# Turning the Tables: Ligand-Centered Hydride Shuttling in Organometallic BIP–Al Systems

**DOI:** 10.1021/acs.inorgchem.5c02587

**Published:** 2025-07-24

**Authors:** Juan Manuel Delgado-Collado, Francisco José Fernández de Córdova, Pilar Palma, Juan Cámpora, Antonio Rodríguez-Delgado

**Affiliations:** Instituto de Investigaciones Químicas, 518805 CSIC-Universidad de Sevilla, Av. Américo Vespucio, 49, Sevilla 41092, Spain

## Abstract

The reversible storage and release of hydride equivalents remains a central challenge in the design of biomimetic redox systems. Cationic 2,6-bis­(imino)­pyridine organoaluminum complexes [(4-R-BIP)­AlR_2_]^+^ (where *R* = H; *R*′ = Me, 1a; *R*′ = Et, 1b; *R* = Bn; *R*′ = Me, 1c) and their neutral 2,6-bis­(imino)-4-R-dihydropyridinate counterparts [(4-R-HBIP)­AlR_2_] 2a-c are presented as chemically reversible hydride exchangers. Interconversion between these systems is achieved through strong reducing agents such as M^+^[HBEt_3_]^−^ (where *M* = Li; Na) or LiAlH_4_, while powerful electrophiles like B­(C_6_F_5_)_3_ or cationic trityl salts Ph_3_C^+^ enable the reverse transformation, with the latter providing complete selectivity. Overall, this reversible hydride exchange mirrors natural NAD­(P)­H/NADP^+^ cofactor system. These findings establish a new platform for ligand-centered hydride shuttling, where the metal fragment acts as a passive modulator—inverting the traditional roles assigned to metal and ligand.

## Graphical abstract

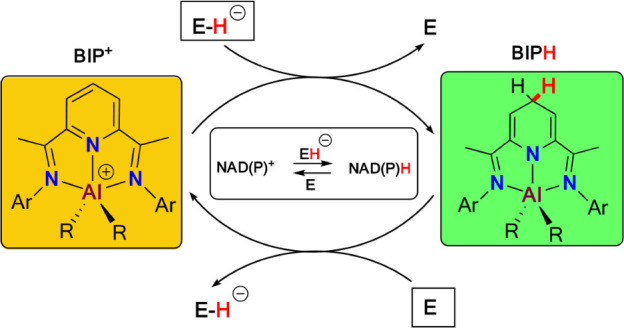

## Introduction

A key strategy for developing more sustainable chemical processes involves drawing inspiration from biological systems.^1^ Reduction and oxidation mediated by well-known nucleotide cofactors, such as nicotinamide adenine dinucleotides (NAD­(P)­H/NAD­(P)^+^),^2^ are among the most prevalent processes in molecular biology.^3^ Many enzymes use nucleotide cofactors to store electrons and protons as “reducing power” in energy-rich systems, which is then utilized for the hydride reduction of organic acceptor molecules, as illustrated in Scheme 1. The cofactor undergoes reversible hydride transfer to and from its organic framework without metal involvement. This capability is due to the mild aromatic stabilization of the pyridinium ring, which can be disrupted and regained at a relatively low energy cost. This transformation is central to various anabolic and catabolic pathways, ranging from photosynthesis to glycolysis.

**Scheme 1:**
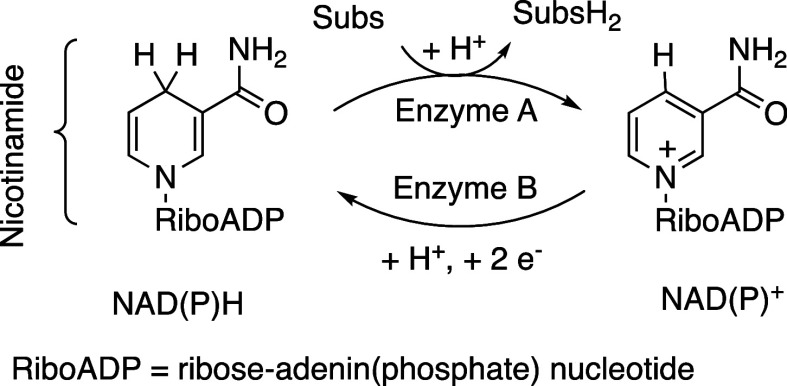
Natural NAD­(P)^+^/NAD­(P)H System^a^

a The (P) notation refers to the (optional) phosphorylation state of the adenine nucleotide unit

Most artificial catalysts for hydrogenation and bond manipulation rely on expensive and scarce platinum-group metals. Consequently, there is significant interest in developing analogous systems based on earth-abundant metals.^4,5^ However, achieving comparable efficiency remains challenging. A useful strategy involves pairing nonprecious metal complexes with organic ligands capable of *metal–ligand cooperation*.^6^ Many such ligands are polydentate chelates, such as the pincer family, and feature pyridine-based rings, which undergo reversible dearomatization during operation.^7^ This behavior resembles that of the NAD­(P)­H/NAD­(P)^+^ system, except that, in such artificial catalysts, the heterocyclic nitrogen is coordinated to a metal fragment. For example, complexes incorporating 2,6-bis­(imino)­pyridine (BIP) ligands frequently undergo redox processes that involve ligand-centered electron transfer and dearomatization of the central pyridine ring.^6d,8a^ These “non-innocent” redox-active ligands have been explored as alternatives to precious metal catalysts, particularly in complexes of first-row transition metals such as iron, cobalt, and manganese.^8a,8b,9^ However, the high reactivity of coordinated BIP ligands often results in complex and irreversible structural transformations, which present significant challenges for their effective use in catalysis.^10^ To mitigate this issue, the conventional acetimidoyl donor groups of traditional BIP ligands have been replaced with nonenolizable benzoimidoyl^11,12^ or 2-pyrazolyl side arms.^13^ While this modification enhances ligand stability, it comes at the cost of more complicated ligand syntheses and, perhaps, some decrease in ligand-centered redox activity. Although Berben has demonstrated the possibility of using such modified BIP-type ligands to exchange hydrogen atoms, including chemical^14^ and electrochemical^15^ catalytic H_2_ production, the ability of the central pyridine ring to mediate reversibly in catalytic hydrogen transfer reactions based—like the NAD­(P)^+^/NAD­(P)H couple—remains largely unexplored.^8c,15b,16^

Over the last two decades, our group has made significant contributions to elucidating the reactivity patterns of BIP ligands, by developing methodologies that isolate fundamental processes. These prevent the occurrence of multiple competing reactions, which often obscure their behavior (see Scheme 2, columns A and B).^10^ Thus, the reaction of BIP ligands with dialkyl complexes, MR_2_ (*M* = Mn,^17a^ Zn,^18a^ *R* = benzyl or allyl), results in the highly selective transfer of one of the R groups to C4 of the pyridine ring, yielding (2,6-bis (imino)-4-alkyl-1,4-dihydropyridinate)-alkylmetal complexes (Scheme 2, column A). The structural similarity of these dihydropyridinates to NAD­(P)H attracted our attention, as they could exhibit a similar functionality as hydride carriers. However, selective alkyl transfer is not general, and extending it to other MR_n_ or even different R groups remains a challenge. For example, Budzelaar reported that the reaction of aluminum alkyls (AlR_3_) with BIP ligands is highly complex due to the low selectivity of alkyl migration and the subsequent transformations of the resulting Al­(III) dihydropyridinate complexes.^19^

**Scheme 2:**
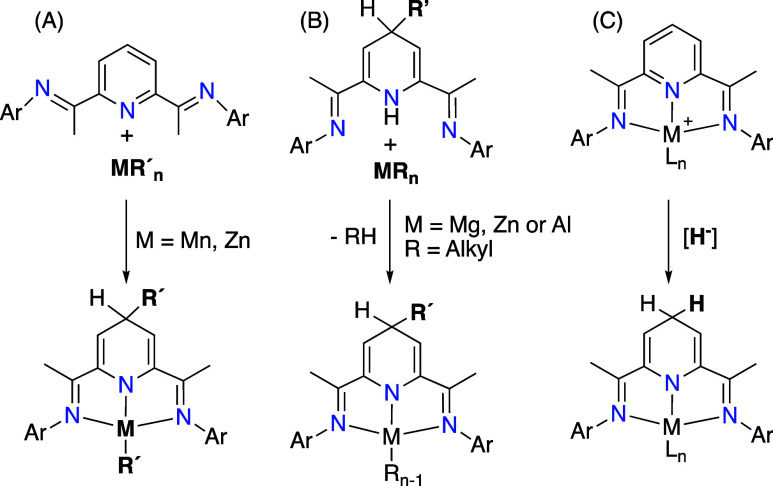
Synthetic Pathways to BIP-based Dihydropyridinate–Metal Complexes

In pursuit of alternative routes to BIP-based dihydropyridinate (4-R-HBIP) complexes, we conducted the demetalation of Mn (II) or Zn­(II) 2,6-bis­(imino)-4-alkyldihydropyridinates obtained via the previously described approach. The resulting free bases were then reacted with Zn, Mg, or Al alkyls (MR′), as shown in Scheme 2, column B.^18,20,21^ This strategy enables the selective and broadly applicable formation of alkylmetal dihydropyridinate complexes, constrained only by the initial choice of the dihydropyridine substituent (i.e., R′), that can be distinct from R.

An alternative approach for the synthesis of these dihydropyridinate-metal species involves dearomatization of the pyridine ring via external hydride attack affording the parent (nonsubstituted) 1,4-H_2_BIP-metal species, as depicted in Scheme 2, Column C, but well-characterized precedents for this process are scarce.^22^ Only two reports describe the dearomatization of pyridine-based pincer ligands coordinated to group 10 (Ni, Pd and Pt)^23^ and Ti,^24^ using Li­[HBEt_3_] as a hydride source in both cases. The only examples of dihydropyridinate formation from coordinated BIP-type ligands involve aluminum complexes, but in both cases, prior reduction of the pyridine ring occurred. These include an intermediate step in the reaction of BIPs with diisobutylaluminum hydride, summarily noted by Budzelaar,^19^ and the well-characterized transformation of a reduced aluminum-TEMPO complex with KH/18C6, reported by Berben, yielding a 1,3-dihydropyridinate.^16^ Notably, reversibility comparable to the NAD­(P)^+^/NAD­(P)H couple has not been demonstrated in any of these precedents.

Although certain hydropyridinate complexes, such as lithium tetrakis­(dihydropyridyl)­aluminate (Lansbury reagent),^25^ are well-established hydride donors,^26^ the hydricity of 4-R-HBIP metal complexes remained hypothetical until 2018. At that time, we demonstrated^18b^ that dihydropyridinates [(4-R-BIPH)­ZnR′] readily transfer hydride to B­(C_6_F_5_)_3_, restoring the aromaticity of the pyridine ring and generating the corresponding cation [(4-R-BIP)­ZnR′]^+^, paired with the [HB­(C_6_F_5_)_3_]^−^ borohydride anion (Scheme 3). We then sought to establish the reversibility of this process—an essential requirement for catalytic applications—by reacting several [(4-R-BIP)­ZnR′]^+^ cations (as salts of an inert tetraarylborate anion) with Na­[HBEt_3_], applying the strategy outlined in Scheme 2C. However, in this case, the borohydride reagent preferentially attacks the metal center, displacing it as Zn­(H)­R′ and leaving the BIP ligand as the corresponding Na^+^ complex. The irreversibility of hydride transfer from [(4-R-BIP)­ZnR′]^+^ can be ascribed to the Lewis acidity of the pseudosquare-planar Zn center.^27^

**Scheme 3:**
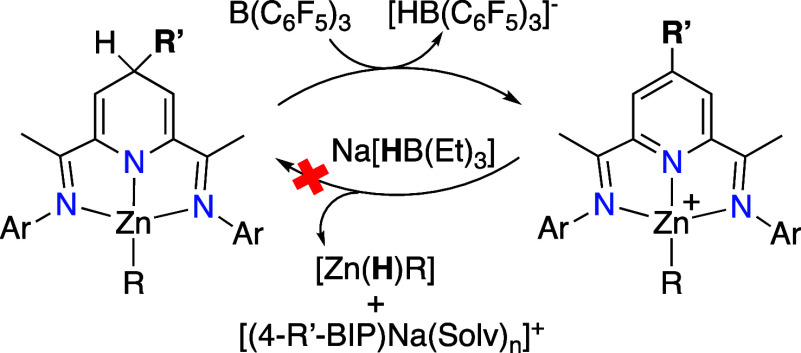
Irreversible Hydride Transfer from Zn­(II) 1,4-Dihydropyridinates

Recently, we synthesized a series of pentacoordinated aluminum cations, [(BIP)­AlR_2_]^+^ (**1**), by reacting trialkyl aluminum compounds (AlR_3_) with the acidic salts [H­(BIP)]^+^ X^–^, where X^–^ is a weakly coordinating anion.^28^ These cations undergo reversible electrochemical or chemical one-electron reduction at the BIP ligand, leading to paramagnetic derivatives while exhibiting remarkable tolerance to atmospheric oxygen and moisture that contrasts sharply with the well-known air sensitivity of most organoaluminum compounds. This exceptional stability likely stems from the suppression of Lewis acidity at the pentacoordinated Al center. Thus, we reasoned that such cations could be ideal candidates to exhibit the reversible hydride transfer behavior we strive for.

In this work, we build upon our previous studies on zinc BIP complexes to develop a chemically reversible hydride exchanger based on organoaluminum compounds. We demonstrate that the readily available cations **1** selectively accept hydride from suitable donor reagents, enabling—for the first time—a rational approach to synthesizing the parent hydropyridinates, i.e., lacking alkyl substitution on the pyridine ring. Conversely, the [(HBIP)­AlR_2_] hydropyridinate complexes (**2**), whether obtained through previously reported methods or as 4-alkyl derivatives formed via the dearomatization of **1**, exhibit reactivity analogous to their Zn counterparts, transferring hydride to electrophiles such as trityl (CPh_3_^+^) and B­(C_6_F_5_)_3_. These findings establish organoaluminum BIP derivatives as reversible hydride exchangers, closely resembling the NAD­(P)­H/NAD­(P)^+^ couple. This breakthrough provides a promising blueprint for designing biomimetic catalytic hydrogen transfer systems.

## Results and Discussion

As outlined in the Introduction, this study aims to demonstrate the ability of the BIP-based complexes to function as hydride carriers. Coordinatively saturated complexes could accept hydride in the ligand from strong donors forming hydropyridinate species that, in turn, would deliver hydride selectively to acceptors, mimicking the biological role of NAD­(P)^+^ cofactors. Since [(BIP)­AlR_2_]^+^ cations (**1**, see Chart [Fig chart1]) are readily available from AlR_3_, and the protonated H­(BIP)^+^X^–^ ligand salts,^28^ we began the study with these simple cations, setting *R* = Me or Et (**1a** and **1b**, respectively), and X^–^ to PF_6_^–^ or BAr^F^_4_^–^. To reduce variability, we restrained the *N-*aryl substituent on the imine to 2,6-diisopropylphenyl (this ligand is usually abbreviated as ^DiPP^BIP, hereafter as BIP for simplicity). In addition, we applied the same procedure to prepare one additional cation, **1c**, similar to **1a** but containing a modified BIP ligand with a benzyl substituent in position 4 of pyridine. To distinguish this ligand from its nonsubstituted analog, it will be abbreviated as ^Bn^BIP. Complex **1c·BAr** ^**F**^_**4**_ has been fully characterized, including a SC XRD (see Figure 38 in the SI).

**1 chart1:**
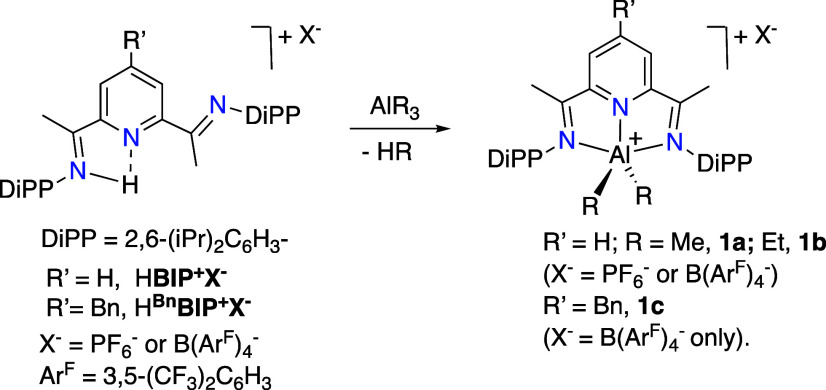
Synthesis of Cationic Complexes of Type **1** used as Starting Materials in this Work

### Reversible Hydride Exchange on Nonsubstituted BIP Complexes

Mixing cold (−30 °C) solutions containing equimolar amounts of one of the four ionic complexes **1a,b·PF**_**6**_ or **1a,b**·**BAr** ^**F**^_**4**_ and lithium superhydride instantly triggers a dramatic color change from off-yellow to deep turquoise. As the mixture warms to room temperature, its color gradually shifts to dark burgundy, characteristic of reduced BIP complexes. In contrast with the Zn system, where superhydride displaces the cationic metal fragment from the BIP ligand, the ^1^H NMR spectra of the crude mixtures reveal a clean transformation of the starting complexes **1a,b** into the neutral dihydropyridinates [(4-H_2_BIP)­AlR_2_], **2a** or **2b** (Scheme 4). The counteranion (PF_6_^–^ or BAr^F^_4_^–^) has little or no influence on the course of the reaction.

**Scheme 4:**
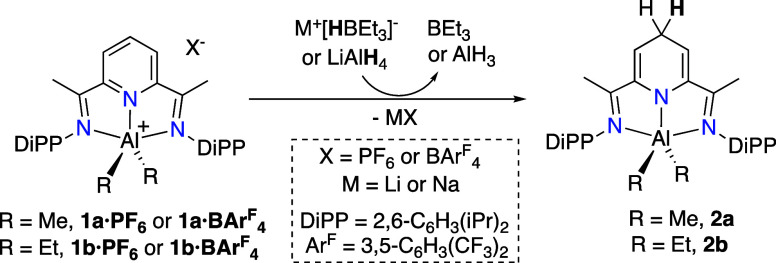
Reaction of Nonsubstituted Cations **1a**,**b** with Strong Hydride Donors

To assess whether the choice of alkali cation influences the course of the reaction, we carried out similar experiments using sodium instead of lithium super hydride, with essentially the same results. However, purifying the products from the sodium salts proved slightly more cumbersome in practice. Consequently, we focused on lithium reagents for hydride transfer.

The [HBEt_3_]^−^ anion selectively delivers hydride to C4 of the heterocycle. This is evident from the symmetrical NMR spectra, which show only one set of imidoyl signals and a simple^1^H spin system for the dihydropyridine ring. The latter shows two triplets of the same intensity at ca. 3.5 and 4.9 ppm, giving rise to the equivalent 3,3′–C*H* methynes and the C(4)*H*_*2*_ methylene, with^3^ *J*_HH_ = 4.0 Hz, for both **2a** and **2b**. Similar ^1^H NMR features have been reported for complexes Al^13a^ and Rh^29^ complexes containing 4-hydropyiridinate moieties, and the group 10 PONOP pincers mentioned in the Introduction (Scheme 2C).^23^

After removing salts, complexes **2a** and **2b** were isolated in high yields (83–89%) as purple solids. As shown also in Scheme 4, a similar result was obtained when **1a·PF**_**6**_ was treated with LiAlH_4_. The isolated yield was slightly lower (71%); thus, the procedure was not extended to the four starting materials. Complexes **2** were fully characterized with the usual ensemble of spectroscopic techniques (^1^H and ^13^C NMR spectra) and elemental analyses. In addition, the characterization of **2b** was completed with its SC XRD (Figure 1).

**Figure 1:**
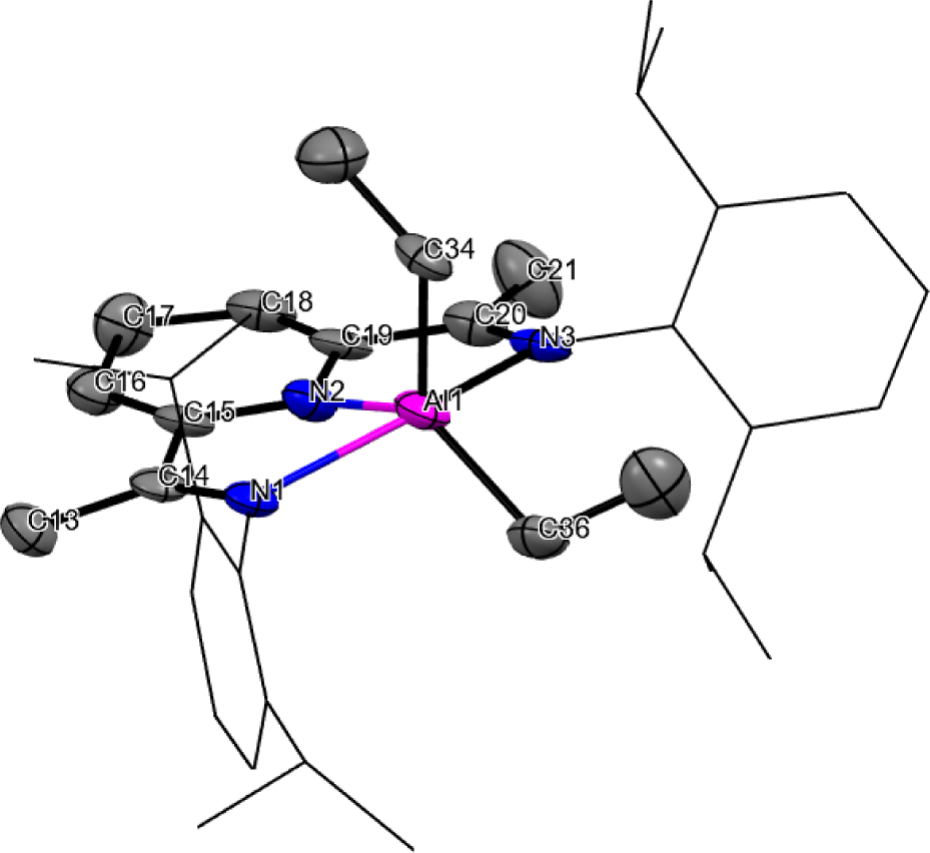
ORTEP representation of the structure of compound **2b**. Hydrogen atoms have been omitted for clarity. Selected bond lengths (Å) and angles (deg): Al–N(1): 2.218(5); Al–N(2): 1.893(5); Al–N(3): 2.238(5); Al–C(36): 1.993(8); Al–C(34): 1.981(6); N(1)–C(14): 1.274(7); N2–C(15): 1.383(7); C(14)–C(15): 1.451(8); C(13)–C(14): 1.515(8); C(15)–C(16): 1.346(9); C(16)–C(17): 1.485(9); N(2)–Al–N(1): 76.0(2); N(2)–Al–C(36): 136.3(2); N(2)–Al–C(34): 112.7(2); C(34)–Al–C(36): 111.0(3); N(2)–Al–N(3): 76.5(2); N(1)–Al–N(3): 150.9(2).

This is the first time that “parent” dihydropyridinate complexes of type **2** are synthesized through a rationally devised protocol. However, a few relevant SC XRD s,^15a,20,30^ have been reported in the literature, including a low-quality structure of their Al­(*i*-Bu)_2_ analog, determined from a small amount of crystalline material obtained from the reaction of the BIP ligand with Al­(H)­(*i*-Bu)_2_.^19^ The molecular structure of **2b** is unexceptional and shares its key features with these precedents. Thus, it shows a slightly puckered dihydropyridine ring (puckering angle between planes C16,C17,C18 ∧ C18,C17,N2,C16,C15 ≈ 5 deg.) and a distinct alternation of C–C bond-lengths, as expected for a localized, nonaromatic system. The intraligand C–C and C–N distances are identical to those found in the previously reported [(4-allyl-HBIP)­AlMe_2_][Bibr ref20] within experimental accuracy. These values are also closely comparable to those observed in the related complexes [(4-benzyl-HBIP)­MR′] with *M* = Zn (R′ = benzyl)^18a^ and Mg (R′ = *n*-butyl).^18b^ The coordination environment in **2b** is pentacoordinated, with a somewhat distorted square-planar geometry, as indicated by a τ_5_ index^31^ of 0.24. This falls between those of the mentioned [(4-allyl-HBIP)­AlMe_2_] and the noninnocent [(BIP**·**)­AlEt_2_] complexes,^28^ where the geometries are highly distorted (τ_5_ ≈ 0.43) and nearly ideal bipyramidal trigonal (τ_5_≈ 0.04), respectively.

We previously reported that BIP scaffolds impart remarkable chemical stability to their organoaluminum derivatives, despite the inherent reactivity of Al–C bonds. Cationic compounds of type **1** exhibit a surprising resistance to mild protic acids, such as alcohols or water, and even tolerate air exposure in solution.^28^ The dihydropyridinates **2a,b** are also quite stable, considering the high reactivity of the dihydropyridyl(−) fragment as a reductant amido ligand. They also withstand exposure to normal alcohols like MeOH, ^i^PrOH or BnOH, even if these are added in a moderate excess. (See Figure S36 in Section 3.8, for the reaction of **2a** with MeOH monitoring) However, these complexes show an interesting reactivity. Thus, whereas they are stable for days in benzene, toluene, or dichloromethane at room temperature, heating induces competitive dimerization and acceptorless dehydrogenation, analogously to their 4-alkyl substituted counterparts.^20^ This process will be described in more detail in a forthcoming article. In addition, these compounds react slowly with air at room temperature. Unlike complexes **1,** solutions of **2a** in C_6_D_6_ slowly decompose when exposed to air in a gastight NMR tube. After 24 h, the signals of the starting material had lost 45% of their original intensity, with concomitant release of the aromatized BIP ligand. A light white insoluble solid precipitated, presumably a polymeric alumoxane akin to MAO. The same products (free BIP and a white precipitate) were rapidly formed when dry oxygen gas was carefully bubbled into the sample, as its deep burgundy color gradually changed to a lighter hue. This indicates that the reaction with air does not involve hydrolysis but hydride transfer from the dihydropyridinate to O_2_. (See Figure S35 in Section 3.7)

The chemical stability of complexes **2a** and **2b** facilitates the exploration of their capacity as hydride donors. By analogy with our previous work with Zn­(II) complexes, we initiated this study using B­(C_6_F_5_)_3_, a relatively strong Lewis acid able to abstract hydride from the pyridine ring. The expected products were cations **1a** and **1b,** respectively, paired with the [HB­(C_6_F_5_)_3_]^−^ borohydride anion. These ion pairs would be close analogs of the reactive combination **1a,b**/[HBEt_3_]^−^ studied above. However, in contrast with the clean and selective reaction of B­(C_6_F_5_)_3_ with zinc dihydropyridinates [(4R-HBIP)­ZnR′],^18b^ the reactions with **2a** and **2b** proceed without complete selectivity, producing deeply colored reaction mixtures whose NMR spectra indicate the formation of significant amounts of side products. (See reaction monitoring S21–S29 in Sections 3.1–3.3)

Thus, when **2a** or **2b** are treated with an equimolar amount of B­(C_6_F_5_)_3_ in cold (−30 °C) CD_2_Cl_2_, an instant color change occurs, from purple to a brighter tone that persists on warming. Their ^1^H NMR spectra are consistent with cations **1**, complicated with additional signals, which could not be easily assigned due to extensive overlap. The ^11^B and ^19^F spectra, much simpler, indicate the presence of a mixture of the expected borohydride [B­(H)­(C_6_F_5_)_3_]^−^ and alkylborate [B­(R)­(C_6_F_5_)_3_]^−^, (*R* = Me or Et) suggesting that H/R exchange takes place between the boron and aluminum centers. Whereas this reaction is discussed later in detail, at this point, we chose to circumvent this issue by replacing B­(C_6_F_5_)_3_ with the isoelectronic C-based cation [CPh_3_]^+^, as [B­(C_6_F_5_)_4_]^−^ salt, assuming that this anion would not undergo side H/R exchange reactions, since it does not contain the hydride to be exchanged as this (H^–^) was expected to form nonpolar solvent soluble triphenylmethane (HCPh_3_) easily removable by washing with hexane or pentane in which triphenylmethane is highly soluble. As anticipated, **2a** and **2b** react cleanly with one equivalent of the trityl salt in cold dichloromethane, returning the corresponding cations **1a** and **1b** in quantitative spectroscopic yield (Scheme 5). The salts **1a·B­(C**_**6**_ **F**_**5**_)_**4**_ and **1b·B­(C**_**6**_ **F**_**5**_)_**4**_ were isolated in good yields as powdery materials (81 – 85%) that, on extensive washing with pentane and recrystallization, lose their intense red color, becoming pale pink or orange crystalline solids. Their ^1^H NMR spectra are virtually identical to those of the corresponding [BAr^F^_4_]^−^ salts used as starting materials in this work, except for the absence of counteranion signals. The identity of **1b·B­(C**_**6**_ **F**_**5**_)_**4**_ was further validated by determining its SC XRD (Figure 2). The configuration and metric parameters for **1b** are indistinguishable from those reported recently for the same cation, characterized as the [PF_6_]^−^ salt.^28^ This result closes the cycle, demonstrating the ability of the BIP ligand to act as a reversible hydride carrier when the appropriate conditions are met.

**Scheme 5:**
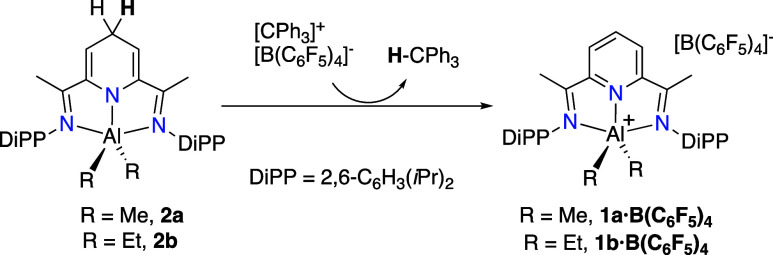
Regeneration of Cations **1** from Dihydropyridinates **2** with a Trityl Salt

**Figure 2:**
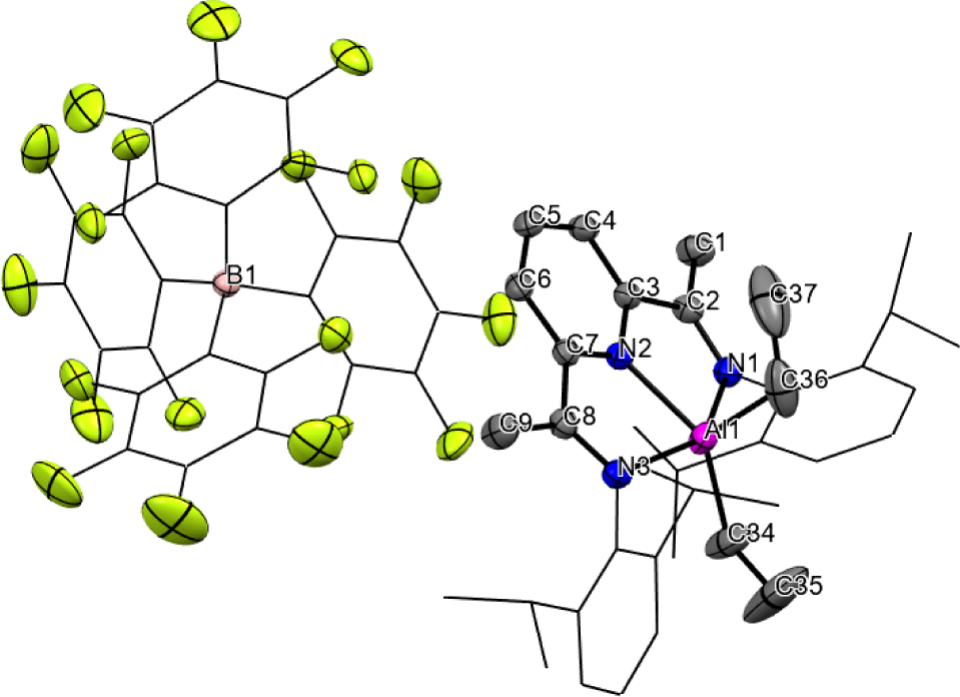
ORTEP representation of the structure of compound **1b·B­(C**_**6**_ **F**_**5**_)_**4**_. Hydrogen atoms and 2.5 molecules of CH_2_Cl_2_ have been omitted for clarity. Selected bond lengths (Å) and angles (deg): Al–N(1): 2.2028(16); Al–N(2): 2.0081(16); Al–N(3): 2.1733(16); Al–C(36): 1.968(3); Al–C(34): 1.970(3); N(1)–C(2): 1.282(2); N2–C(3): 1.342(2); N(2)–C(7): 1.342(2); N(3)–C(8): 1.283(2); C(1)–C(2): 1.493(3); C(3)–C(2): 1.488(3); C(3)–C(4): 1.384(2); C(4)–C(5): 1.384(3); C(5)–C(6): 1.387(3); C(6)–C(7): 1.389(3); C(7)–C(8): 1.487(3); C(8)–C(9): 1.486(3); N(2)–Al–N(1): 74.73(6); N(2)–Al–C(36): 102.30(11); N(2)–Al–C(34): 139.64(11); C(34)–Al–C(36): 117.99­(14); N(2)–Al–N(3): 75.25(6); N(1)–Al–N(3): 147.22(7).

Next, we set to identify the minor products formed in the reactions of **2a** and **2b** with B­(C_6_F_5_)_3_. As mentioned, this is difficult to ascertain from the^1^H or ^13^C­{^1^H} NMR spectra of the crude mixtures, but their ^11^B and ^19^F NMR spectra indicate that the minor products arise from H/R exchange (see Sections 3.1–3.3). For example, the ^11^B spectrum of the mixture arising from **1a** and B­(C_6_F_5_)_3_ at −30 °C shows two sharp signals in a 3:1 ratio. The major resonance, at −25.3 ppm, splits in a broad doublet in the ^11^B spectrum^1^ *J*_BH_ = 93 Hz), characteristic of the [B­(H)­(C_6_F_5_)_3_]^−^ anion,^32^ whereas the minor signal at −14.9 ppm does not show ^1^H–^11^B coupling, indicating the absence of a B–H bond. The ^19^F spectrum of the same mixture also shows two sets of closely spaced signals in 3:1 ratio, corresponding to the three types of fluorine in the C_6_F_5_ groups (in CD_2_Cl_2_: Major, −133.9, *o*-F; −164.4, *p*-F; −167.4 ppm, *m*-F; Minor, −133.1 *o*-F; −165.1, *p*-F; −167.8 ppm, *m*-F). Comparing the chemical shifts of these signals with literature data confirmed the identity of the major species as the [B­(H)­(C_6_F_5_)_3_]^−^ anion^32^ and allowed us to assign the minor to methylborate,^33^ [B­(Me)­(C_6_F_5_)_3_]^−^. This conclusion is further supported by the observation of a low-intensity, broad singlet at 0.42 ppm in the ^1^H spectrum, corresponding to the B-Me group. This compares well with the data reported for other ionic complexes with the same anion.^34^ Analogously, the ^11^B and ^19^F spectra of the mixture arising from **2b** also show two signal sets, this time in approximately 8:1 ratio. Again, the major species corresponds to [B­(H)­(C_6_F_5_)_3_]^−^, and the slightly differently shifted set of the minor (^19^F in CD_2_Cl_2_: −132.5, *o-*F; −165.1, *p*-F; −167.8 ppm, *m*-F) is assigned to ethylborate, [B­(Et)­(C_6_F_5_)_3_]^−^, also consistent with the ^1^H literature data (in CD_2_Cl_2_).^35^ In the ^1^H spectrum, the C*H*_3_ signal of the B-*Et* anion, a small triplet at 0.54 ppm, was also observed, despite its very low intensity.^34^

The formation of methyl- or ethylborate along with borohydride anions in the reactions of **2a** or **2b** with B­(C_6_F_5_)_3_ dictates that the cationic side products, labeled as **1′a** and **1′b**, must contain only one alkyl group attached to the Al center, as shown in Scheme 6 (top). As mentioned above, the precise identity of these organometallic byproducts is difficult to establish directly from the NMR spectra, due to their low concentration and extensive signal overlapping. While this is especially true for **1′b**, we realized that the resolution of the ^1^H spectra of the richer **1** *a* **/1′a** mixture is better in C_6_D_6_ than in CD_2_Cl_2_. This enabled us to distinguish a few critical spectroscopic features of the minor component (see Figures S24 and S21, respectively, in Sections 3.2 and 3.1 in the SI).

**Scheme 6:**
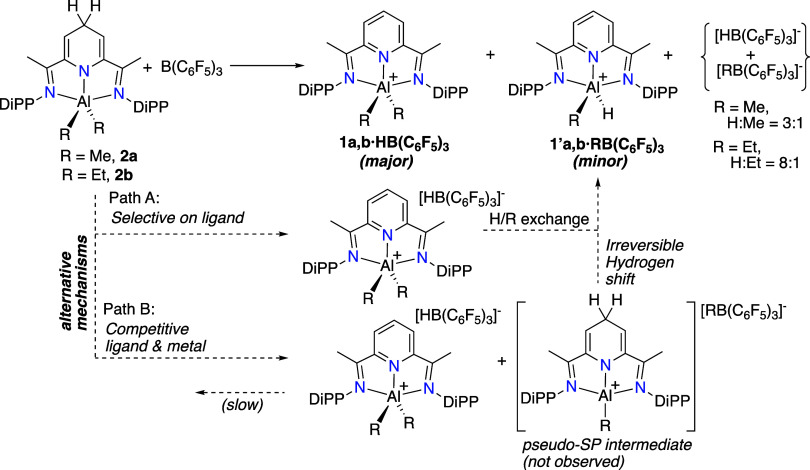
Reaction of the Dihydropyridinate Complexes **2a** and **2b** with B­(C_6_F_5_)_3_, and Two Alternative Mechanisms to Explain the Formation of Byproducts

First, the overall intensity of the **1′a** subspectrum is in good agreement with the 3:1 ratio of the borohydride and methylborate anions, deduced from the ^11^B and ^19^F spectra. In the low field edge of the spectrum of **1′a**, a well-resolved pattern formed by a doublet and triplet at 7.44 and 7.78 ppm, in 2:1 intensity ratio, indicates that **1′a** has an aromatic pyridine core. The opposite edge of the spectrum shows another significant feature. A small signal of an Al-bound methyl of **1′a** was found at −0.82 ppm, slightly shifted upfield from the main Al*Me*_2_ signal of **1a** (−0.76 ppm). The former has about the correct integral value for a single Me group attached to aluminum.

Second, two characteristic methyne septets at 2.39 and 2.85 ppm (2H each), bespeak the diastereotopic relationship of the *ortho i-*Pr substituents of the N-DiPP groups. This is consistent with a symmetry loss at the Al center, caused by replacing one methyl group, likely for an H coming from borohydride anion. Though we have been unable to locate a ^1^H signal for the Al-bound H atom (expected as a broad, low intensity signal in the central region of the spectrum), the spectral data as a whole support the proposed structure for **1′a** as a mixed (hydrido)­methyl complex, and, by extension, as a (hydrido)­ethyl derivative for **1′b**. Since **1a·[B­(H)­(C**_**6**_ **F**_**5**_)_**3**_] and **1′a·[B­(R)­(C**_**6**_ **F**_**5**_)_**3**_] are isomers, both crude solid mixtures provide correct elemental analyses for their expected composition.

Two possible mechanisms can explain the formation of **1′a** and **1′b**, as shown in the lower part of Scheme 6. Path A entails selective B­(C_6_F_5_)_3_ attack on the 4-HBIP ligand of **2**, followed by alkyl/hydride exchange at the metal center. In Path B, borane can attack competitively either the 4-HBIP or the AlR_2_ site, leading to two cationic products: **1**, and a transient intermediate, which rapidly rearranges to **1′**. This intermediate contains a nucleophilic 1,4-dihydropyridinate ring, and a highly distorted four-coordinate Al center with pseudosquare-planar (pseudo-SP) geometry imposed by the pincer-like ligand. A cationic Al­(III) center in such a coordination environment should exhibit significant Lewis acidity,^36^ and, therefore be strongly driven to undergo an H atom shift to rearrange into the more stable, five-coordinated, **1′**. The migration of the hydrogen atom from the ligand could occur via an intra- or, more likely, intermolecular pathway. In this mechanism, the **1**/**1′** selectivity becomes imprinted on the product ratio at the moment of the borane attack, which is essentially instant even at low temperature. In contrast, path A would initially yield **1**, and this would evolve into the **1/1′** mixture at a later stage.

Further observations support mechanism B, rather than A. Thus, mixing solutions **2a** with B­(C_6_F_5_)_3_ in CD_2_Cl_2_ at −40 °C, and immediately recording the ^19^F NMR spectrum shows similar anion ratios than the preparative procedure (i.e., borohydride/alkylborate = 3.4:1 for **1a**/**1′a**). This indicates that the products are generated within seconds or minutes after mixing. Therefore, the initial hydride/alkyl abstraction ratio is determined under kinetic control, which is readily explained by mechanism B, but less easily with mechanism A. The latter would require that the H/R exchange at the Al center should be at least as fast, or even faster, then hydride abstraction from the ligand to become competitive. Allowing the crude samples arising from **2a** and B­(C_6_F_5_)_3_ to stand at room temperature for 29 h led to a slight but significant decrease of the initial borohydride:methylborate ratio, from 3.4:1 to ca. 1.2:1, concomitant with a similar evolution of the **1a:1a′** signals in the ^1^H NMR spectrum. This is much slower than the reaction of **2a** with the borane, ruling out that the initial product ratio could arise as shown in Path A. Path B is also more consistent with the higher borohydride/ethylborate ratio observed for **2b** since the Al-Et bond is less accessible to the bulky B­(C_6_F_5_)_3_ than the Al-Me of **2a**. Thus, we expect that bulkier R′ groups (or more stable Al-R′ bonds) would minimize the alkyl competition pathway.

Most likely, the slow H/R exchange does not proceed via metathetical H–B/Me-Al exchange, as Path A suggests, since both the boron and aluminum centers are coordinatively saturated. Instead, this process could follow pathway B, involving a reversible hydride exchange step. Although [B­(H)­(C_6_F_5_)_3_]^−^ is a weak hydride donor, it could still undergo reverse hydride transfer,^37^ enabling methyl abstraction by the resulting B­(C_6_F_5_)_3_. This leads to the observed slow thermodynamic drift of the product ratio. Mild heating of the reaction mixture at 55 °C promotes further exchange, reaching a **1a:1′a** 1:3 after 24 h. However, conversion slows drastically hereafter, keeping with the expected second-order kinetics. The progression of this secondary exchange process indicates that **1′a** is the thermodynamically favored product and also suggests that the hydride shift step is irreversible.

The reducing capacity of a hydride donor can be quantified by its “hidricity” (ΔG°_H_), which corresponds to the Gibbs free energy associated with hydride dissociation from its conjugate pair (i.e., the reaction (AH)^n^ → A^n+1^ + H^–^, where A^n+1^ is a Lewis acid with an n+1 electric charge). Hydricity values can be determined by experimental or computational methods and have been reported for many organic^38^ and organometallic^37,39^ molecules, typically in a polar solvent like acetonitrile. These data can serve a similar role to the p*K*_a_ of protic acids to characterize the Lewis acid strength of hydride acceptors: For example, trityl is a much stronger acid than B­(C_6_F_5_)_3_ because the hydricity of the corresponding conjugate, triphenylmethane (CHPh_3_, 92 kcal/mol) is ∼27 kcal/mol more positive than that of [B­(H)­(C_6_F_5_)_3_]^−^ (65 kcal/mol). Our reaction data allow estimating the reducing capacity of dihydropyridinates of type **2.** Complexes **1** are quantitatively reduced to **2** by Li­[B­(H)­(Et)_3_]^−^ or LiAlH_4_ (ΔG°_H_- = 26 and 43 kcal/mol, respectively), implying that the ΔG°_H_- of **2** must be at least >43 kcal/mol to ensure a favorable Gibbs energy balance with both reagents. Conversely, complexes **2** transfer hydride to CPh_3_^+^ and, despite competition with Me transfer, also to the weaker acceptor B­(C_6_F_5_)_3_ (ΔG°_H_- ≈ 92 and 65 kcal/mol, respectively). This places an upper bound for the hydricity of **2** at <65 kcal/mol leading to an estimated value between 43 and 65 kcal/mol, or 54 ± 11 kcal/mol. Similar estimation has been used by Berben with Group 13 ion coordination to pyridyls, scaling hydricity for dihydropyridinates.^40^ Their reducing capacity place these compounds in an interesting spot, higher than conventional organic hydride donors,^39^ e.g., triarylmethanes (ca. 120–76 kcal/mol), fluorenes (114–70 kcal/mol) or dihydropyridine derivatives (70–61 kcal/mol, including NAD­(P)­H, 77.1 kcal/mol), but comparable to many transition metal hydrides.

Complexes of type **2** are unique in their ability to react simultaneously with Lewis acids as organic hydride donors or as classic organometallic alkyls. However, the selectivity observed is intriguing, assuming that the CPh_3_^+^ and B­(C_6_F_5_)_3_ reactions involve direct electrophilic attack at carbon (the dihydropyridine 4-CH_2_) or at the metal center. The trityl cation is known to be a significantly stronger Lewis acid than B­(C_6_F_5_)_3_, as indicated by the significantly more positive ΔG°_H_- of the former.^38^ Although the dearth of thermochemical data in the literature for alkyl abstraction processes^41^ prevented us from estimating the analogous parameter (ΔG°_R_) for R^–^ removal from the AlR_2_ unit of **2a** or **2b** (*R* = Me or Et) with trityl and B­(C_6_F_5_)_3_, we do not expect a different trend in these reactions, i.e., we expect trityl to be a significantly stronger R^–^ abstraction reagent. However, based on the Evans–Polanyi principle, one would anticipate that the milder electrophile B­(C_6_F_5_)_3_ might exhibit a higher selectivity than the “hot” trityl cation. Steric effects also fail to account for the observed selectivity trend: the B­(C_6_F_5_)_3_ boron atom, significantly more crowded than the carbon center in CPh_3_^+^, would be expected to attack more slowly on the sterically hindered AlR_2_ center than on the relatively exposed 4-CH_2_ of the dihydropyridine ring. On these premises, we tentatively believe that the main factor directing the selectivity of the trityl cation is its positive electric charge. The electropositive Al center bears a partial positive charge. Thus, the trityl cation finds an extra electrostatic repulsion barrier to overcome that the borane does not find. This suggests that using cationic electrophiles should enhance the selectivity for ligand-centered attack.

#### Hydride Exchange on 4-Benzyl-BIP Complexes

Since hydride transfer to the cationic Al complexes **1a** and **1b** invariably involves position C4 of the pyridine ring, we sought to determine whether the same selectivity holds for 4-alkyl-pyridine analogs. To this end, we investigated the benzyl-substituted derivative **1c.** The reaction of **1c·BAr** ^**F**^_**4**_ with one equivalent of Li­(HBEt_3_) in cold CH_2_Cl_2_ causes a different color change: from reddish to deep royal blue, suggesting a different reaction than that with the unsubstituted derivatives **1a** and **1b**. After removing triethylborane and lithium salts, the ^1^H NMR spectrum in C_6_D_6_ revealed two isomeric hydropyridinate products in ∼1:5 ratio, **2c** and **3c**, corresponding to competitive hydride transfer to the C4 and C3 positions of the pyridine ring (Scheme 7). The structures of the isomers are unambiguously ascertained from the signals from the 4-benzylhydropyridine fragments (see the experimental section and SI for details).

**Scheme 7:**
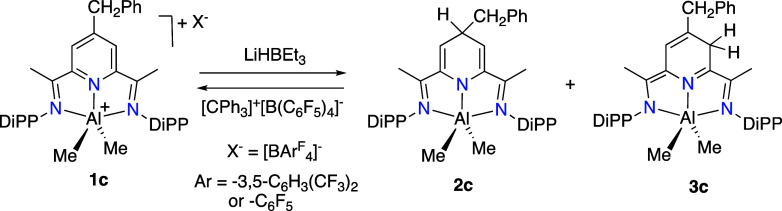
Reversible Hydride Addition to the Cationic ^Bn^BIP Derivative **1c**

The minor isomer, **2c**, corresponds to the known complex^20^ previously obtained as a pure isomer via the reaction of the free 4-Bn-BIPH_2_ base with AlMe_3_, as shown in Scheme 2B. Its 4-benzylhydropyridinate subspectrum exhibits three multiplets, in 2:1:2 ratio, at 2.84, 4.06, and 5.07 ppm, corresponding to the benzyl CH_2_, the 4-C­(Bn)*H* and the equivalent *sp* ^2^–C*H* in the ring positions 3 and 5. The major product, **3c**, also features three singlets at 3.14, 3.35, and 6.01, in 2:2:1 ratio, corresponding to the *sp* ^3^-methylene groups (benzyl and the C(3)­H_2_) and the *sp* ^2^-methyne C5–H of a 4-benzyl-3-hydropyridinate, respectively. As expected, the AlMe_2_ fragments of the isomers give rise to two singlets for **2c** (−0.61 (3H) and −0.62 (3H) ppm) and one singlet at −0.52 (6H) ppm for **3c** in a 1:5 ratio, respectively.

The above experiments demonstrate that alkylation at C4 of the pyridine ring does not significantly alter the chemical behavior of the organoaluminum BIP complexes. However, the presence of an alkyl substituent only perturbs the hydride transfer regioselectivity, shifting the preferred site to hydride attack from C4 to C3. The regioselectivity shift is relatively small: a 5:1 isomeric ratio implies less than 1 kcal/mol in Δ*G* ^‡^ under the experiment conditions, consistent with the moderate steric hindrance of the benzyl substituent. Notably, Berben has shown that the regioselectivity for hydride attack at a BIP-Al complex fully shifts to position 3 when the oxidation state of the ligand is previously reduced by two units (BIP + 2e^–^).^16^ In this case, the effect arises from electronic effects rather than steric hindrance. However, the increased electron density on the doubly reduced ligand significantly diminishes its reactivity toward nucleophilic attack, and the hydride addition (using KH) proceeded in low yield (18%).

Interestingly, the isomerism on the dihydropyridinate complexes has little impact on their reactivity as competent hydride donors, as both **2c** and **3c** act similarly to their counterparts **2a** and **2b**. Treatment of the isomeric mixture of dihydropyridinates with a stoichiometric amount of [CPh_3_]­[B­(C_6_F_5_)_4_] cleanly regenerates the cation **1c**, now paired with the [B­(C_6_F_5_)_4_]^−^ anion, as shown in Scheme 7. The product, **1c·B­(C**_**6**_ **F**_**5**_)_**4**_, was isolated in excellent yield (see Experimental). Similar experiments were conducted by reacting the **2c**/**3c** mixture with B­(C_6_F_5_)_3_ (see NMR monitoring of the reaction in Figures S32–S33 in Section 3.5) to assess whether competitive H/Me abstraction persists in this system. The ^11^B and ^19^F NMR spectra of the reaction mixture revealed a 1:3 mixture of [B­(H)­(C_6_F_5_)_3_]^−^ and [B­(Me)­(C_6_F_5_)_3_]^−^ anions, suggesting that the steric hindrance of the 4-Bn substituent shifts the preferred abstraction regioselectivity from the ring to the metal.

The ^1^H spectrum of this mixture indicates the formation of the expected products, **1c** and **1′c**. This latter shows similar spectral features to those of **1′a** and **1′b**, i.e., aromatic pyridine, a single Al-Me group, and desymmetrization of the Al center, revealed by the diastereotopic differentiation of the DiPP *i*Pr groups. Notably, however, the **1c**:**1′c** product ratio is 1:1 across several independent experiments, which is inconsistent with the above-mentioned anion distribution observed in the ^11^B and ^19^F spectra. Allowing the mixture to evolve at room temperature only increased the mismatch: after 24 h, the ^11^B and ^19^F signals of the borohydride anion become almost undetectable, whereas the **1c**:**1′c** ratio only increases to 1:2. After 48 h, the borohydride signals vanish entirely from the NMR spectra, leaving those of the [MeB­(C_6_F_5_)_3_]^−^ anion alone. Yet, in the ^1^H spectrum, the **1c**:**1′c** ratio only increases slightly, from 1.20 to 1.25. The apparent “excess” of [MeB­(C_6_F_5_)_3_]^−^ cannot be explained unless a fraction of the starting Al (**2c** + **3c**) is converted into NMR-silent paramagnetic species.

Although, so far, we have been unable to isolate paramagnetic products, the EPR spectrum of the reaction mixture consistently shows a signal with a complex hyperfine structure at g ≈ 2.004 (see Figure S34), reminiscent of those of neutral complexes [(BIP)­AlR_2_].^28^ We propose that this species might arise via Al-Me/B–H exchange, as detected with unsubstituted **2a** or **2b**. However, whereas the [HB­(C_6_F_5_)_3_]^−^ seemingly does not attack the unsubstituted BIP ligand of the mixed alkyl­(hydrido) cations **1′a** or **1′b**, it does react further with **1′c** to afford unstable hydropyridinate intermediates, that evolve via aceptorless dehydrogentation^17b,20^ into paramagnetic compounds. This behavior suggests that, under the thermodynamic control conditions imposed by the use of B­(C_6_F_5_)_3_ as hydride acceptor, the influence of the 4-benzyl substituent on the reactivity of the pyridine ring results in a qualitatively different outcome, ultimately leading to complete H–B/Me-Al exchange and partial reduction of the BIP complexes into NMR-silent paramagnetic species.

## Conclusions and Outlook

This study demonstrated for the first time that BIP-based complexes can exhibit perfectly reversible hydride transfer, resembling the natural NAD­(P)­H/NAD­(P)^+^ cofactor system. The process relies on the dearomatization and aromatization of the pyridine ring, without the active participation of the metal center. However, the presence of the electropositive metal fragment significantly enhances the reducing power of the hydropyridinate form, positioning its thermodynamic hydricity between that of organic hydride donors and metal-based hydrides. Notably, the hydride transfer selectivity is sensitive to the pyridine ring substitution, as alkylation at C4 shifts the preferred attack site from carbon 4 to the 3(5) positions without compromising reversibility. This feature adds flexibility to these complexes as hydride shuttles.

A key feature of these aluminum complexes is the coordinative saturation of the pentacoordinated Al center. The kinetic inertness of the alkyl ligands—which arises from the covalent, nondissociable nature of the Al–C bonds and the absence of localized electron pairs—renders the organometallic Al fragment largely passive, despite the intrinsic reactivity of the Al–C bonds. Consequently, the role of the metal is confined to that of a stereoelectronic modulator, inverting the usual relationship between metal and ligands in homogeneous catalysis. This insight suggests that replacing the AlR′_2_ unit with other organometallic fragments could provide a versatile strategy to tune ligand-centered reactivity and selectivity, enabling hydride transfer from various sources to a broad range of electrophiles.

Although the reactivity of aluminum of the type **1/2** complexes is largely ligand-centered, our findings reveal a striking contrast between carbon- and boron-based acceptors. Whereas trityl has a nearly ideal behavior and selectively abstracts hydride from the ligand, B­(C_6_F_5_)_3_ engages in competitive attack at both the hydropyridinate and the AlR′_2_ fragments. The latter leads to metal unsaturation, triggering fast hydride migration and leads to cationic byproducts (**2′**). This divergence underscores the influence of electric charge: the cationic nature of the carbonium reagent likely imposes an electrostatic barrier that protects the metal center from the attack, a constraint absent from the borane electrophile. Furthermore, we have identified a slow, thermodynamically driven H/R′ exchange between B and Al, which likely proceeds via reversible hydride transfer between [B­(H)­(C_6_F_5_)_3_]^−^ and cationic species of type **1**, rather than direct metathetical exchange. Introducing a benzyl substituent at C4 further complicates the exchange process, expanding the number of potential equilibria involving positions 3(5) and 4 of the pyridine ring.

The results reported herein open new avenues for both fundamental research and practical applications of 2,6-bis­(imino)­pyridine complexes. The demonstrated reversibility of hydride transfer suggests that **1**/**2**-type complexes could function as intermediate hydride carriers in bioinspired catalytic systems where the reducing equivalents are sourced from sustainable feedstocks, like water, alcohols, or hydrogen, before being transferred to electrophilic substrates in a subsequent step. Additionally, as demonstrated before, complexes of type **2** undergo clean dimerization on heating, providing a general and readily accessible pathway to ditopic N,N,N pincers, which can be transferred to a variety of transition and main-group metals. Both avenues are under investigation in our laboratory.

## Experimental Section

Most of the compounds included in this work are sensitive to oxygen and traces of moisture. Therefore, inert atmosphere Schlenk techniques or an N_2_-filled glovebox were routinely employed for experimental procedures. Solvents (dichloromethane, *N*-hexane, pentane, and diethyl ether) were rigorously degassed, dried, and distilled immediately prior to use. Instrumentation and procedures, NMR, EPR, ESI-MS, Elemental Analysis (EA), and X-ray diffraction studies data are included in the Supporting Information.

Li­[HBEt_3_] (1 M solution in THF), LiAlH_4_, AlMe_3_, [Ph_3_C]­[B­(C_6_F_5_)_4_] were purchased from Sigma-Aldrich and they were used as received. B­(C_6_F_5_)_3_ was acquired from TCI chemicals and it was sublimed prior employment. Ligand BIP (2,6-[2,6-^i^Pr_2_C_6_H_3_N = C­(Me)]_2_-C_5_H_3_N) was prepared from 2,6-diacetylpyridine and 2,6-[2,6-^i^Pr_2_C_6_H_3_NH_2_ following the usual condensation procedure in toluene under azeotropic water removal with a small amount of *p*-toluenesulfonic acid as a catalyst. [Al­(R)_2_(^DiPP^BIP)]­[X] (*R* = Me (**1a**); Et (**1b**); X = PF_6_; BAr^F^_4_),^28^ [Al­(Me)_2_(4-H-^Bn^BIP)] (**2c**)^21^ and the protonated tetra-arylborate salts [H­(^Bn^BIP)]­[BAr^F^_4_][Bibr cit18b] were prepared using our own reported methods.

### Synthesis of [Al­(Me)_2_(^Bn^BIP)]­[BAr^F^_4_] (**1c·BAr^F^_4_**)

A colorless solution of trimethyl aluminum (11 μL, 0.110 mmol) in 5 mL of dichloromethane at −30 °C was added slowly to a red solution of [H­(^Bn^BIP)]­[BArF_4_] (143.5 mg, 0.100 mmol) in 10 mL of dichloromethane at the same temperature. The mixture was magnetically stirred for 1 h at 25 °C, followed by the removal of solvent and volatiles under vacuum. The resulting solid residue was washed with hexane (3 × 5 mL), filtered, and dried, yielding a powdery dark red solid (135.7 mg, 90%) corresponding to complex **1c·BArF**_**4**_. This solid was then redissolved in 10 mL of dichloromethane, concentrated to 1 mL total volume, and 0.2 mL of hexane was added. After 24 h at −30 °C, dark red cubic crystals suitable for X-ray diffraction studies were formed.

^**1**^ **H NMR** (CD_2_Cl_2_, 25 °C, 400 MHz): δ −0.92 (s, 6H, Al*Me*_2_), 1.06 (d,^3^ *J*_HH_ = 6.9 Hz, 12H, CH*Me*Me), 1.21 (d,^3^ *J*_HH_ = 6.9 Hz, 12H, CHMe*Me*), 2.49 (s, 6H, *Me*(CN)), 2.51 (sept,^3^ *J*_HH_ = 6.9 Hz, 4H, *CH*MeMe), 4.43 (s, 2H, CH_2_ Py-Bn), 7.28–7.47 (*o,m,p*-CH_Ar_ Py-Bn), 7.31 (d,^3^ *J*_HH_ = 7.1 Hz, 4H, *m*-CH_N–Ar_), 7.38 (t,^3^ *J*_HH_ = 7.2 Hz, 2H, *p*-CH_N–Ar_), 7.55 (s, 4H, *p*-CH_Ar_ BAr^F^_4_), 7.72 (br s, 8H, *o*-CH_Ar_ BAr^F^_4_), 8.25 (s, 2H, 3,5-CH_Py_). ^**19**^ **F­{** ^**1**^ **H} NMR** (CD_2_Cl_2_, 25 °C, 376 MHz): δ −62.88 (s, 24F, BAr^*F*^_4_). ^**11**^ **B­{** ^**1**^ **H} NMR** (CD_2_Cl_2_, 25 °C, 128 MHz): δ −6.61 (s, 1B, *B*Ar^F^_4_). ^**13**^ **C­{** ^**1**^ **H} NMR** (CD_2_Cl_2_, 25 °C, 100 MHz): δ −8.27 (Al*Me*_2_), 18.92 (*Me*(CN)), 23.83 (CHMe*Me*), 24.79 (CH*Me*Me), 29.02 (*C*HMeMe), 42.35 (*C*H_2_ Py-Bn), 117.63 (*p-C*H_Ar_ BAr^F^_4_), 124.75 (q,^1^ *J*_CF_ = 272 Hz, *C*F_3_ BAr^F^_4_), 125.05 (*m*-CH_N–Ar_), 128.08 (3,5-*C*H_Py_), 128.45 (*p-C*H_Ar_ Py-Bn), 128.77 (*p-C*H_N–Ar_), 129.01 (q,^2^ *J*_CF_ = 35 Hz, *C*-CF_3_ BAr^F^_4_), 129.41 (*o-C*H_Ar_ Py-Bn), 129.85 (*m-C*H_Ar_ Py-Bn), 134.97 (*o-C*H_Ar_ BAr^F^_4_), 135.46 (*i-C*_Ar_ Py-Bn), 138.78 (*o-C*_N–Ar_), 139.56 (*i-C*_N–Ar_), 147.71 (2-*C*H_Py_), 162.11 (q,^1^ *J*_CB_ = 52 Hz, *i-C*_Ar_ BAr^F^_4_), 164.09 (4-*C*H_Py_), 169.91 (Me­(*C*N)). **Elemental analysis for** C_74_H_67_AlBF_24_N_3_ (found vs calculated, crystalline sample): C 59.65 (59.57), H 4.62 (4.53), N 2.92 (2.82).

### Synthesis of [Al­(Me)_2_(4-HBIP)] (**2a**)

#### Preparation A

In a Teflon J. Young-type screw-capped ampule, 315.8 mg (0.225 mmol) of complex **1a·BArF**_**4**_ was dissolved in 20 mL of CH_2_Cl_2_, and 225 μL of LiHBEt_3_ (0.225 mmol, 1 M in THF) was added, both solutions cooled at −30 °C. The mixture’s color changed immediately from yellow-brown to deep turquoise. The reaction mixture was stirred for 10 min at room temperature, resulting in a dark burgundy solution. Subsequently, solvents and volatiles were evaporated to isolate the crude product, which was extracted with hexane (4 × 5 mL), filtered through Celite, and dried under vacuum. A purple powdery solid was obtained, identified as complex [Al­(Me)_2_(4-HBIP)] (**2a**), yielding 100.8 mg (83%).

#### Preparation B

Compound **2a** was prepared by adding 336 μL of LiHBEt_3_ (0.336 mmol, 1 M in THF) to a yellow-brown solution of **1a·PF**_**6**_ (229.9 mg, 0.336 mmol) in 10 mL of CH_2_Cl_2_ at −30 °C. The same color changes observed in the previous procedure were noted. Following the evaporation of solvents and volatiles, the product was extracted with pentane (4 × 5 mL), filtered, and dried under vacuum. This process yielded 163.6 mg (89% yield) of complex **2a**, as a purple powdery solid.

#### Preparation C

A yellow-brown solution of **1a·BAr** ^**F**^_**4**_ (46.3 mg, 0.033 mmol) in 8 mL of Et_2_O was carefully added to a vigorously stirred suspension of LiAlH_4_ (1.37 mg, 0.036 mmol) in 5 mL of Et2O, both maintained at −30 °C. Instantly, the solution’s color changed from yellow-brown to deep turquoise. The reaction mixture was stirred for 45 min at room temperature, during which the solution turned dark burgundy. Subsequently, the solvent and volatiles were removed under reduced pressure, and the resultant crude product was extracted with hexane (2 × 5 mL), filtered, and dried. A purple solid was obtained, weighing 12.6 mg (71% yield), which, according to NMR spectra, corresponded to complex **2a**.

^**1**^ **H NMR** (C_6_D_6_, 25 °C, 400 MHz): δ −0.57 (s, 6H, Al*Me*_2_), 0.90 (d,^3^ *J*_HH_ = 6.9 Hz, 12H, CH*Me*Me), 1.36 (d,^3^ *J*_HH_ = 6.9 Hz, 12H, CHMe*Me*), 1.60 (s, 6H, *Me*(CN)), 2.97 (sept,^3^ *J*_HH_ = 6.9 Hz, 4H, *CH*MeMe), 3.54 (t,^3^ *J*_HH_ = 4.0 Hz, 2H, 4-CH_Py_), 4.93 (t,^3^ *J*_HH_ = 4.0 Hz, 2H, 3,5-CH_Py_), 7.07 (m, 6H, CH_N–Ar_)^.**13**^ **C­{** ^**1**^ **H} RMN** (C_6_D_6_, 25 °C, 100 MHz): δ −6.81 (Al*Me*_2_), 17.01 (*Me*(CN)), 24.34 (CHMe*Me*), 25.43 (CH*Me*Me), 27.21 (4-*C*H_Py_), 28.35 (*C*HMeMe), 103.35 (3,5-*C*H_Py_), 124.36 (*m*-*C*H_N–Ar_), 126.71 (*p*-*C*H_N–Ar_), 140.59 (2-*C*_Py_), 142.23 (*o*-*C*_N–Ar_), 143.33 (*i*-*C*_N–Ar_), 170.57 (Me­(*C*N)). **Elemental analysis for** C_35_H_50_AlN_3_ (found vs calculated, crystalline sample): C 77.70 (77.88), H 9.49 (9.34), N 8.03 (7.78).

### Synthesis of [Al­(Et)_2_(4-HBIP)] (**2b**)

#### Preparation A

The same experimental protocol utilized for the synthesis of complex **2a** was employed for the synthesis of complex **2b**. Specifically, 178 μL (0.178 mmol, 1 M in THF) of LiHBEt_3_ was added to a yellow-brown solution of **1b·BAr** ^**F**^_**4**_ (254.8 mg, 0.178 mmol) in 15 mL of Et2O at −30 °C. Similar color changes were observed as those during the preparation of **2a**. The product was extracted with hexane (4 × 5 mL), filtered, and dried, yielding a purple powdery solid weighing 80.9 mg (80% yield), corresponding to **2b** based on NMR analysis. Additionally, single purple crystals suitable for X-ray diffraction studies were obtained from a concentrated hexane solution (1 mL) at −30 °C after 48 h.

#### Preparation B

Compound **2b** was also prepared by adding 250 μL of LiHBEt_3_ (0.250 mmol, 1 M in THF) to a yellow-brown solution of **1b·PF**_**6**_ (178.3 mg, 0.250 mmol) in dichloromethane, following the same experimental protocol as described above. After extraction, filtering, and drying under vacuum, a purple solid was obtained, identified as complex **2b**, weighing 119.7 mg (84% yield).

^**1**^ **H NMR** (C_6_D_6_, 25 °C, 400 MHz): δ 0.15 (q,^3^ *J*_HH_ = 8.3 Hz, 4H, Al­(*CH*_2_CH_3_)_2_), 0.95 (d,^3^ *J*_HH_ = 7.1 Hz, 12H, CH*Me*Me), 1.02 (t,^3^ *J*_HH_ = 7.1 Hz 6H, Al­(CH_2_ *CH*_3_)_2_), 1.41 (d,^3^ *J*_HH_ = 6.9 Hz, 12H, CHMe*Me*), 1.63 (s, 6H, *Me*(CN)), 3.02 (sept,^3^ *J*_HH_ = 6.8 Hz, 4H, *CH*MeMe), 3.55 (t,^3^ *J*_HH_ = 3.9 Hz, 2H, 4-CH_Py_), 4.92 (t,^3^ *J*_HH_ = 4.0 Hz, 2H, 3,5-CH_Py_), 7.09 (m, 6H, CH_N–Ar_). ^**13**^ **C­{** ^**1**^ **H} RMN** (C_6_D_6_, 25 °C, 100 MHz): δ 0.84 (Al­(CH_2_ *C*H_3_)_2_), 10.24 (Al­(*C*H_2_CH_3_)_2_), 16.80 (*Me*(CN)), 24.13 (CHMe*Me*), 25.30 (CH*Me*Me), 27.20 (4-*C*H_Py_), 28.20 (*C*HMeMe), 103.17 (3,5-*C*H_Py_), 124.27 (*m*-*C*H_N–Ar_), 126.67 (*p*-*C*H_N–Ar_), 140.47 (2-*C*_Py_), 143.08 (o-*C*_N–Ar_), 143.59 (*i*-*C*_N–Ar_), 171.21 (Me­(*C*N)). **Elemental analysis for C**_**37**_ **H**_**54**_ **AlN**_**3**_ (found vs. calculated, crystallized sample): C 77.90 (78.26), H 9.60 (9.59), N 7.61 (7.40).

### Reaction of **2a** with [Ph_3_C]­[B­(C_6_F_5_)_4_]. Synthesis of **1a·B­(C_6_F_5_)_4_**

#### NMR Tube Scale Reaction

In an NMR tube scale reaction, a cold yellow solution of [Ph_3_C]­[B­(C_6_F_5_)_4_] (20.7 mg, 0.022 mmol) in 0.4 mL of dichloromethane-*d*_2_ was added to a purple solution of compound **2a** (12.1 mg, 0.022 mmol) in the same volume of solvent at −30 °C. An immediate color change to dark red was observed. After 5 min, NMR analysis indicated the absence of signals from both reagents, revealing only signals corresponding to compound **1a·B­(C**_**6**_ **F**_**5**_)_**4**_ and Ph_3_CH.

#### Preparative Scale Reaction

A yellow solution of [Ph_3_C]­[B­(C_6_F_5_)_4_] (111.7 mg, 0.121 mmol) in 10 mL of dichloromethane was slowly added to another 10 mL dichloromethane solution of 2a (65.3 mg, 0.121 mmol), both cooled at −30 °C. This addition resulted in an immediate color change to dark red. The mixture was stirred vigorously and allowed to gradually reach room temperature. After 15 min of stirring, the solution was evaporated to dryness, yielding a dark red solid residue. NMR spectra confirmed the presence of complex **1a·B­(C**_**6**_ **F**_**5**_)_**4**_ and Ph_3_CH. Subsequently, the solid was washed with pentane (4 × 8 mL) and dried under reduced pressure, resulting in 127.4 mg (85% yield) of a pink microcrystalline solid identified as compound **1a·B­(C**_**6**_ **F**_**5**_)_**4**_.

^**1**^ **H NMR** (CD_2_Cl_2_, 25 °C, 400 MHz): δ −0.90 (s, 6H, Al*Me*_2_), 1.08 (d,^3^ *J*_HH_ = 6.8 Hz, 12H, CH*Me*Me), 1.23 (d,^3^ *J*_HH_ = 7.0 Hz, 12H, CHMe*Me*), 2.53 (sept,^3^ *J*_HH_ = 7.0 Hz, 4H, *CH*MeMe), 2.57 (s, 6H, *Me*(CN)), 7.32 (d,^3^ *J*_HH_ = 7.3 Hz, 4H, *m*-CH_N–Ar_), 7.40 (t,^3^ *J*_HH_ = 7.4 Hz, 2H, *p*-CH_N–Ar_), 8.50 (d,^3^ *J*_HH_ = 7.1 Hz, 2H, 3,5-CH_Py_), 8.76 (t,^3^ *J*_HH_ = 7.0 Hz, 1H, 4-CH_Py_). ^**19**^ **F­{** ^**1**^ **H} NMR** (CD_2_Cl_2_, 25 °C, 376 MHz): δ −132.99 (br d, 2F, (*o*-*F*) [B­(C_6_F_5_)_4_]^−^), −163.54 (t,^3^ *J*_FF_ = 19 Hz, 1F, (*p-F*) [B­(C_6_F_5_)_4_]^−^) −167.47 (t,^3^ *J*_FF_ = 19 Hz, 2F, (*m-F*) [B­(C_6_F_5_)_4_]^−^). ^**11**^ **B­{** ^**1**^ **H} NMR** (CD_2_Cl_2_, 25 °C, 128 MHz): δ −16.65 (s, 1B, [*B*(C_6_F_5_)_4_]^−^). ^**13**^ **C­{** ^**1**^ **H} NMR** (CD_2_Cl_2_, 25 °C, 100 MHz): δ −7.83 (Al*Me*_2_), 19.16 (*Me*(CN)), 24.10 (CHMe*Me*), 24.97 (CH*Me*Me), 29.32 (*C*HMeMe), 125.29 (*m*-CH_N–Ar_), 128.40 (3,5-*C*H_Py_), 129.04 (*p-C*H_N–Ar_), 136.72 (dm, ^1^ *J*_CF_ = 245 Hz, B­(*C*_6_F_5_)_4_]^−^), 138.64 (dm, ^1^ *J*_CF_ = 242 Hz, B­(*C*_6_F_5_)_4_]^−^), 139.03 (*o-C*_N–Ar_), 139.92 (*i-C*_N–Ar_), 147.25 (4-*C*H_Py_), 147.95 (2-*C*H_Py_), 148.50 (dm, ^1^ *J*_CF_ = 245 Hz, B­(*C*_6_F_5_)_4_]^−^), 170.29 (Me­(*C*N)). **Elemental analysis for** C_59_H_49_AlBF_20_N_3_ (found vs calculated, crystalline sample): C 58.32 (58.19), H 3.94 (4.06), N 3.51 (3.45).

### Reaction of **2b** with [Ph_3_C]­[B­(C_6_F_5_)_4_]. Synthesis of **1b·B­(C_6_F_5_)_4_**

The synthesis of **1b·B­(C**_**6**_ **F**_**5**_)_**4**_ was conducted following the same experimental protocol described above for **1a·B­(C**_**6**_ **F**_**5**_)_**4**_. In this instance, 51.9 mg (0.091 mmol) of compound 2b and 84.3 mg (0.091 mmol) of [Ph_3_C]­[B­(C_6_F_5_)_4_] were reacted at −30 °C. The reaction mixture was stirred for 15 min before the solvent was removed, the solid residue was washed with pentane (3 × 8 mL) and dried under vacuum, yielding a pink solid (92.2 mg; 81% yield) whose NMR spectra corresponded exclusively to the ionic complex **1b·B­(C**_**6**_ **F**_**5**_)_**4**_. Subsequently, the product was redissolved in 10 mL of dichloromethane, filtered, concentrated to one-third of its original volume, and stored at −30 °C. After 72 h, red-orange plate crystals suitable for X-ray diffraction studies were obtained.

^**1**^ **H NMR** (CD_2_Cl_2_, 25 °C, 400 MHz): δ −0.05 (q, *J*_HH_ = 7.7 Hz, 4H, Al­(*CH*_2_CH_3_)_2_), 0.10 (t,^3^ *J*_HH_ = 7.2 Hz 6H, Al­(CH_2_ *CH*_3_)_2_), 1.09 (d,^3^ *J*_HH_ = 6.8 Hz, 12H, CH*Me*Me), 1.27 (d,^3^ *J*_HH_ = 6.9 Hz, 12H, CHMe*Me*), 2.50 (sept,^3^ *J*_HH_ = 7.2 Hz, 4H, *CH*MeMe), 2.58 (s, 6H, *Me*(CN)), 7.32 (d,^3^ *J*_HH_ = 7.0 Hz, 4H, *m*-CH_N–Ar_), 7.41 (t,^3^ *J*_HH_ = 7.2 Hz, 2H, *p*-CH_N–Ar_), 8.52 (d,^3^ *J*_HH_ = 7.1 Hz, 2H, 3,5-CH_Py_), 8.71 (t,^3^ *J*_HH_ = 7.1 Hz, 1H, 4-CH_Py_). ^**19**^ **F­{** ^**1**^ **H} NMR** (CD_2_Cl_2_, 25 °C, 376 MHz): δ −133.03 (br d, 2F, (*o*-*F*) [B­(C_6_F_5_)_4_]^−^), −163.61 (t,^3^ *J*_FF_ = 19 Hz, 1F, (*p-F*) [B­(C_6_F_5_)_4_]^−^) −167.54 (t,^3^ *J*_FF_ = 19 Hz, 2F, (*m-F*) [B­(C_6_F_5_)_4_]^−^). ^**11**^ **B­{** ^**1**^ **H} NMR** (CD_2_Cl_2_, 25 °C, 128 MHz): δ −16.66 (s, 1B, [*B*(C_6_F_5_)_4_]^−^). ^**13**^ **C­{** ^**1**^ **H} NMR** (CD_2_Cl_2_, 25 °C, 100 MHz): δ −1.28 (Al­(CH_2_ *C*H_3_)_2_), 8.08 (Al­(*C*H_2_CH_3_)_2_), 19.15 (*Me*(CN)), 23.92 (CHMe*Me*), 25.08 (CH*Me*Me), 29.30 (*C*HMeMe), 125.30 (*m*-CH_N–Ar_), 128.04 (3,5-*C*H_Py_), 129.11 (*p-C*H_N–Ar_), 135.69 (dm, ^1^ *J*_CF_ = 247 Hz, B­(*C*_6_F_5_)_4_]^−^), 138.83 (dm, ^1^ *J*_CF_ = 244 Hz, B­(*C*_6_F_5_)_4_]^−^), 139.59 (*o-C*_N–Ar_), 139.90 (*i-C*_N–Ar_), 146.85 (4-*C*H_Py_), 147.35 (2-*C*H_Py_), 148.54 (dm, ^1^ *J*_CF_ = 242 Hz, B­(*C*_6_F_5_)_4_]^−^), 170.55 (Me­(*C*N)). **Elemental analysis for** C_61_H_53_AlBF_20_N_3_ (found vs calculated, crystalline sample): C 58.88 (58.81), H 4.51 (4.29), N 3.59 (3.37).

### Reaction of **2a** with B­(C_6_F_5_)_3_. Formation of [Al­(Me)_2_(BIP)]^+^ (**1a^+^**) and [Al­(H)­(Me)­(BIP)] [MeB­(C_6_F_5_)_3_]^**+**^ (**1′a^+^**) Paired with [HB­(C_6_F_5_)_3_]^−^ and [MeB­(C_6_F_5_)_3_]^−^

#### NMR Tube Scale Reaction

A colorless solution of B­(C_6_F_5_)_3_ (12.0 mg, 0.023 mmol) in 0.3 mL of dichloromethane-*d*_2_ at −30 °C was carefully added to a purple solution of compound 2a (12.7 mg, 0.023 mmol) in 0.3 mL of CD_2_Cl_2_, also at −30 °C. Immediately, the solution changed from purple to magenta and was analyzed by NMR. After 5 min at room temperature, the ^1^H NMR spectrum revealed signals corresponding to **1a** ^**+**^ (**1′a** ^**+**^ signals could not be properly appreciated in this solvent) counterbalanced by **[HB­(C**_**6**_ **F**_**5**_)_**3**_]^**–**^ **/[MeB­(C**_**6**_ **F**_**5**_)_**3**_]^**–**^ in a relative ratio of 3:1, respectively. The relative ratio of both species were confirmed by ^11^B and ^19^F-NMR. Subsequently, the solution was dried and redissolved in C_6_D_6_. The NMR spectra (^1^H, ^11^B, and ^19^F) exhibited two sets of resonances attributable to the products mixture **1a/** **1′a·HB­(C**_**6**_ **F**_**5**_)_**3**_ **/MeB­(C**_**6**_ **F**_**5**_)_**3**_ in a relative ratio of 1:3.

#### VT-NMR Tube Scale Reaction Monitoring

A colorless solution of B­(C_6_F_5_)_3_ (18.4 mg, 0.035 mmol) in 0.3 mL of CD_2_Cl_2_ at −50 °C was added via pipet into a vial containing a purple solution of compound **2a** (19.4 mg, 0.035 mmol) in 0.3 mL of CD_2_Cl_2_, also at −50 °C. The resultant mixture was subsequently analyzed by NMR (^1^H, ^11^B, and ^19^F) at −40 °C, revealing signals corresponding to compounds **1a** ^**+**^ (and **1′a** ^**+**^, though this could not be identified), and the anions **[HB­(C**_**6**_ **F**_**5**_)_**3**_]^−^ and **[MeB­(C**_**6**_ **F**_**5**_)_**3**_]^−^ in a relative ratio of approximately 3:1. The reaction mixture was then slowly warmed from −40 to 25 °C over a period of 75 min, with NMR recordings taken at 10-degree intervals. The composition of the sample remained virtually unchanged. The solution was kept at room temperature for 29 h, during which the relative ratio of the mixture **1a**/**1′a**·**HB­(C**_**6**_ **F**_**5**_)_**3**_/**MeB­(C**_**6**_ **F**_**5**_)_**3**_ evolved from approximately 3:1 to 1:1. Subsequently, the solution was heated to 55 °C over a period of 1 week, with NMR spectra recorded at 24-h intervals. This provided a final relative ratio for the mixture **1a**/**1′a·HB­(C**_**6**_ **F**_**5**_)_**3**_/**MeB­(C**_**6**_ **F**_**5**_)_**3**_ of 1:3, marking the conclusion of the experiment.

#### Preparative Scale Reaction

In a scintillation vial, 62.3 mg (0.115 mmol) of complex **2a** was dissolved in 7 mL of dichloromethane and stored at −30 °C. B­(C_6_F_5_)_3_ (59.1 mg, 0.115 mmol) was also dissolved in cold CH_2_Cl_2_ and stored at −30 °C in another vial. The two solutions were mixed by adding the borane solution dropwise to the **2a** complex solution, resulting in an immediate color change from purple to magenta. After stirring for 10 min at room temperature, the solution was evaporated to dryness. The crude reaction product was then washed with pentane (3 × 8 mL), filtered, and dried again. Finally, an analytically pure pink microcrystalline solid (105.2 mg, 87% yield) was obtained, and NMR analysis confirmed the formation of the mixture **1a**/**1′a·HB­(C**_**6**_ **F**_**5**_)_**3**_/**MeB­(C**_**6**_ **F**_**5**_)_**3**_ in a relative ratio of approximately 3:1.

^**1**^ **H NMR of 1a·HB­(C**_**6**_ **F**_**5**_)_**3**_/**MeB­(C**_**6**_ **F**_**5**_)_**3**_ (CD_2_Cl_2_, 25 °C, 400 MHz): δ −0.91 (s, 6H, Al*Me*_2_), 1.07 (d,^3^ *J*_HH_ = 6.8 Hz, 12H, CH*Me*Me), 1.22 (d,^3^ *J*_HH_ = 7.1 Hz, 12H, CHMe*Me*), 2.55 (sept,^3^ *J*_HH_ = 7.2 Hz, 4H, *CH*MeMe), 2.57 (s, 6H, *Me*(CN)), 7.31 (d,^3^ *J*_HH_ = 7.2 Hz, 4H, *m*-CH_N–Ar_), 7.39 (t,^3^ *J*_HH_ = 7.1 Hz, 2H, *p*-CH_N–Ar_), 8.52 (d,^3^ *J*_HH_ = 7.1 Hz, 2H, 3,5-CH_Py_), 8.73 (t,^3^ *J*_HH_ = 7.0 Hz 1H, 4-CH_Py_). Signals of **1′a** ^**+**^ obscured by **1a** ^**+**^; the **[** *Me* **B­(C**_**6**_ **F**_**5**_)_**3**_]^**–**^ was observed at 0.42 ppm (br s). ^**19**^ **F­{** ^**1**^ **H}** (CD_2_Cl_2_, 25 °C, 376 MHz): Mixture of anions **[HB­(C**_**6**_ **F**_**5**_)_**3**_]^**–**^: δ −133.88 (d,^3^ *J*_FF_ = 19 Hz, 2F, (*o*-*F*), −164.40 (t,^3^ *J*_FF_ = 19 Hz, 1F, (*p-F*), −167.41 (t,^3^ *J*_FF_ = 19 Hz, 2F, (*m-F*). **[MeB­(C**_**6**_ **F**_**5**_)_**3**_]^**–**^: δ −133.07 (d,^3^ *J*_FF_ = 19 Hz, 2F, (*o*-*F*)), −165.10 (t,^3^ *J*_FF_ = 19 Hz, 1F, (*p-F*), −167.78 (t,^3^ *J*_FF_ = 19 Hz, 2F, (*m-F*). ^**11**^ **B NMR** (CD_2_Cl_2_, 25 °C, 128 MHz): δ −25.37 (d,^1^ *J*_BH_ = 93 Hz, **[H*B*(C_6_F_5_)_3_]^–^**). −14.96 (s, **[Me*B*(C_6_F_5_)_3_]^–^**).

^**1**^ **H NMR of 1** *a* **/1a′·HB­(C**_**6**_ **F**_**5**_)_**3**_/**MeB­(C**_**6**_ **F**_**5**_)_**3**_ (C_6_D_6_, 25 °C, 400 MHz): **Cation 1a** ^**+**^ δ −0.76 (s, 6H, Al*Me*_2_), 0.91 (overlapping doublets,^3^ *J*_HH_ = 6.7 Hz, 12H, CH*Me*Me), 1.15 (d,^3^ *J*_HH_ = 6.9 Hz, 12H, CHMe*Me*), 1.94 (s, 6H, *Me*(CN)), 2.46 (sept,^3^ *J*_HH_ = 7.1 Hz, 4H, *CH*MeMe), 6.97 (d,^3^ *J*_HH_ = 7.0 Hz, 4H, *m*-CH_N–Ar_), 7.05 (t,^3^ *J*_HH_ = 7.1 Hz, 2H, *p*-CH_N–Ar_), 7.56 (d,^3^ *J*_HH_ = 7.0 Hz, 2H, 3,5-CH_Py_), 7.85 (t,^3^ *J*_HH_ = 7.1 Hz 1H, 4-CH_Py_). **Cation 1′a** ^**+**^ (C_6_D_6_, 25 °C, 400 MHz): δ −0.82 (s, 3H, Al*Me*(H)), 0.96 (overlapped doublet,^3^ *J*_HH_ = 7.0 Hz, 6H, CH*Me*Me), 1.30 (overlapped doublet,^3^ *J*_HH_ = 6.9 Hz, 12H, CH*Me*Me), 1.91 (s, 6H, *Me*(CN)), 2.39 (sept,^3^ *J*_HH_ = 6.8 Hz, 2H, *CH*MeMe), 2.85 (sept,^3^ *J*_HH_ = 6.9 Hz, 2H, *CH*MeMe), 7.04 (d,^3^ *J*_HH_ = 7.0 Hz, 4H, *m*-CH_N–Ar_), 7.44 (d,^3^ *J*_HH_ = 7.1 Hz, 2H, 3,5-CH_Py_), 7.78 (t,^3^ *J*_HH_ = 7.0 Hz, 1H, 4-CH_Py_). *The signals corresponding to one CHMe* **Me**, *p*-CH_N–Ar_ and Al-*H could not be found.* **[** *Me* **B­(C**_**6**_ **F**_**5**_)_**3**_]^**–**^ was observed at 0.42 (br s)^.**19**^ **F­{** ^**1**^ **H} NMR** (C_6_D_6_, 25 °C, 376 MHz): Mixture of anions, **[HB­(C**_**6**_ **F**_**5**_)_**3**_]^**–**^: δ −133.74 (d,^3^ *J*_FF_ = 19 Hz, 2F, (*o*-*F*)), −164.38 (t,^3^ *J*_FF_ = 19 Hz, 1F, (*p-F*)) −167.47 (t,^3^ *J*_FF_ = 19 Hz, 2F, (*m-F*)). [**MeB­(C**_**6**_ **F**_**5**_)_**3**_]^**–**^: δ −132.84 (d,^3^ *J*_FF_ = 19 Hz, 2F, (*o*-*F*)), −165.18 (t,^3^ *J*_FF_ = 19 Hz, 1F, (*p-F*) −167.88 (t,^3^ *J*_FF_ = 19 Hz, 2F, (*m-F*)). ^**11**^ **B NMR** (C_6_D_6_, 25 °C, 128 MHz): δ −24.78 (br d,^1^ *J*_BH_ = 93 Hz [HB­(C_6_F_5_)_3_]^**–**^), −14.36 (s, [Me*B*(C_6_F_5_)_3_]^**–**^).

**ESI-MS (CH**_**2**_ **Cl**_**2**_): 513.0 [HB­(C_6_F_5_)_3_]^−^; 527.1 [MeB­(C_6_F_5_)_3_]^−^; 538.5 [^DiPP^BIPAlMe_2_]^+^. **Elemental analysis for** C_53_H_50_AlBF_15_N_3_ (found vs calculated): C 60.39 (60.52), H 4.72 (4.79), N 3.89 (4.00).

### Reaction of **2b** with B­(C_6_F_5_)_3_. Formation of [Al­(Et)_2_(BIP)]^+^ (**1b^+^**) and [Al­(H)­(Et)­(BIP)] (1′b^**+**^) Paired with [HB­(C_6_F_5_)_3_]^−^ and [EtB­(C_6_F_5_)_3_]^−^

#### NMR Tube Scale Reaction

In a scintillation vial, 13.6 mg (0.027 mmol) of B­(C_6_F_5_)_3_ were dissolved in 0.3 mL of CD_2_Cl_2_ and cooled to −30 °C before being carefully added to an equimolar purple solution of **2b** (15.1 mg, 0.027 mmol, in 0.3 mL of CD_2_Cl_2_), also at −30 °C. The solution immediately changed from purple to dark red. After 10 min at room temperature, the resultant solution was analyzed by NMR (^1^H, ^19^F, ^11^B), revealing the disappearance of signals from the reagents and the emergence of a new set of resonances corresponding to **1**b**/1**′**b**·**HB­(C**_**6**_ **F**_**5**_)_**3**_ **/EtB­(C**_**6**_ **F**_**5**_)_**3**_ in a relative ratio of 8:1.

^**1**^ **H NMR** (CD_2_Cl_2_, 25 °C, 400 MHz): **1b** ^**+**^, δ −0.06 (q,^3^ *J*_HH_ = 7.5 Hz, 4H, Al­(*CH*_2_CH_3_)_2_), 0.10 (t,^3^ *J*_HH_ = 7.6 Hz, 6H, Al­(CH_2_ *CH*_3_)_2_), 1.08 (d,^3^ *J*_HH_ = 6.9 Hz, 12H, CH*Me*Me), 1.26 (d,^3^ *J*_HH_ = 6.7 Hz, 12H, CHMe*Me*), 2.51 (sept,^3^ *J*_HH_ = 6.8 Hz, 4H, *CH*MeMe), 2.58 (s, 6H, *Me*(CN)), 7.32 (d,^3^ *J*_HH_ = 7.5 Hz, 4H, *m*-CH_N–Ar_), 7.40 (t,^3^ *J*_HH_ = 7.1 Hz, 2H, *p*-CH_N–Ar_), 8.55 (d,^3^ *J*_HH_ = 7.9 Hz, 2H, 3,5-CH_Py_), 8.69 (t,^3^ *J*_HH_ = 8.0 Hz, 1H, 4-CH_Py_). **[EtB­(C**_**6**_ **F**_**5**_)_**3**_]^**–**^, δ 0.54 (br t, 3H, [(*CH*_3_CH_2_)­B­(C_6_F_5_)_3_]^**–**^). *Signals of* **1**′**b** ^***+***^ *and −CH*_2_ *- of [(CH*_3_ ***CH***_***2***_ *)­B­(C*_6_ *F*_5_)_3_] ^**–**^ *) could not be assigned.* ^**19**^ **F­{** ^**1**^ **H} NMR** (CD_2_Cl_2_, 25 °C, 376 MHz): **[HB­(C**_**6**_ **F**_**5**_)_**3**_] ^**–**^, δ −133.87 (d,^3^ *J*_FF_ = 20 Hz, 2F, (*o*-*F*)), −164.41 (t,^3^ *J*_FF_ = 19 Hz, 1F, (*p-F*)) −167.42 (t,^3^ *J*_FF_ = 19 Hz, 2F, (*m-F*)). **[EtB­(C**_**6**_ **F**_**5**_)_**3**_]^**–**^, δ −132.52 (d,^3^ *J*_FF_ = 20 Hz, 2F, (*o*-*F*)), −165.12 (t,^3^ *J*_FF_ = 19 Hz, 1F, (*p-F*), −167.79 (t,^3^ *J*_FF_ = 19 Hz, 2F, (*m-F*)). ^**11**^ **B NMR** (CD_2_Cl_2_, 25 °C, 128 MHz): δ −25.42 (d,^1^ *J*_BH_ = 90 Hz, [H*B*(C_6_F_5_)_3_]^**–**^), −12.63 (s, [Et*B*(C_6_F_5_)_3_]^**–**^).

### Reaction of **1c·BAr^F^_4_** with LiHBEt_3._ Formation of [Al­(Me)_2_(^Bn^BIP)] (**2c**) and [Al­(Me)_2_(^Bn^BIP)] (**3c**).

In a nitrogen-filled glovebox, a dark red solution of **1c·BAr** ^**F**^_**4**_ (77.2 mg, 0.052 mmol) in 8 mL of dichloromethane was prepared in a 30 mL vial. The solution was stored at −30 °C, and after 30 min, 52 μL of LiHBEt_3_ (0.052 mmol, 1 M in THF) were added slowly. The resultant mixture instantly turned deep royal blue. The reaction mixture was then stirred for 10 min at room temperature before the solvent was removed to dryness, yielding an oily crude product. This was extracted with pentane (2 × 5 mL), filtered, and dried again, affording 32.6 mg (76% yield) of a microcrystalline dark blue solid. The ^1^H NMR analysis showed two sets of signals corresponding to **2c** ^21^ and **3c** in a relative ratio of 1:5.

^**1**^ **H NMR** (C_6_D_6_, 25 °C, 400 MHz): **2c,** δ −0.61 (s, 3H, Al*Me*), −0.62 (s, 3H, Al*Me*), 0.95 (d,^3^ *J*_HH_ = 6.7 Hz, 6H, CH*Me*Me), 1.65 (s, 6H, *Me*(CN)), 2.84 (d,^3^ *J*_HH_ = 6.9 Hz, 2H, CH_2_ Py–Bn), 2.96 (m, ^3^ *J*_HH_ = 6.7 Hz, 4H, *CH*MeMe), 4.06 (m, 1H, 4-CH_Py_), 5.07 (d,^3^ *J*_HH_ = 4.0 Hz, 2H, 3,5-CH_Py_), 7.06–7.24 (m, 11H, CH_Ar_). *One CH*Me**Me** *signals could not be located.* **3c**, δ −0.52 (s, 6H, Al*Me*_2_), 1.00 (d,^3^ *J*_HH_ = 6.9 Hz, 6H, CH*Me*Me), 1.03 (d,^3^ *J*_HH_ = 7.0 Hz, 6H, CH*Me*Me), 1.32 (overlapped doublet,^3^ *J*_HH_ = 7.3 Hz, 12H, CH*Me*Me), 1.62 (s, 3H, *Me*(CN)), 3.07 (sept,^3^ *J*_HH_ = 7.1 Hz, 2H, *CH*MeMe), 3.14 (s, 2H, CH_2_, Py-Bn), 3.17 (sept,^3^ *J*_HH_ = 6.9 Hz, 2H, *CH*MeMe), 3.35 (s, 2H, 3-CH_2 Py_), 6.01 (s, 1H, 5-CH_Py_), 7.06–7.24 (m, 11H, CH_Ar_). *The second* **Me** *(CN) signal could not be located.* ^**13**^ **C­{** ^**1**^ **H} NMR** (C_6_D_6_, 25 °C, 300 MHz): **2c,** δ −7.14 (Al*Me*), −6.86 (Al*Me*), 17.11 (*Me*(CN)), 24.31­(CH*Me*Me), 25.47 (CHMe*Me*), 28.36 (*CH*MeMe), 39.51 (4-*C*H_Py_), 47.86 (*C*H_2_, Py–Bn), 106.59 (3,5-*C*H_Py_), 124.39 (*p-*CH_N–Ar_), 126.44 (*m*-CH_N–Ar_), 126.78 (*p*-CH_Ar_, Py–Bn), 128.69 (*o*-CH_Ar_, Py–Bn), 129.78 (*m*-CH_Ar_, Py–Bn), 139.31 (*i*-C_Ar_, Py–Bn), 140.57 (2-*C*_Py_), 142.39 (*i*-C_N–Ar_), 170.97 (Me­(*C*N)). *The o-C*_*N–Ar*_ *could not be located.* **3c,** δ −5.17 (Al*Me*_2_), 16.45 (*Me*(CN)), 17.80 (*Me*(CN)), 24.56, 26.66, 24.90, 24.93 (CH*Me*Me), 28.52 (*CH*MeMe), 28.57 (*CH*MeMe), 31.14 (3-*C*H_2 Py_), 44.51 (*C*H_2_, Py–Bn), 117.13 (5-*C*H_Py_), 121.82 (4-*C*H_Py_), 123.94 (*p-*CH_N–Ar_), 124.03 (*p-*CH_N–Ar_), 126.16 (*m*-CH_N–Ar_), 126.34 (*m*-CH_N–Ar_), 126.98 (*p*-CH_Ar_, Py–Bn), 128.98 (*o*-CH_Ar_, Py–Bn), 129.44 (*m*-CH_Ar_, Py–Bn), 137.99 (*i*-C_Ar_, Py–Bn), 140.57 (2-*C*_Py_), 142.12 (*o*-C_N–Ar_), 143.04 (*o*-C_N–Ar_), 143.37 (*i*-C_N–Ar_), 143.40 (*i*-C_N–Ar_), 162.56 (Me­(*C*N)), 166.17 (Me­(*C*N)).

### Reaction of the Mixture of **2c** and **3c** with [Ph_3_C]­[B­(C_6_F_5_)_4_]. Formation of [Al­(Me)_2_(^Bn^BIP)]­[B­(C_6_F_5_)_4_] (**1c·B­(C_6_F_5_)_4_**)

#### NMR Tube Scale Reaction

In two scintillation vials, solutions of [Ph_3_C]­[B­(C_6_F_5_)_4_] (16.0 mg, 0.017 mmol) and a mixture of **2c** and **3c** (10.9 mg, 0.017 mmol) were prepared, both dissolved in 0.3 mL of CD_2_Cl_2_, and stored at −30 °C prior to mixing. The yellow solution containing [Ph_3_C]­[B­(C_6_F_5_)_4_] was then added to the mixture of **2c** and **3c**, resulting in an immediate color change from dark blue to dark red. The content of the vial was subsequently transferred to an NMR J-Young tube. The NMR spectra of the reaction mixture showed only one set of resonances assigned to **1c·B­(C**_**6**_ **F**_**5**_)_**4**_, and Ph_3_CH (the latter omitted from the listing).

^**1**^ **H NMR** (CD_2_Cl_2_, 25 °C, 400 MHz): δ −0.93 (s, 6H, Al*Me*_2_), 1.06 (d,^3^ *J*_HH_ = 7.0 Hz, 12H, CH*Me*Me), 1.21 (d,^3^ *J*_HH_ = 7.1 Hz, 12H, CHMe*Me*), 2.49 (s, 6H, *Me*(CN)), 2.50 (sept,^3^ *J*_HH_ = 6.9 Hz, 4H, *CH*MeMe), 4.41 (s, 2H, CH_2_ Py-Bn), 7.25–7.40 (m, 6H, *m,p*-CH_N–Ar_), 7.35–7.49 (m, 5H, *o,m,p*-CH_N–Ar_ Py-Bn), 8.25 (s, 2H, 3,5-CH_Py_). ^**19**^ **F­{** ^**1**^ **H} NMR** (CD_2_Cl_2_, 25 °C, 376 MHz): δ −133.02 (br d, 2F, (*o*-*F*) [B­(C_6_F_5_)_4_]^−^), −163.68 (t,^3^ *J*_FF_ = 19 Hz, 1F, (*p-F*) [B­(C_6_F_5_)_4_]^−^) −167.56 (t,^3^ *J*_FF_ = 19 Hz, 2F, (*m-F*) [B­(C_6_F_5_)_4_]^−^). ^**11**^ **B­{** ^**1**^ **H} NMR** (CD_2_Cl_2_, 25 °C, 128 MHz): δ −16.66 (s, [*B*(C_6_F_5_)_4_]^−^). ^**13**^ **C­{** ^**1**^ **H} NMR** (CD_2_Cl_2_, 25 °C, 100 MHz): δ −8.05 (Al*Me*_2_), 19.14 (*Me*(CN)), 24.08 (CHMe*Me*), 25.03 (CH*Me*Me), 29.26 (*C*HMeMe), 42.51 (*C*H_2_ Py-Bn), 125.27 (*m*-CH_N–Ar_), 128.31 (3,5-*C*H_Py_), 128.65 (*p-C*H_N–Ar_), 128.98 (*p-C*H_Ar_ Py-Bn), 129.66 (*o-C*H_Ar_ Py-Bn), 130.07 (*m-C*H_Ar_ Py-Bn), 135.83 (*o-C*_N–Ar_), 136.71 (dm, ^1^ *J*_CF_ = 246 Hz, B­(*C*_6_F_5_)_4_]^−^), 138.61 (dm, ^1^ *J*_CF_ = 246 Hz, B­(*C*_6_F_5_)_4_]^−^), 139.05 (*i-C*_Ar_ Py-Bn), 139.85 (*i-C*_N–Ar_), 147.95 (2-*C*H_Py_), 148.55 (dm, ^1^ *J*_CF_ = 241 Hz, B­(*C*_6_F_5_)_4_]^−^), 164.36 (4-*C*H_Py_), 170.22 (Me­(*C*N)).

### Synthesis of **1c·B­(C_6_F_5_)_4_**

A cold yellow dichloromethane solution (8 mL) of [Ph_3_C]­[B­(C_6_F_5_)_4_] (103.1 mg, 0.112 mmol) was added via syringe to a purple solution of **2c** (70.3 mg, 0.112 mmol) in the same volume of solvent at −30 °C, resulting in an instantaneous color change of the mixture to dark red. The reaction mixture was stirred for 10 min at room temperature before solvents and volatiles were evaporated, yielding a dark red oily solid residue. This residue was washed with pentane (3 × 8 mL), filtered, and dried again, resulting in 131.8 mg (90% yield) of a pink microcrystalline solid. NMR spectra displayed only the signals corresponding to the compound **1c·B­(C**_**6**_ **F**_**5**_)_**4**_. **Elemental analysis for** C_66_H_55_AlBF_20_N_3_ (found vs calculated, crystalline sample): C 60.68 (60.61), H 4.44 (4.24), N 3.31 (3.21).

### Reaction of the Mixture of **2c** and **3c** with B­(C_6_F_5_)_3_

B­(C_6_F_5_)_3_ (12.5 mg, 0.024 mmol) diluted in 0.4 mL of CD_2_Cl_2_ at −30 °C was added dropwise to a deep royal blue solution containing 15.4 mg (0.024 mmol) of the mixture of **2c** and **3c** in 0.4 mL of CD_2_Cl_2_ at −30 °C. The resultant solution turned purple immediately and was transferred to an NMR J-Young tube for analysis. The ^1^H spectrum revealed the presence of products **1c** ^**+**^ and **1′c** ^**+**^ in a relative ratio of 1:1. The ^11^B and ^19^F spectra exhibited signals from the corresponding counteranions **[HB­(C**_**6**_ **F**_**5**_)_**3**_]^**–**^ and **[MeB­(C**_**6**_ **F**_**5**_)_**3**_]^**–**^ in a relative ratio of approximately 1:3. After 30 min, EPR analysis confirmed the presence of paramagnetic species (see SI). NMR analysis after 24 h at room temperature showed the same products (**1c** ^**+**^ and **1′c** ^**+**^) in a relative ratio of 1:2, with only the signal from counteranion **[MeB­(C_6_F_5_)_3_]** ^**–**^. After 48 h at room temperature, the same analysis revealed compounds **1c** and **1′c** in a relative ratio of 1:2.5, with only the resonances corresponding to **[MeB­(C_6_F_5_)_3_]^–^**.

^**1**^ **H NMR** (CD_2_Cl_2_, 25 °C, 400 MHz): **1c** ^**+**^, δ −0.93 (s, 6H, Al*Me*_2_), 1.04 (d,^3^ *J*_HH_ = 6.8 Hz, 12H, CH*Me*Me), 1.21 (d,^3^ *J*_HH_ = 6.8 Hz, 12H, CHMe*Me*), 2.49 (s, 6H, *Me*(CN)), 2.51 (sept,^3^ *J*_HH_ = 6.6 Hz, 4H, *CH*MeMe), 4.38 (s, 2H, CH_2_ Py-Bn), 7.08–7.50 (m, 11H, CH_Ar_), 8.26 (s, 2H, 3,5-CH_Py_). **1′c** ^**+**^, δ −0.87 (s, 3H, Al*Me*(H)), 2.40 (sept,^3^ *J*_HH_ = 6.6 Hz, 2H, *CH*MeMe), 2.53 (s, 6H, *Me*(CN)), 2.90 (sept,^3^ *J*_HH_ = 6.8 Hz, 2H, *CH*MeMe), 4.39 (s, 2H, CH_2_ Py-Bn), 7.08–7.50 (m, 11H, CH_Ar_), 8.27 (s, 2H, 3,5-CH_Py_). **[MeB­(C**_**6**_ **F**_**5**_)_**3**_]^**–**^, δ 0.43 (br s). *The Al–H signal of* **3c** *could not be located*. ^**19**^ **F NMR** (CD_2_Cl_2_, 25 °C, 376 MHz): **[HB­(C**_**6**_ **F**_**5**_)_**3**_]^**–**^, δ −134.77 (br, 2F, (*o*-*F*)), −163.59 (br, 1F, (*p-F*)) −166.72 (br, 2F, (*m-F*)). **[MeB­(C**_**6**_ **F**_**5**_)_**3**_]^**–**^: δ −133.11 (d,^3^ *J*_FF_ = 19 Hz, 2F, (*o*-*F*) [MeB­(C_6_ *F*_5_)_3_]^**–**^), −165.10 (t,^3^ *J*_FF_ = 19 Hz, 1F, (*p-F*) [MeB­(C_6_F_5_)_3_]^**–**^) −167.79 (t,^3^ *J*_FF_ = 19 Hz, 2F, (*m-F*) [MeB­(C_6_F_5_)_3_]^**–**^). ^**11**^ **B NMR** (CD_2_Cl_2_, 25 °C, 128 MHz): δ −25.27 (d,^1^ *J*_BH_ = 93 Hz, **[H** *B* **(C**_**6**_ **F**_**5**_)_**3**_]^**–**^), −14.97 (s, **[Me*B*(C_6_F_5_)_3_]^–^**).

### Single Crystal X-ray Analysis

A summary of the crystallographic data and the structure refinement results for compounds **1b·B­(C**_**6**_ **F**_**5**_ **)**_**4**_, **1c·BAr** ^**F**^_**4**_, **2b** is given in Tables S1–S3. Crystals of a suitable size for Xray diffraction analysis were coated with dry perfluoropolyether and mounted on glass fibers and fixed in a cold nitrogen stream (*T* = 193 K) to the goniometer head. Data collection was carried out on a Bruker-AXS, D8 QUEST ECO, PHOTON II area detector diffractometer, using monochromatic radiation λ­(Mo K_α_) = 0.71073 Å, by means of ω and φ scans with a width of 1.4, 0.50, and 1.5 degree, respectively. The data were reduced (SAINT V8.40B^42^) and corrected for absorption effects by the multiscan method (SADABS-2016/2 ^43^). The structures were solved by intrinsic phasing modification of direct methods (SHELXT2018/2 ^44^) and refined against all *F* ^2^ data by full-matrix least-squares techniques (SHELXL-2018/3 ^45^) minimizing *w*[*F*_0_^2^ – *F*_c_^2^],^2^ using Olex2 ^46^ as graphical interface. All non-hydrogen atoms were refined anisotropically. The hydrogen atoms were included from calculated positions and refined riding on their respective carbon atoms with isotropic displacement parameters. A search for solvent-accessible voids for structure **1b·B­(C**_**6**_ **F**_**5**_ **)**_**4**_ using SQUEEZE^47^ showed two volumes of potential solvents of 546 Å^3^ and 155 Å^3^ (174 and 38 electron count), whose solvent content could not be identified or refined with the most severe restrictions, but due to the volume and the electrons present, it would match four and one very disordered dichloromethane molecules respectively per unit cell. In general, the −CF_3_ groups of BAr^F^_4_ in **1c·BAr** ^**F**^_**4**_ present positional disorder, so four of them were modeled as two components of the disorder with their respective occupancy coefficients. Therefore, it was also necessary to use some geometric restraints (SADI, SIMU, RIGU) during the structure refinement to ensure a sensible geometry. A search for solvent-accessible voids for structure **1c·BAr** ^**F**^_**4**_ using SQUEEZE^46^ showed a volume of potential solvents of 508 Å^3^ (168 electron count), whose solvent content could not be identified or refined with the most severe restrictions. Due to the volume and the electrons present, it would match four very disordered dichloromethane molecules per unit cell. The corresponding CIF data represent SQUEEZE treated structures with the solvent molecules handling as a diffuse contribution to the overall scattering, without specific atom position and excluded from the structural model. The SQUEEZE results were appended to the CIF. Crystallizing compound **2b** is challenging due to their instability, and it appears somewhat twinned, exhibiting at least four additional minority domains. Refining these structures using HKLF 5 and BASF did not yield improved results. Consequently, due to the imperfections in the crystals and the presence of some disorder, as previously discussed and resolved, R-values is somewhat elevated. The corresponding crystallographic data were deposited with the Cambridge Crystallographic Data Centre as supplementary publications. CCDC 2441156 (**1b·B** **(C_6_F_5_)**_**4**_), 2441157 (**1c·BAr** ^**F**^_**4**_), and 2441158 for **2b** contain the supplementary crystallographic data for this paper. The data can be obtained free of charge *via:* https://www.ccdc.cam.ac.uk/structures/.

## Supplementary Material



## Notes

Instrumentation and procedures, NMR spectra of new compounds (PDF), NMR monitoring of reactions and X-ray structural data (PDF)
