# Supplementary material for: Turning the Tables: Ligand-Centered Hydride Shuttling in Organometallic BIP–Al Systems
Source: Inorg Chem. 2025 Jul 24;64(30):15760–73. doi: 10.1021/acs.inorgchem.5c02587 (PMC12326362; doi:10.1021/acs.inorgchem.5c02587)
Supplement: Supplementary file 1 [file ic5c02587_si_001.pdf]

Supplementary Information for

Turning the Tables: Ligand-Centered Hydride Shuttling in Organometallic BIP–Al Systems.

Juan Manuel Delgado-Collado, Francisco José Fernández de Córdova, Pilar Palma, Juan Cámpora and Antonio Rodríguez-Delgado**

Instituto de Investigaciones Químicas, CSIC-Universidad de Sevilla, c/Américo Vespucio, 49, 41092 Sevilla, Spain

1. Instrumentation and procedures S2-S3
2. NMR spectra of new compounds S3-S12
   2.1 NMR spectra of **1c·BAr^F$_4$** S3-S4
   2.2 NMR spectra of **2a** S5
   2.3 NMR spectra of **2b** S6
   2.4 NMR spectra of **1a·B(C_6F_5)$_4$** S7-S8
   2.5 NMR spectra of **1b·B(C_6F_5)$_4$** S9-S10
   2.6 NMR spectra of **1c·B(C_6F_5)$_4$** S11-S12
3. NMR monitoring of reactions S13-S24
   3.1 Reaction of **2a** with $B(C_6F_5)_3$ in CD_2Cl_2. Formation of **1a·HB(C_6F_5)$_3$** and **1'a·MeB(C_6F_5)$_3$** S13-S14
   3.2 Reaction of **2a** with $B(C_6F_5)_3$ in C_6D_6. Formation of **1a·HB(C_6F_5)$_3$** and **1'a·MeB(C_6F_5)$_3$** S15-S16
   3.3 Reaction of **2b** with $B(C_6F_5)_3$ in CD_2Cl_2. Formation of **1b·HB(C_6F_5)$_3$** and **1'b·EtB(C_6F_5)$_3$** S17-S18
   3.4 Reaction of **1c·BAr^F$_4$** with $LiHBEt_3$. Formation of **2c** and **3c** S19
   3.5 Reaction of the mixture of **2c** and **3c** with $B(C_6F_5)_3$ S20-S21
   3.6 EPR spectrum S22
   3.7 Reaction of **2a** with dry O_2 S23
   3.8 Reaction of **2a** with MeOH S24
4. X-ray structural data S25-S30

1. Instrumentation and procedures

Most of the compounds presented in this work are highly sensitive to oxygen and moisture. Therefore, inert atmosphere Schlenk techniques or/and a N_2-filled glovebox were routinely used in manipulations and procedures.

Solvent preparation: Dichloromethane, N-hexane, pentane and diethylether were rigorously degassed, dried, and distilled immediately prior to use.

NMR Spectroscopy: NMR spectra were recorded on Bruker equipment, models DPX 300, DPX-400, AVIIIHD 400 nanobay or a AVIII 500, typically at 298 K, unless stated otherwise. The chemical shifts of the ^1H and $^{13}C\{^1H\}$ spectra are referenced to tetramethylsilane (TMS). Spectral assignations were routinely helped with bidimensional (2D) ^1H ^1H COSY, ^1H-^{13}C HMBC and HSQC heterocorrelation spectra. $^{19}F\{^1H\}$ NMR spectra used external referencing with 1% $CFCl_3$ sample in $CHCl_3$. $^{11}B\{^1H\}$ and ^{11}B NMR spectra were externally referenced to 5% $BF_3{\cdot}OEt_2$ sample in C_6D_6. Chemical shifts (δ) are reported in ppm and coupling constants (J) in Hz. Prior use, CD_2Cl_2 was dried over CaH_2, distilled under low pressure ($< 1 \times 10^{-2}$ mbar), and stored under nitrogen. Deuterated benzene C_6D_6 was dried over sodium, distilled under low pressure ($< 1 \times 10^{-2}$ mbar), and stored under nitrogen over Na mirror.

EPR spectroscopy: Electronic Paramagnetic Resonance spectra were recorded with a Bruker Elexsys spectrometer working in X-Band (~ 9.47 GHz) equipped with a helium gas-flow cryostat. EPR samples were dissolved in dichloromethane under a nitrogen atmosphere and transferred to quartz tubes that were subsequently sealed.

Electrospray ionization mass spectrometry (ESI-MS): Electrospray ionisation mass spectra were recorded with a Bruker Esquire6000 spectrometer with ion trap analyser, capable of determining m/z ratios up to 6000 Dalton.

Elemental Analysis: CHN microanalyses were executed in a LECO TruSpec CHN elementary analyzer in the Instituto de Investigaciones Químicas (CSIC-Universidad de Sevilla).

X-ray diffraction studies: These measurements were carried in a Bruker-AXS, D8 Quest ECO diffractometer equipped with a micro-focus IμS 3.0 source, using graphite monochromatized Mo radiation λ(Mo Kα) = 0.71073 Å and with an area detector Bruker

Photon II 14 - CPAD in the Instituto de de Investigaciones Químicas (CSIC-Universidad de Sevilla).

2. NMR spectra of new compounds

2.1 NMR spectra of 1c·BAr$^F_4$

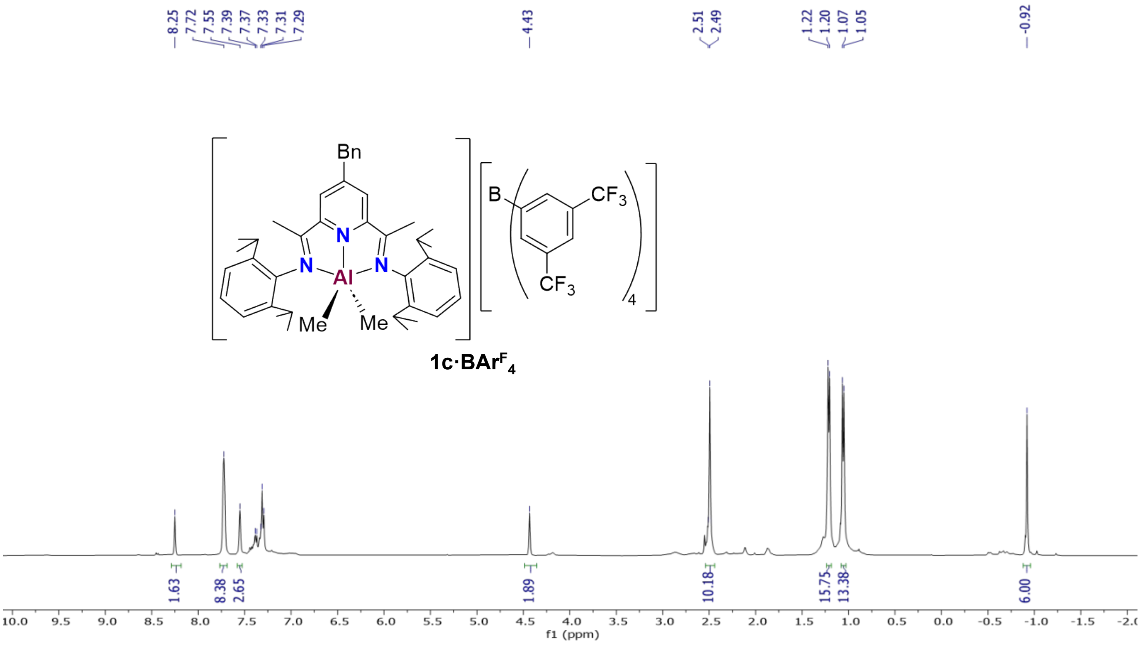

Figure S1. **^{1}H-NMR** (CD_2Cl_2, 25 °C, 400 MHz) spectrum of **1c·BAr$^F_4$.**

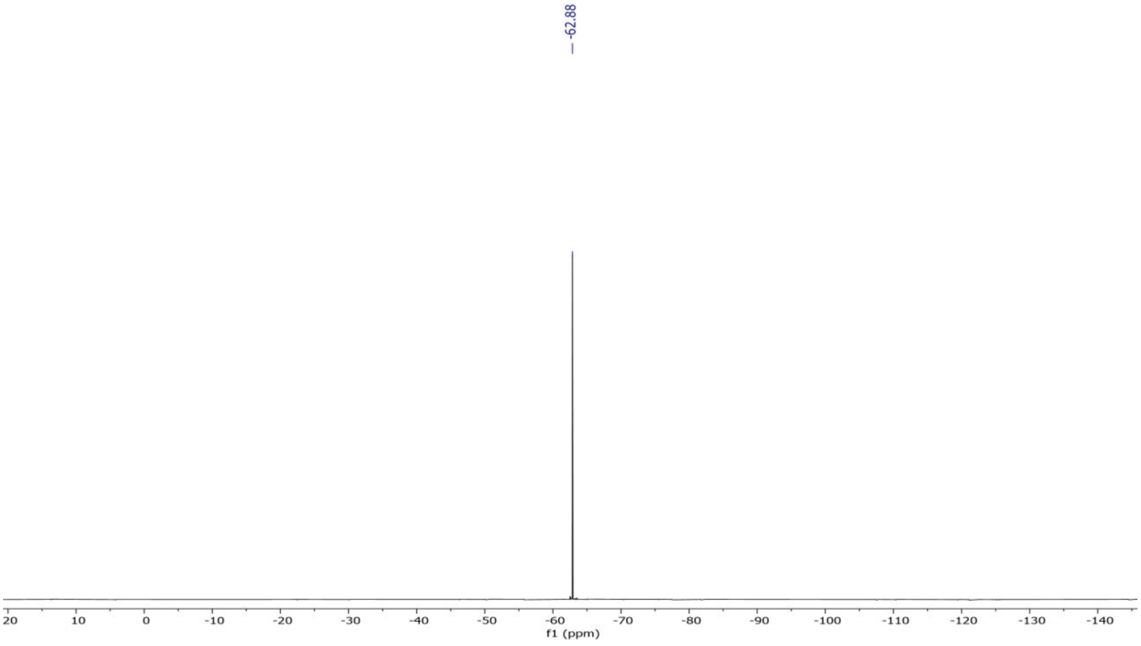

Figure S2. **^{19}F{^{1}H}-NMR** (CD_2Cl_2, 25 °C, 376 MHz) spectrum of **1c·BAr$^F_4$.**

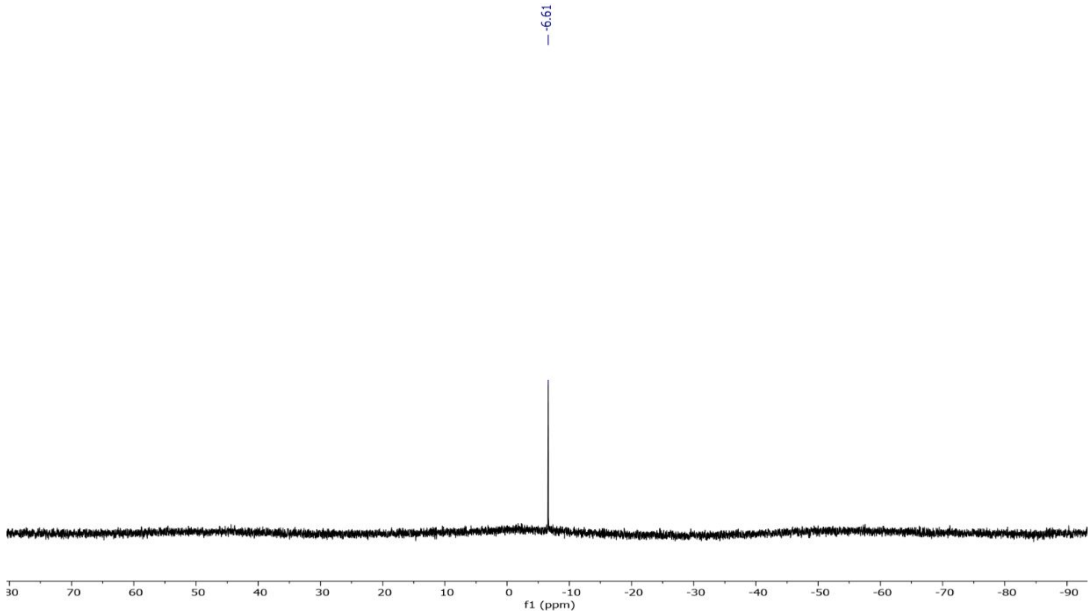

Figure S3. $^{11}B\{^1H\}$-NMR (CD_2Cl_2, 25 °C, 128 MHz) spectrum of **1c·BAr^F$_4$.**

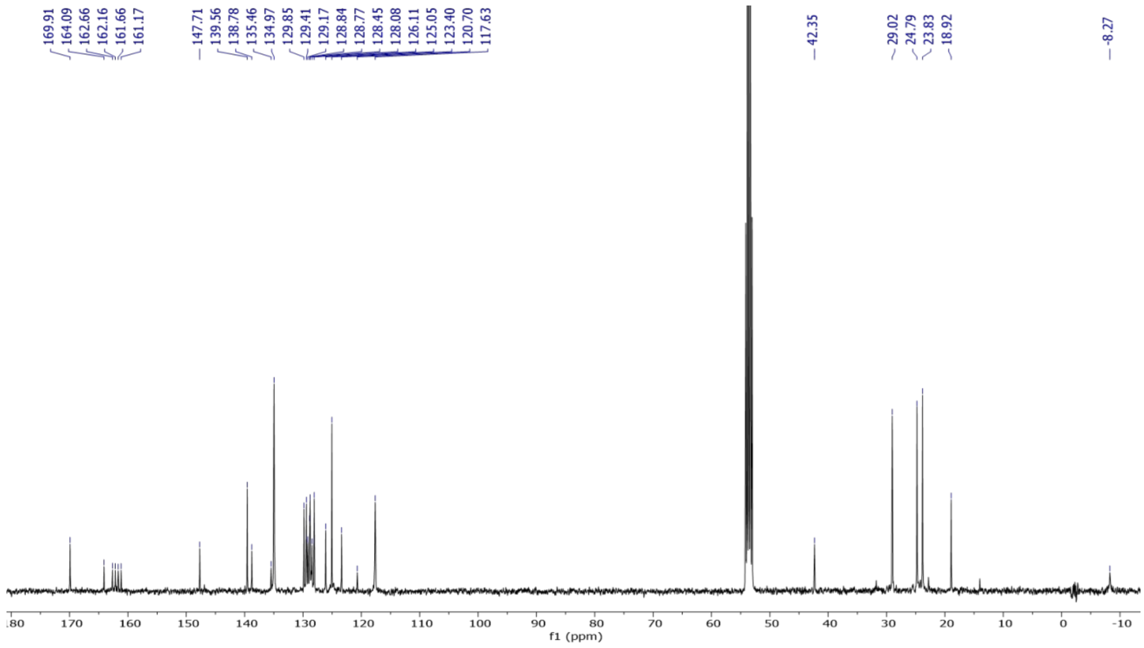

Figure S4. $^{13}C\{^1H\}$-NMR (CD_2Cl_2, 25 °C, 100 MHz) spectrum of **1c·BAr^F$_4$**.

2.2 NMR spectra of 2a

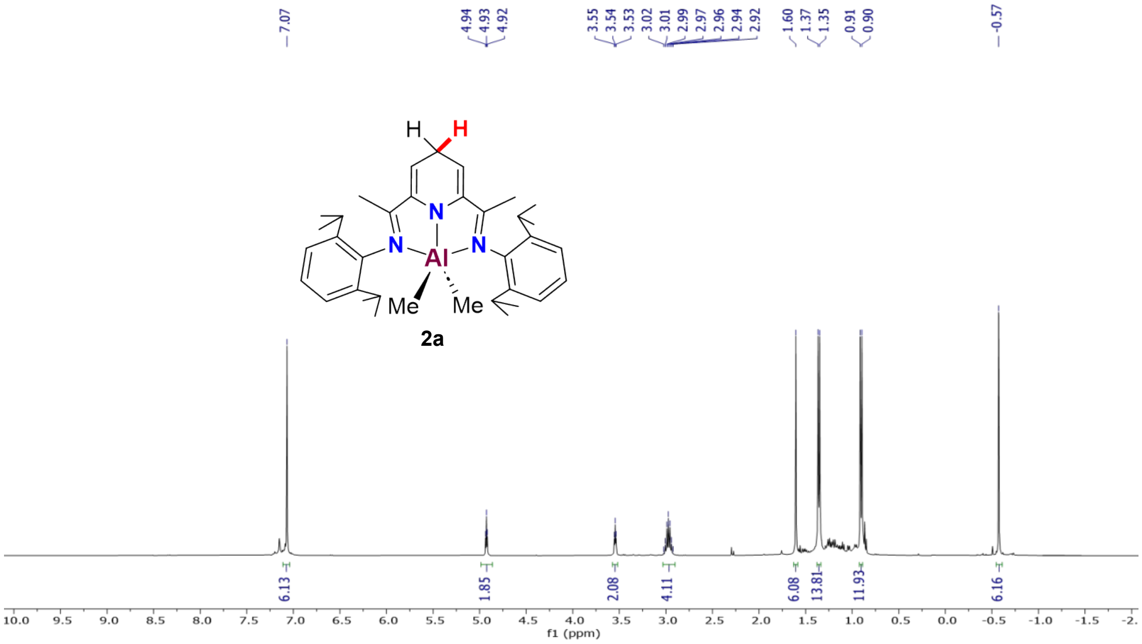

Figure S5. **^{1}H-NMR** (C_6D_6, 25 °C, 400 MHz) spectrum of **2a.**

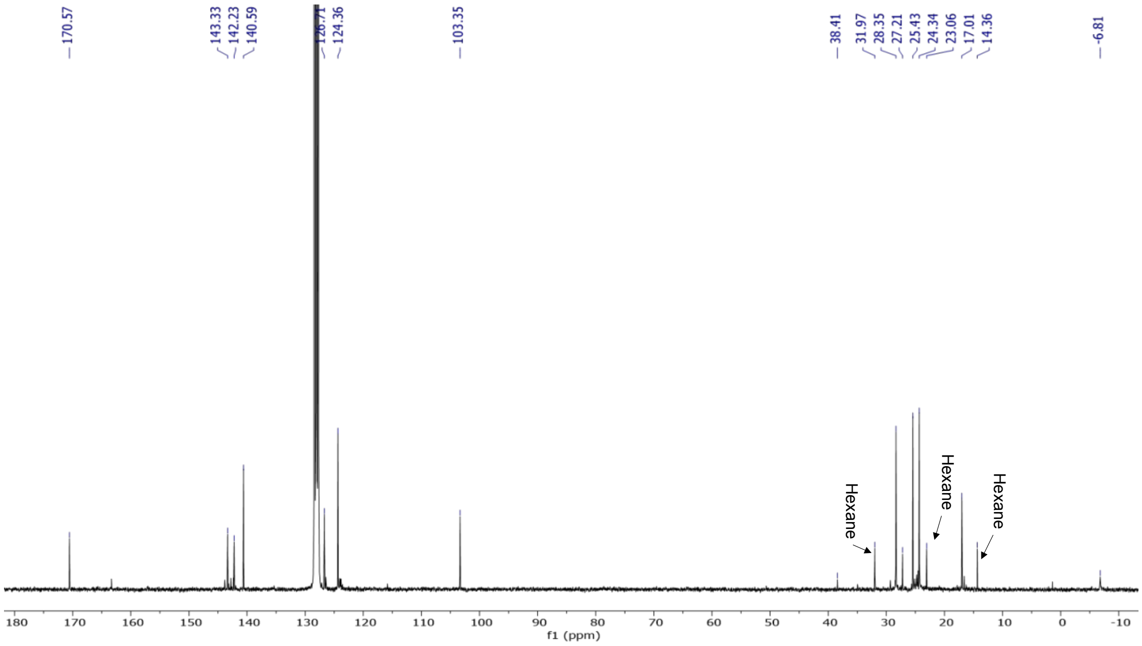

Figure S6. **^{13}C{^{1}H}-NMR** (C_6D_6, 25 °C, 100 MHz) spectrum of **2a**.

2.3 NMR spectra of 2b

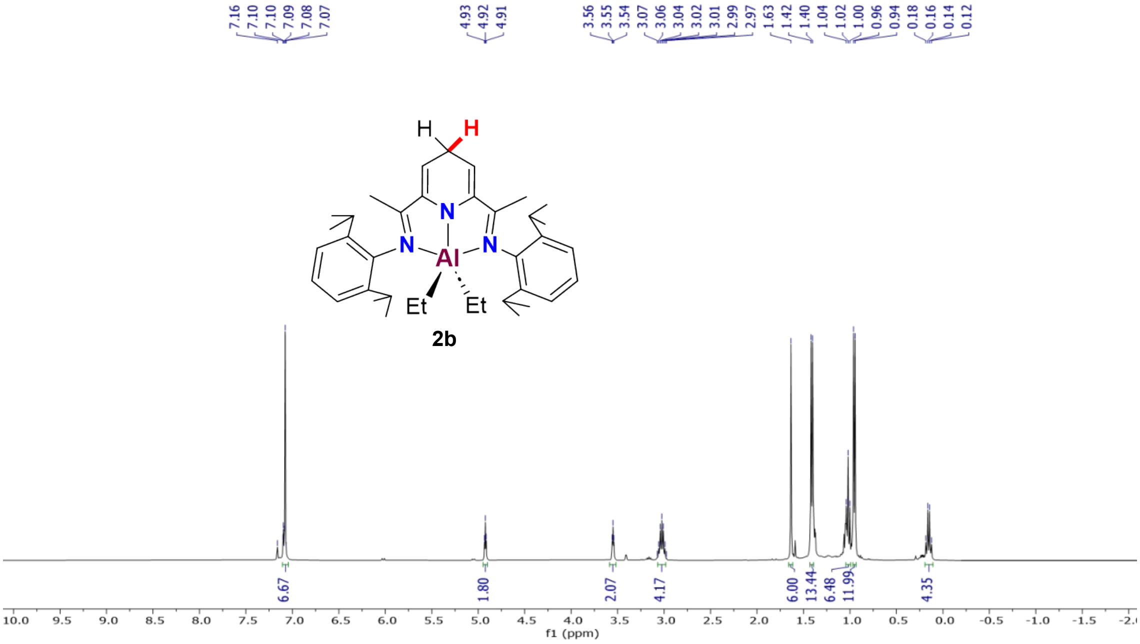

Figure S7. **^{1}H-NMR** (C_6D_6, 25 °C, 400 MHz) spectrum of **2b**.

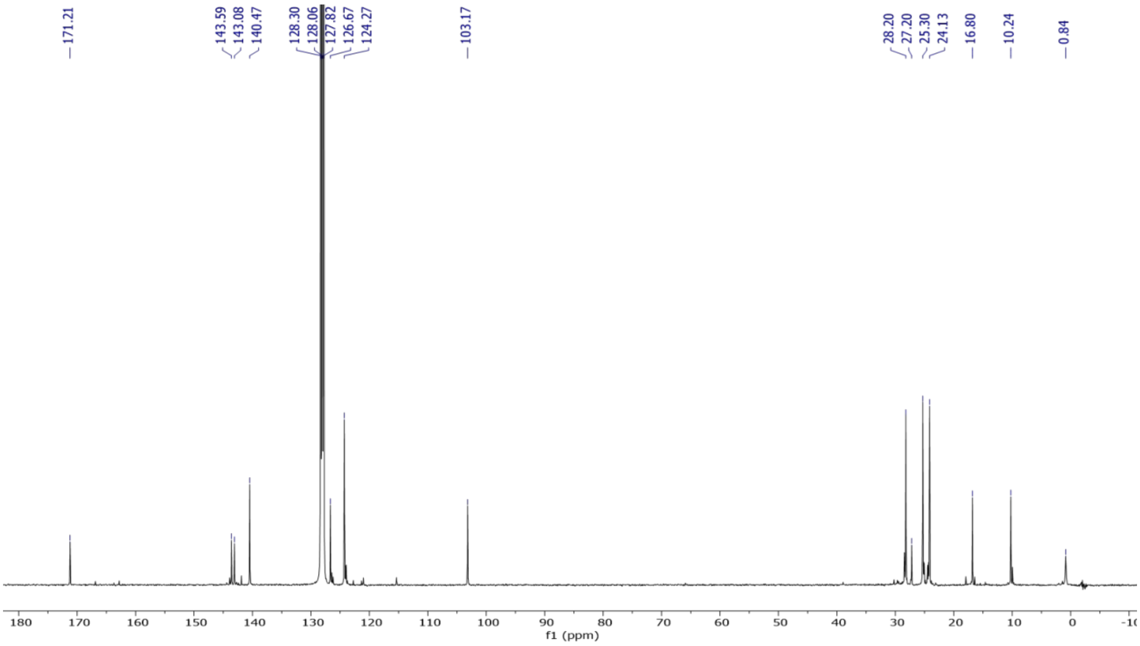

Figure S8. **^{13}C{^{1}H}-NMR** (C_6D_6, 25 °C, 100 MHz) spectrum of **2b**.

2.4 NMR spectra of 1a·$B(C_6F_5)_4$.

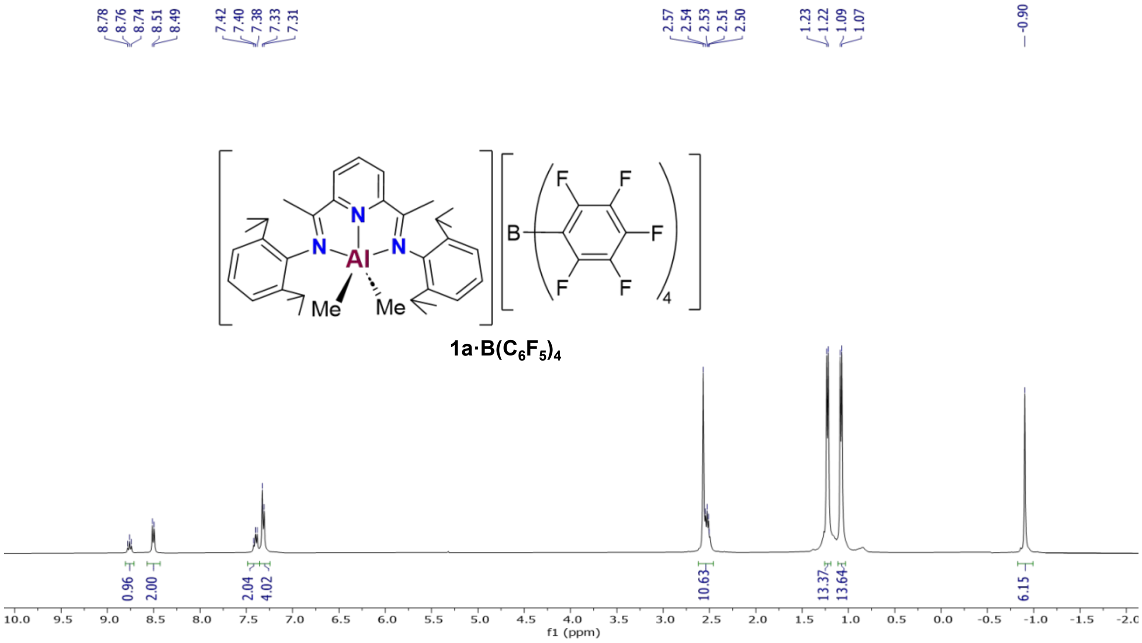

Figure S9. **^{1}H-NMR** (CD_2Cl_2, 25 °C, 400 MHz) spectrum of **1a·$B(C_6F_5)_4$.**

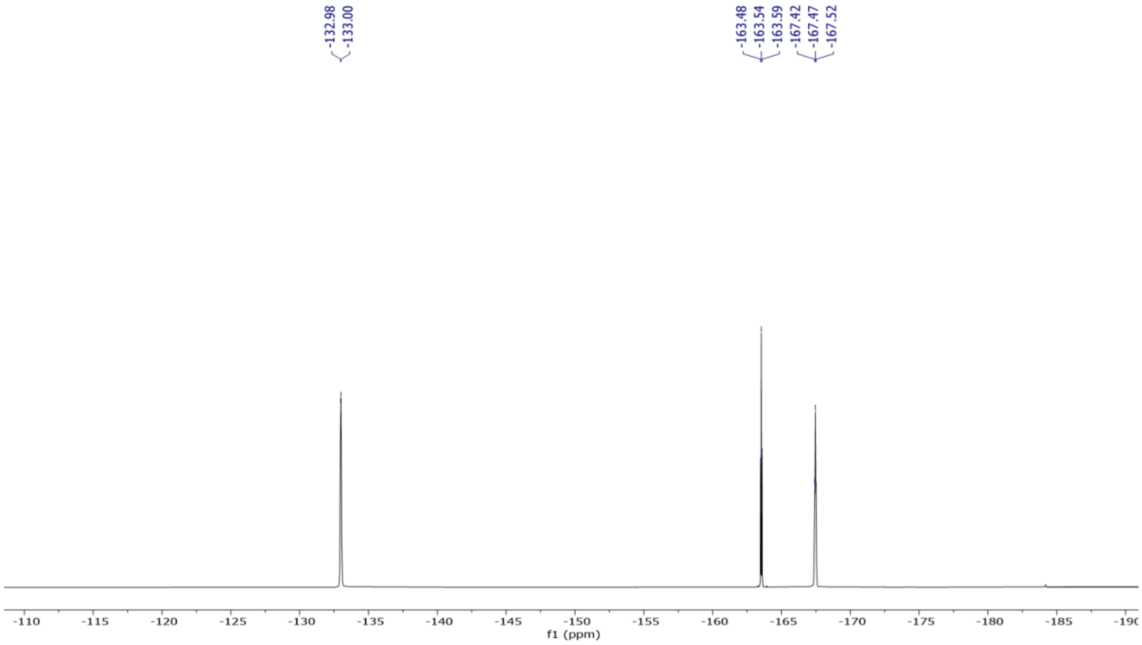

Figure S10. **^{19}F{^{1}H}-NMR** (CD_2Cl_2, 25 °C, 376 MHz) spectrum of **1a·$B(C_6F_5)_4$.**

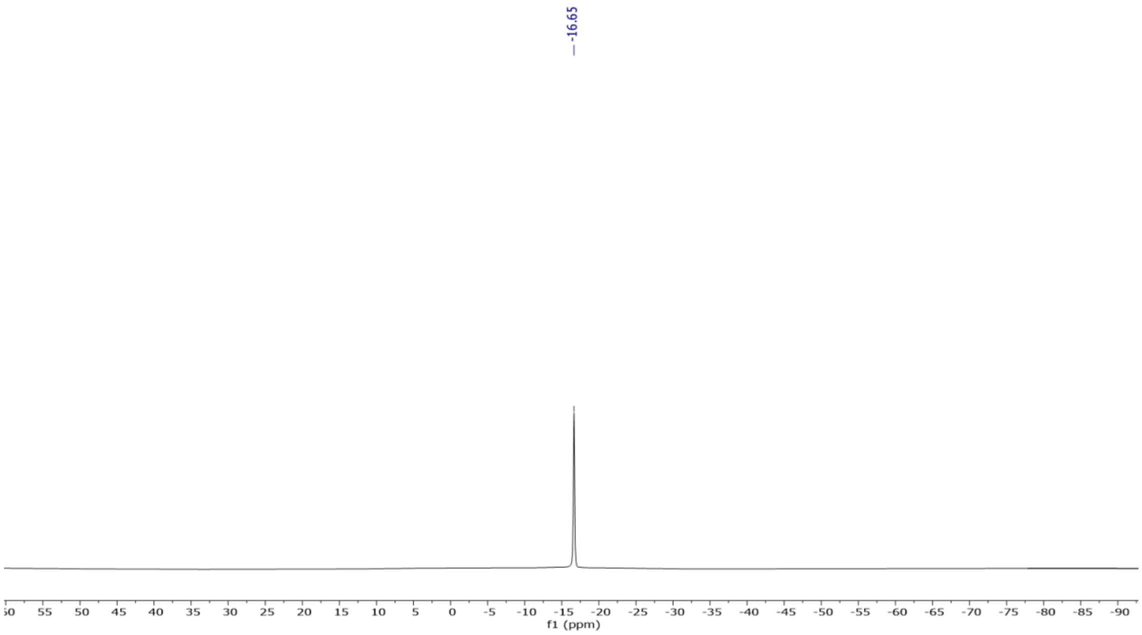

Figure S11. ^{11}**B{**^{1}**H}-NMR** (CD_2Cl_2, 25 °C, 128 MHz) spectrum of **1a·B(C$_6$F$_5$)$_4$.**

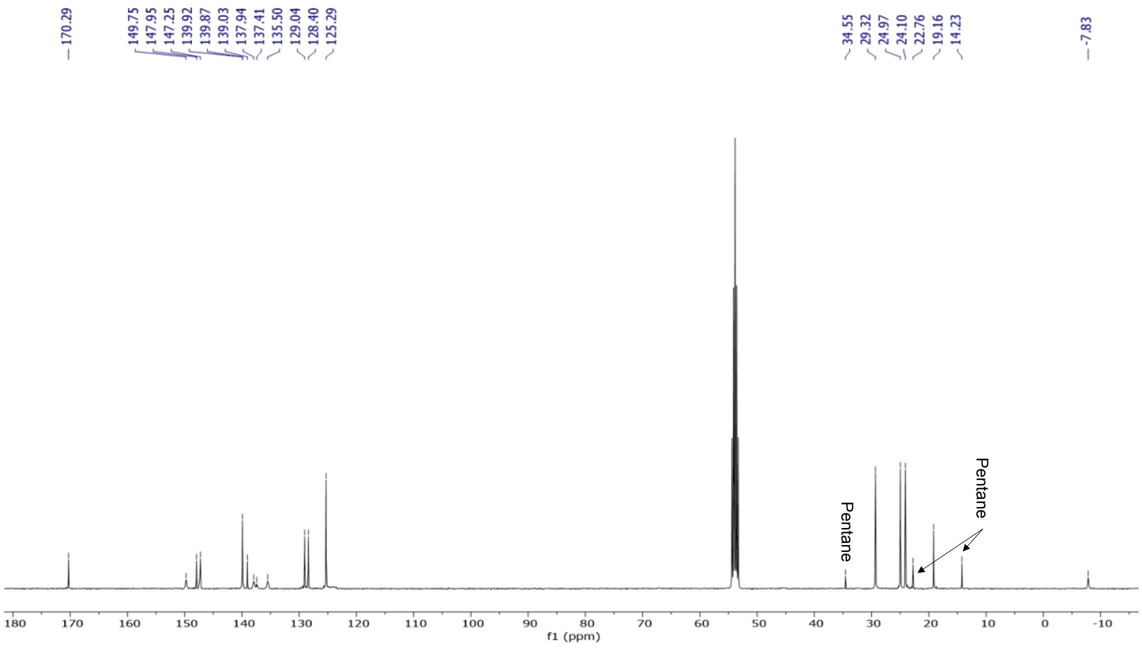

Figure S12.^{13}**C{**^{1}**H}-NMR** (CD_2Cl_2, 25 °C, 100 MHz) spectrum of **1a·B(C$_6$F$_5$)$_4$.**

2.5 NMR spectra of 1b·B(C_6F_5)$_4$.

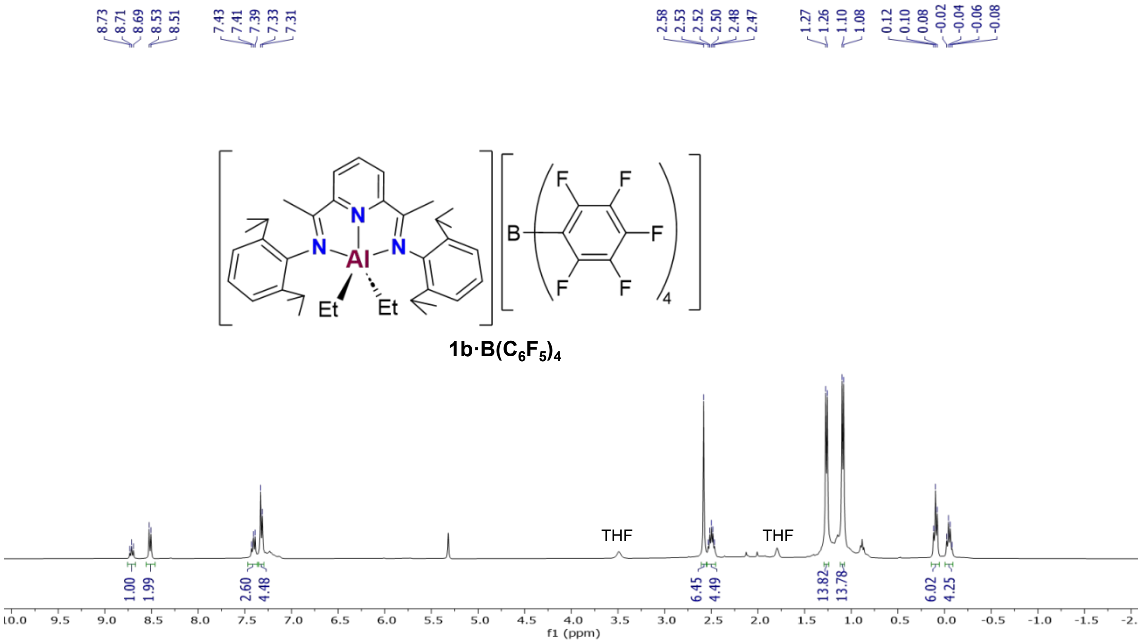

Figure S13. **^{1}H-NMR** (CD_2Cl_2, 25 °C, 400 MHz) spectrum of **1b·B(C_6F_5)$_4$.**

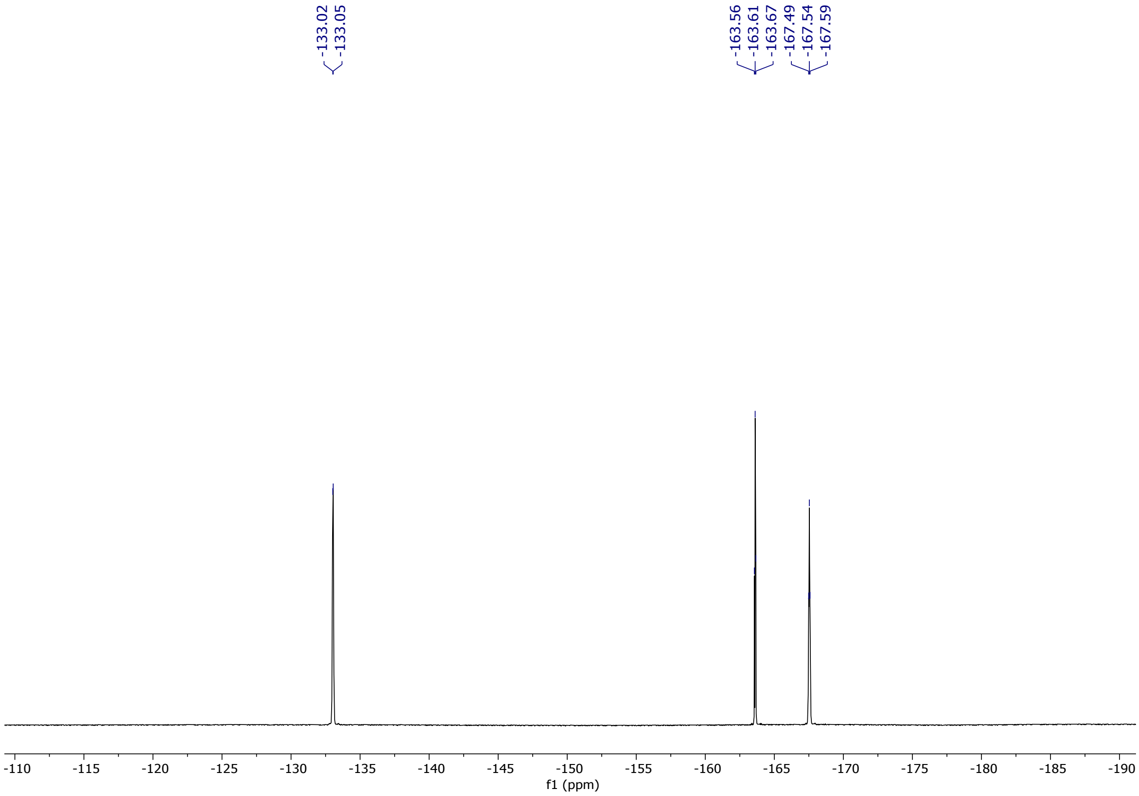

Figure S14. **^{19}F{^{1}H}-NMR** (CD_2Cl_2, 25 °C, 376 MHz) spectrum of **1b·B(C_6F_5)$_4$**.

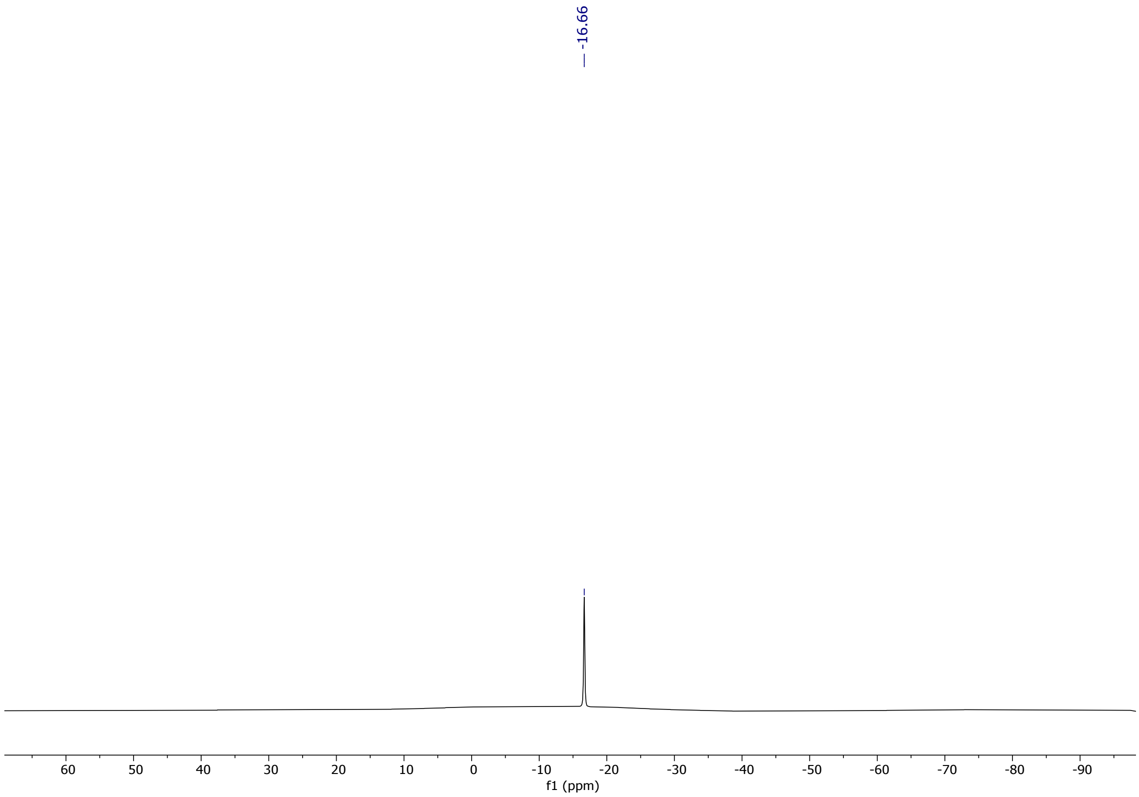

Figure S15. ^{11}**B{**^{1}**H}-NMR** (CD_2Cl_2, 25 °C, 128 MHz) spectrum of **1b·B(C_6F_5)$_4$**.

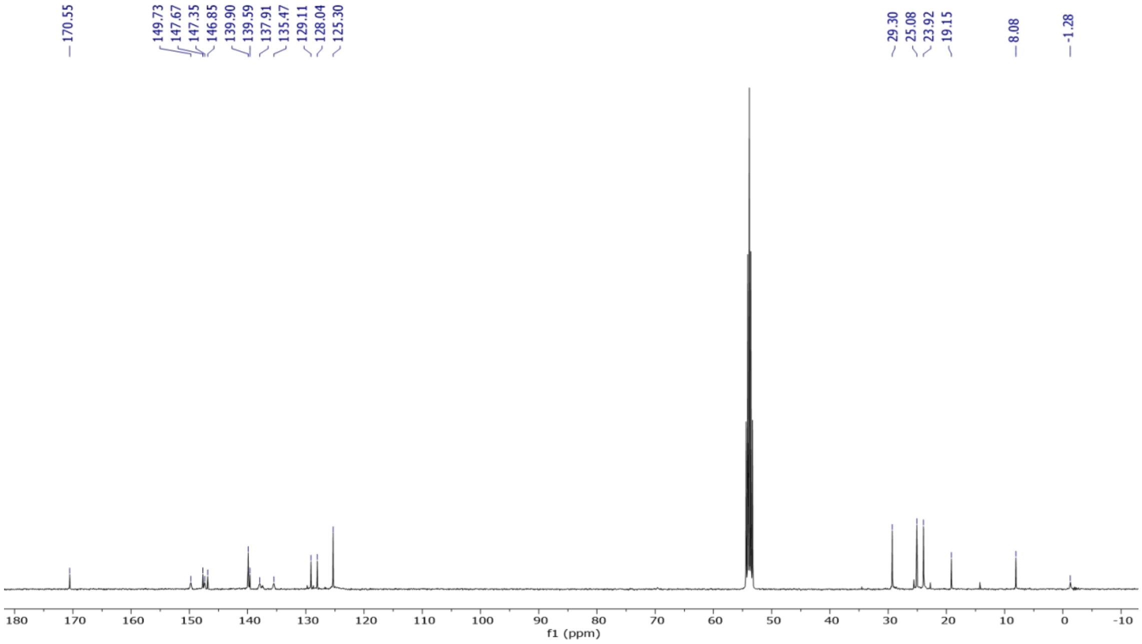

Figure S16. ^{13}**C{**^{1}**H}-NMR** (CD_2Cl_2, 25 °C, 100 MHz) spectrum of **1b·B(C_6F_5)$_4$**.

2.6 NMR spectra of $1c \cdot B(C_6F_5)_4$.

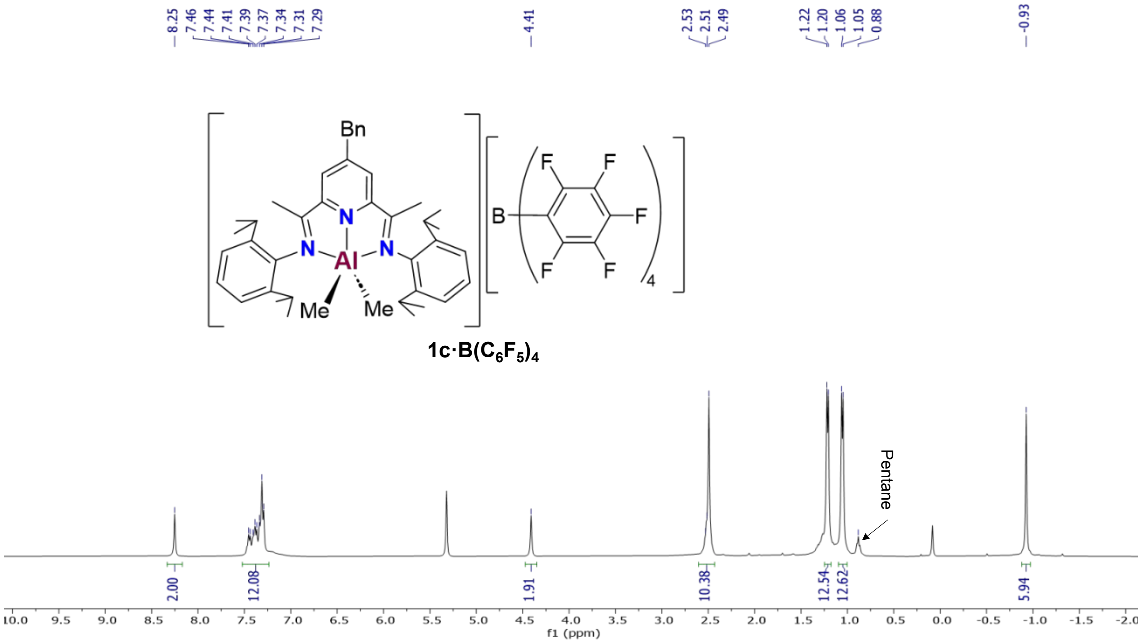

Figure S17. **^{1}H-NMR** (CD_2Cl_2, 25 °C, 400 MHz) spectrum of **$1c \cdot B(C_6F_5)_4$**.

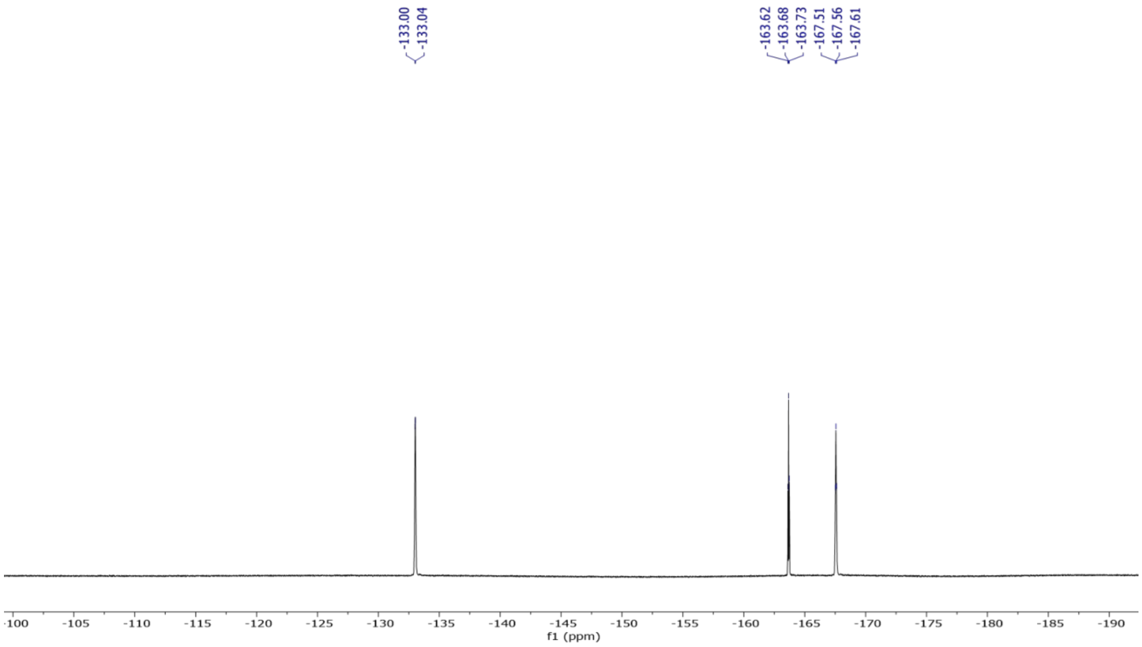

Figure S18. **$^{19}F\{^1H\}$-NMR** (CD_2Cl_2, 25 °C, 376 MHz) spectrum of **$1c \cdot B(C_6F_5)_4$**.

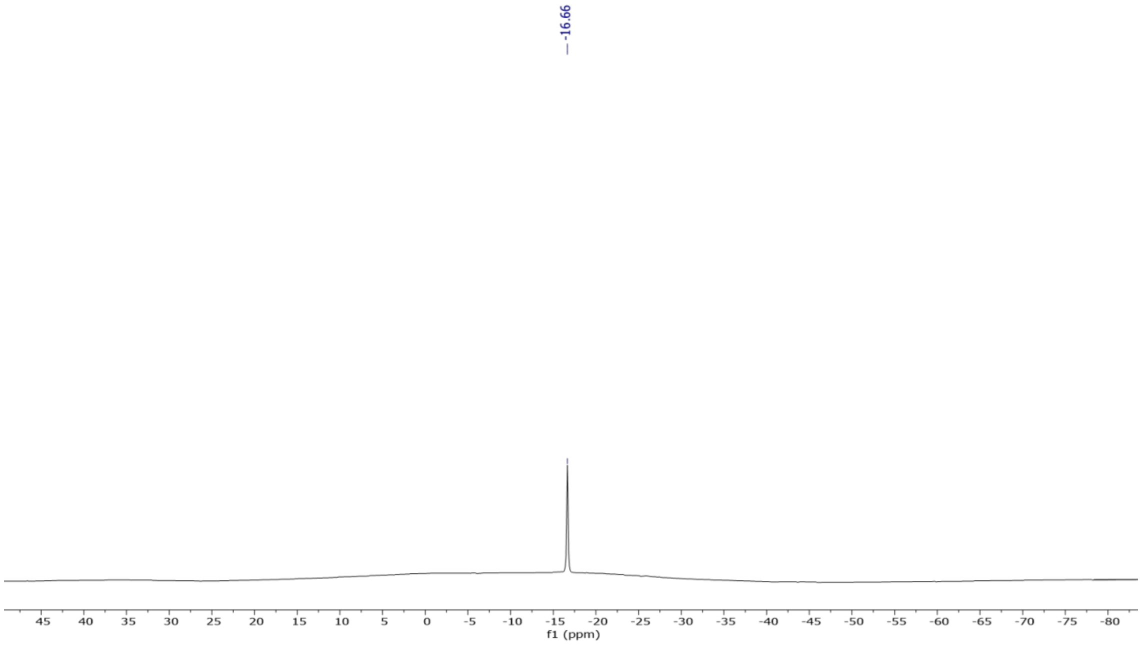

Figure S19. ^{11}B{^{1}H}-NMR (CD_2Cl_2, 25 °C, 128 MHz) spectrum of **1c·B(C_6F_5)$_4$.**

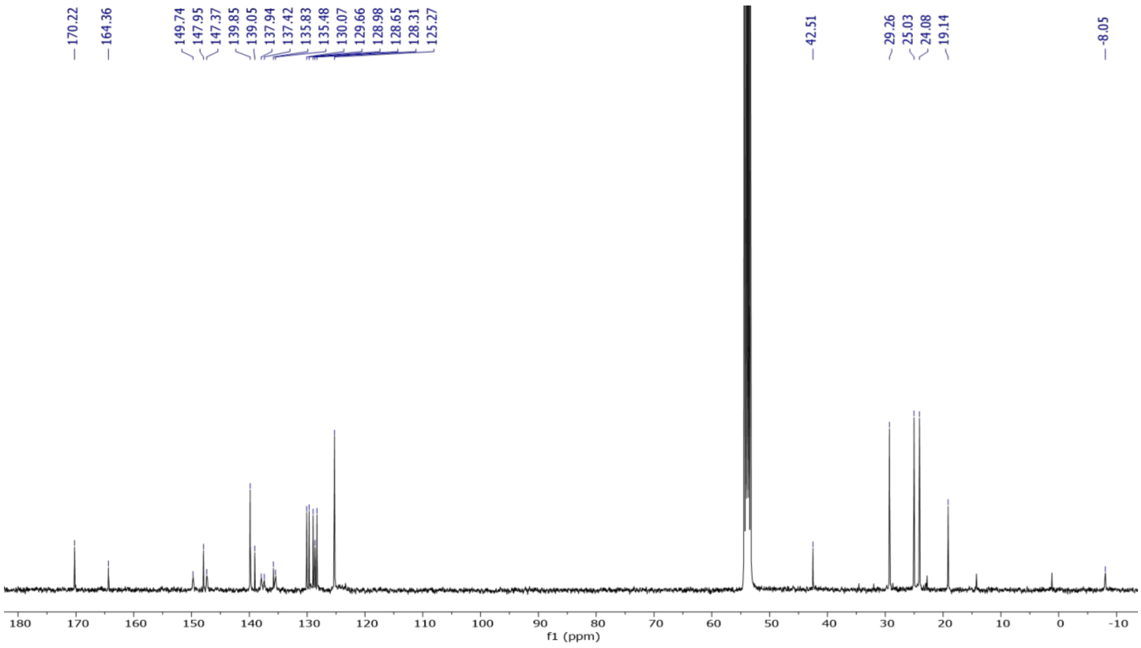

Figure S20. ^{13}C{^{1}H}-NMR (CD_2Cl_2, 25 °C, 100 MHz) spectrum of **1c·B(C_6F_5)$_4$.**

3. NMR monitoring of reactions

3.1 Reaction of 2a with $B(C_6F_5)_3$ in CD_2Cl_2. Formation of 1a·$HB(C_6F_5)_3$ and 1'a·$MeB(C_6F_5)_3$.

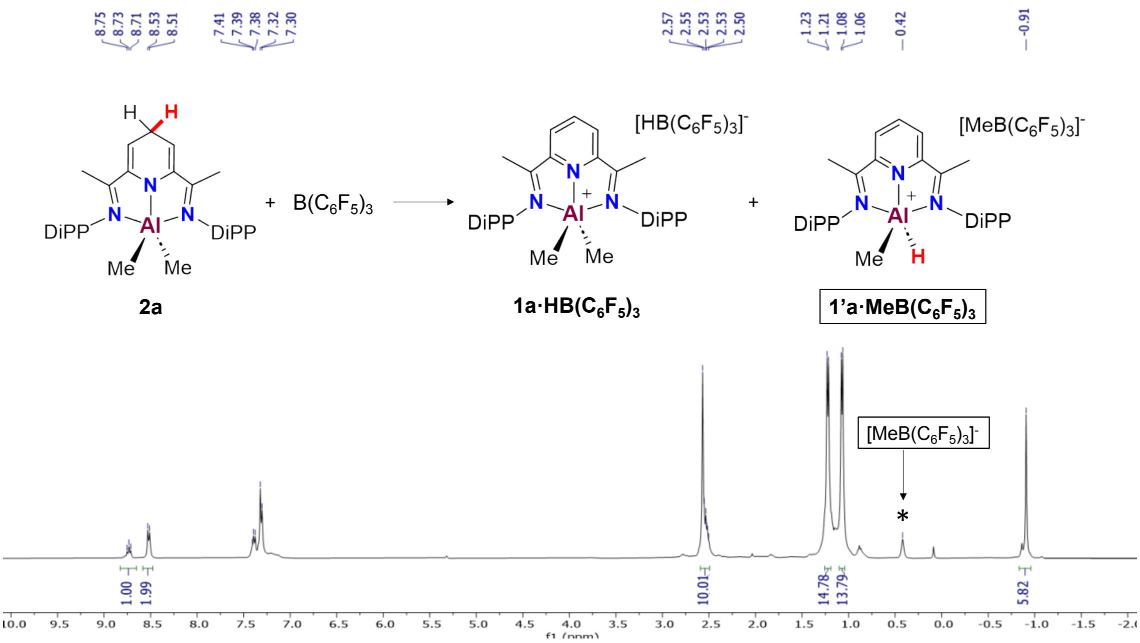

Figure S21. **^{1}H-NMR** (CD_2Cl_2, 25 °C, 400 MHz) spectrum of the reaction product of **2a** with $B(C_6F_5)_3$ in CD_2Cl_2. The signal marked with asterisk (*) corresponds to **$[MeB(C_6F_5)_3]^-$**.

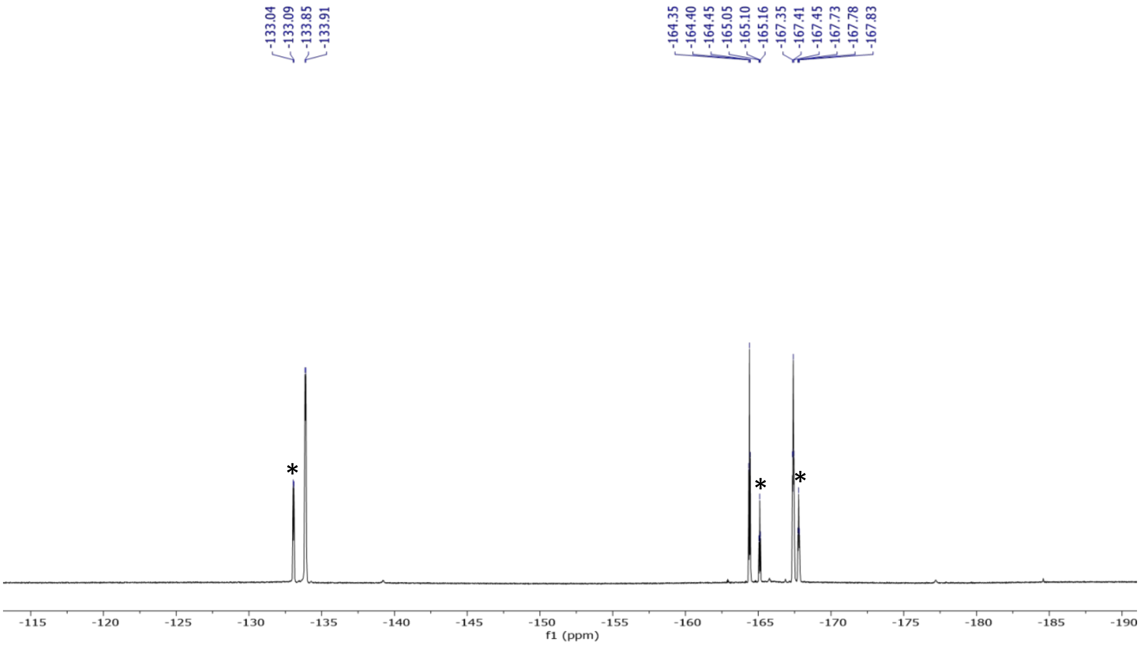

Figure S22. **^{19}F{^{1}H}-NMR** (CD_2Cl_2, 25 °C, 376 MHz) spectrum of the reaction product of **2a** with $B(C_6F_5)_3$ in CD_2Cl_2. The signs marked with asterisk (*) correspond to **$[MeB(C_6F_5)_3]^-$**.

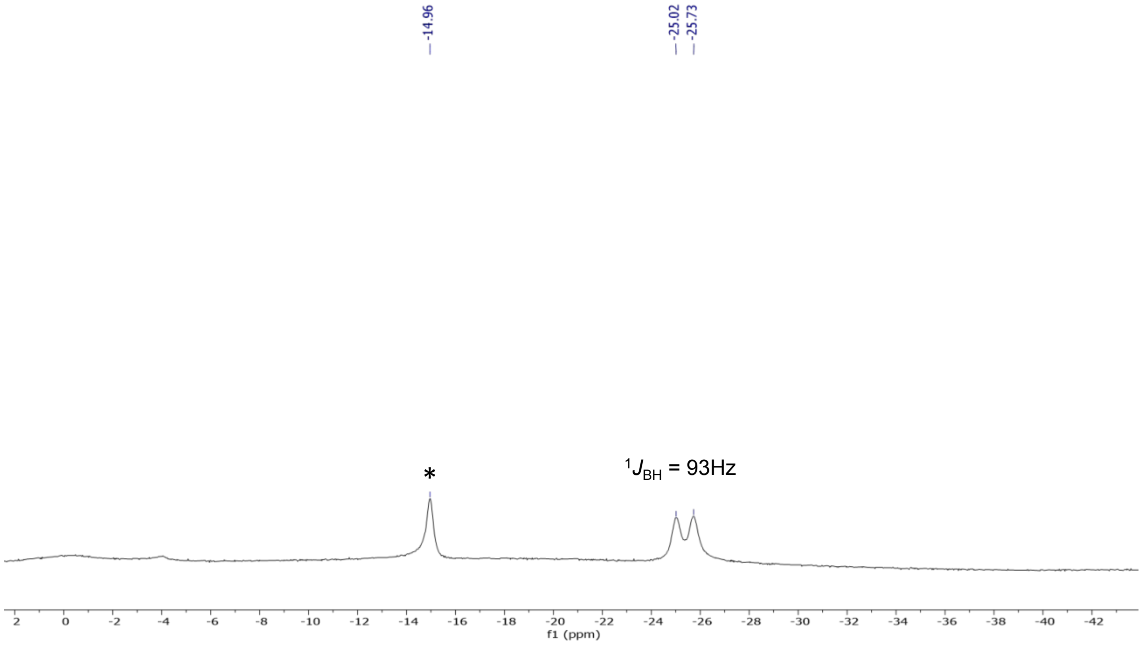

Figure S23. ^{11}**B-NMR** (CD_2Cl_2, 25 °C, 128 MHz) spectrum of the reaction product of **2a** with $B(C_6F_5)_3$ in CD_2Cl_2. The signal marked with asterisk (*) corresponds to **[MeB(C$_6$F$_5$)$_3$]$^-$**

3.2 Reaction of 2a with $B(C_6F_5)_3$ in C_6D_6. Formation of 1a·$HB(C_6F_5)_3$ and 1’a·$MeB(C_6F_5)_3$.

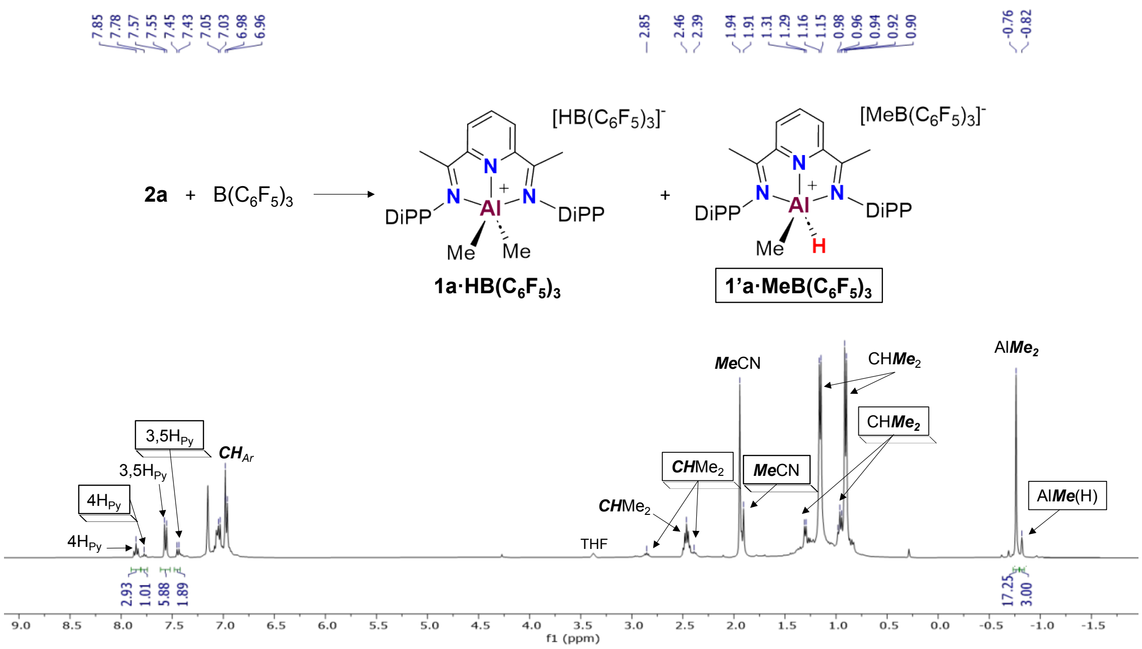

Figure S24. **^{1}H-NMR** (C_6D_6, 25 °C, 400 MHz) spectrum of the reaction product of **2a** with $B(C_6F_5)_3$ in C_6D_6.

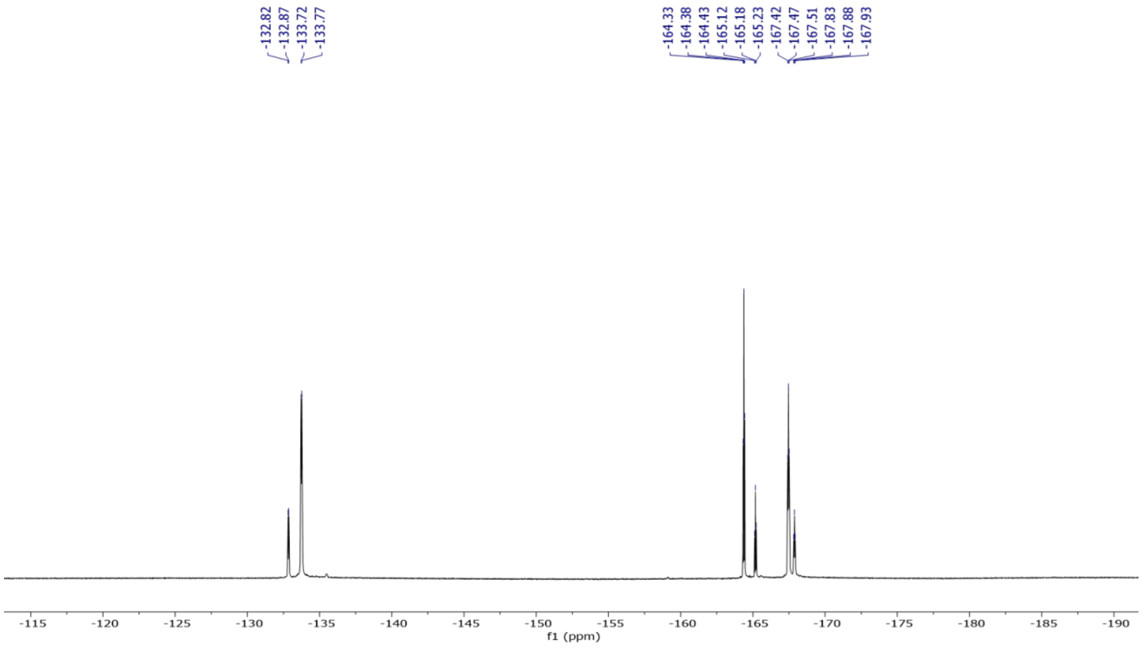

Figure S25. **^{19}F{^{1}H}-NMR** (C_6D_6, 25 °C, 376 MHz) spectrum of the reaction product of **2a** with $B(C_6F_5)_3$ in C_6D_6.

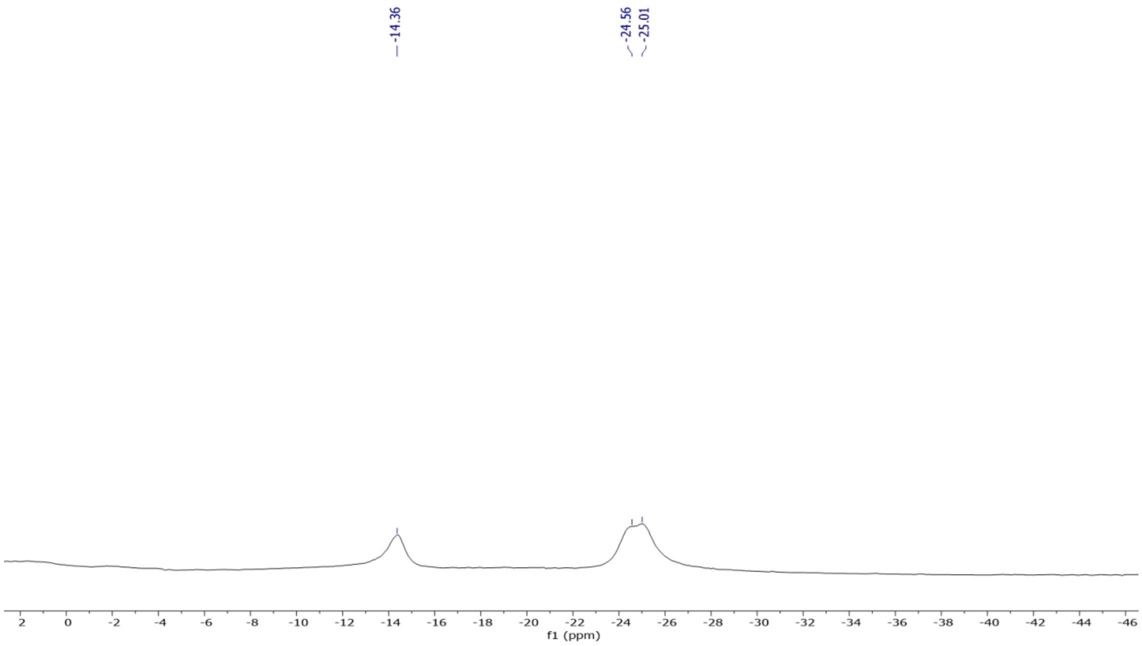

Figure S26. **^{11}B-NMR** (C_6D_6, 25 °C, 128 MHz) spectrum of the reaction product of **2a** with $B(C_6F_5)_3$ in C_6D_6.

3.3 Reaction of 2b with $B(C_6F_5)_3$ in CD_2Cl_2. Formation of 1b·$HB(C_6F_5)_3$ and 1'b·$EtB(C_6F_5)_3$.

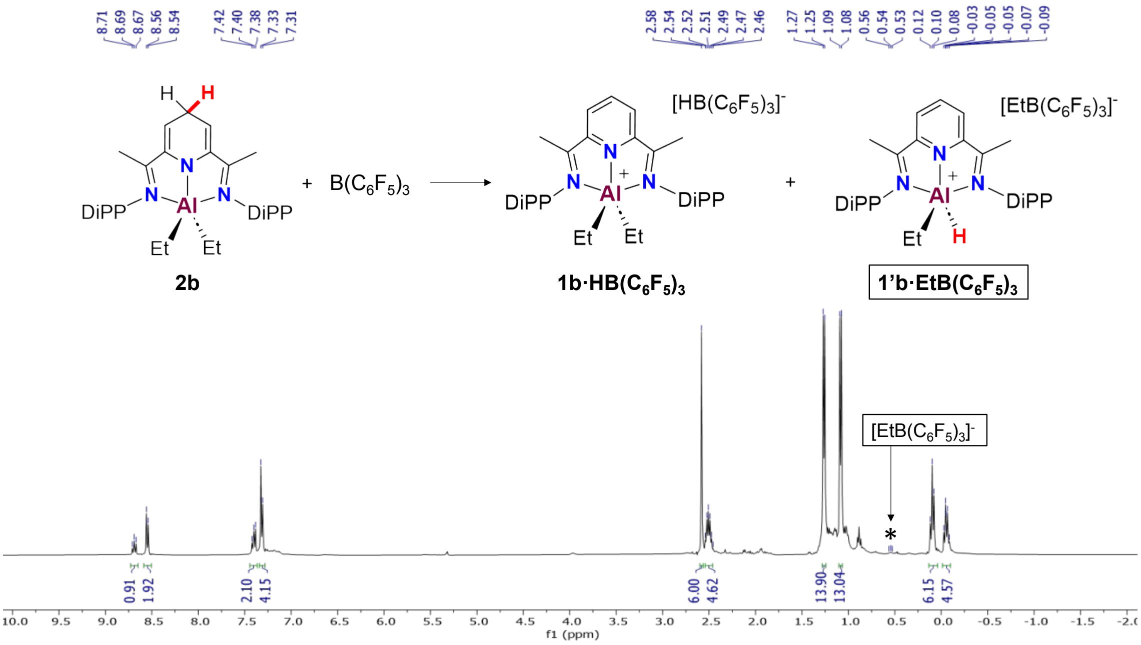

Figure S27. **^{1}H-NMR** (CD_2Cl_2, 25 °C, 400 MHz) spectrum of the reaction product of **2b** with $B(C_6F_5)_3$ in CD_2Cl_2. The signal marked with asterisk (*) corresponds to **$[EtB(C_6F_5)_3]^-$**.

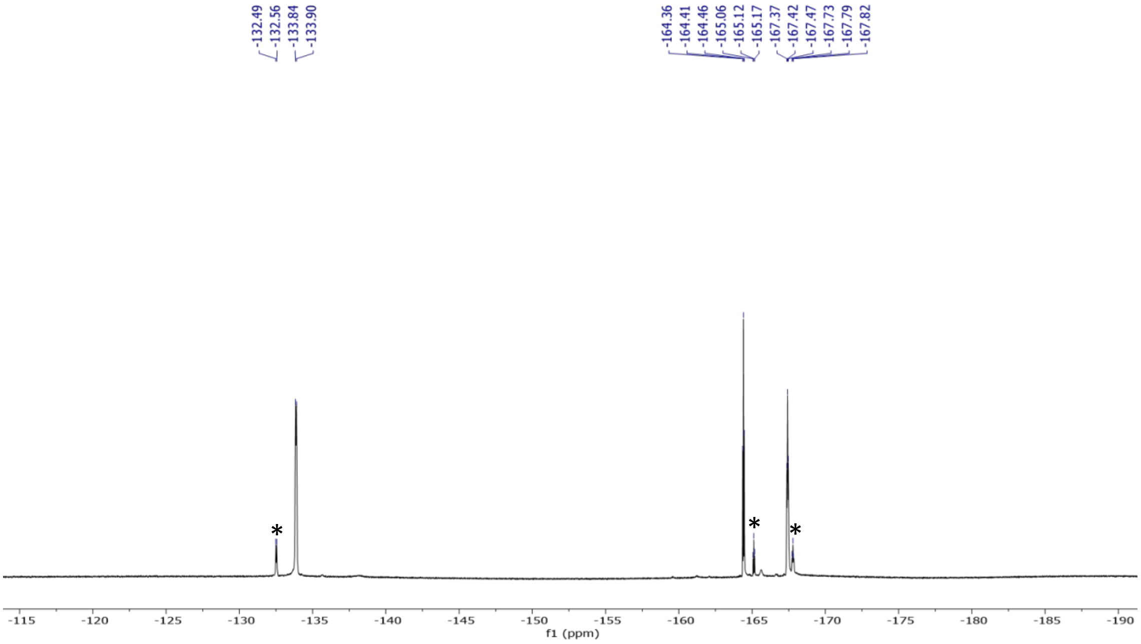

Figure S28. **$^{19}F\{^1H\}$-NMR** (CD_2Cl_2, 25 °C, 376 MHz) spectrum of the reaction product of **2b** with $B(C_6F_5)_3$ in CD_2Cl_2. The signs marked with asterisk (*) correspond to **$[EtB(C_6F_5)_3]^-$**.

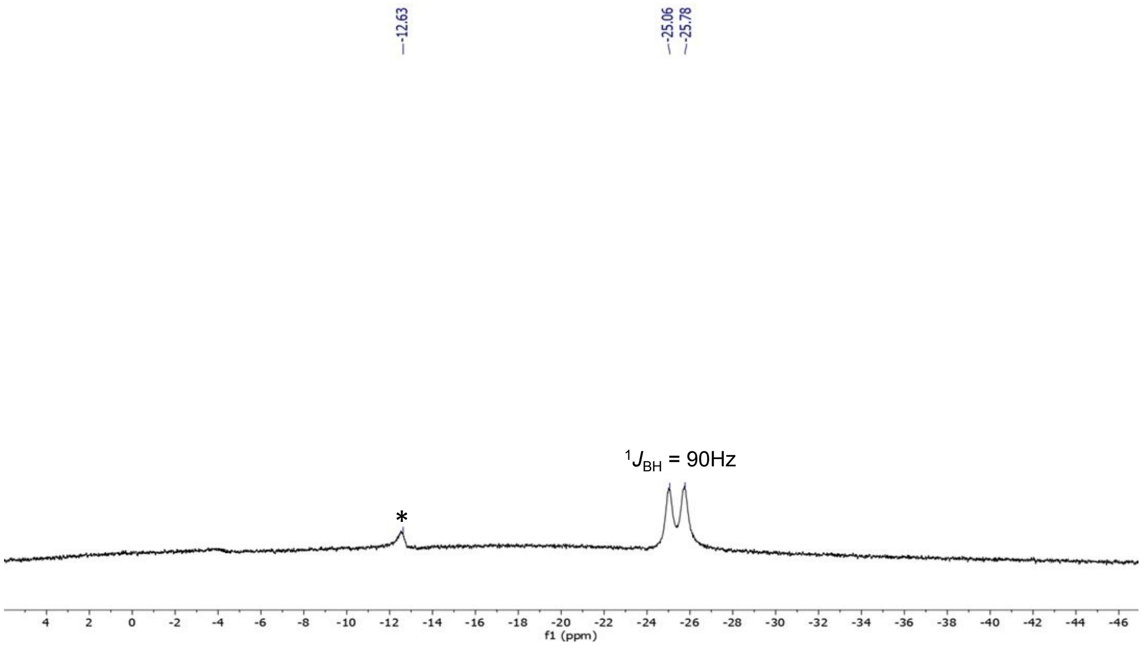

Figure S29. ^{11}B-NMR (CD_2Cl_2, 25 °C, 128 MHz) spectrum of the reaction product of **2b** with $B(C_6F_5)_3$ in CD_2Cl_2. The signal marked with asterisk (*) corresponds to **$[EtB(C_6F_5)_3]^-$**

3.4 Reaction of 1c·BAr$^F_4$ with LiHBEt$_3$. Formation of 2c and 3c.

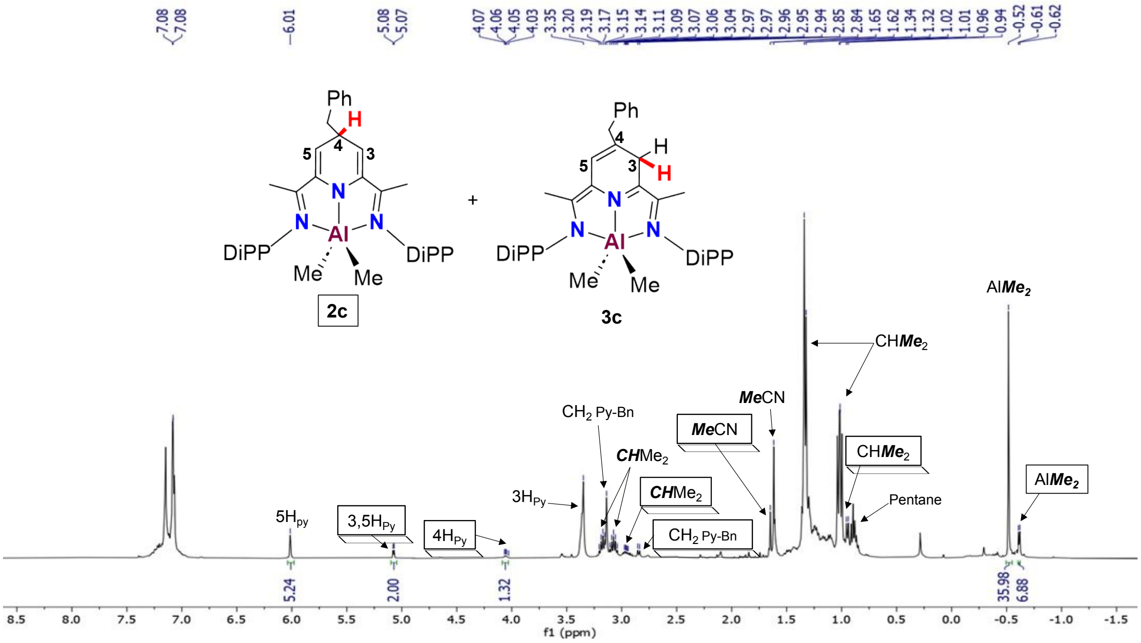

Figure S30. ^{1}**H-NMR** (C_6D_6, 25 °C, 400 MHz) spectrum of the product of the reaction of **1c·BAr$^F_4$** with LiHBEt$_3$.

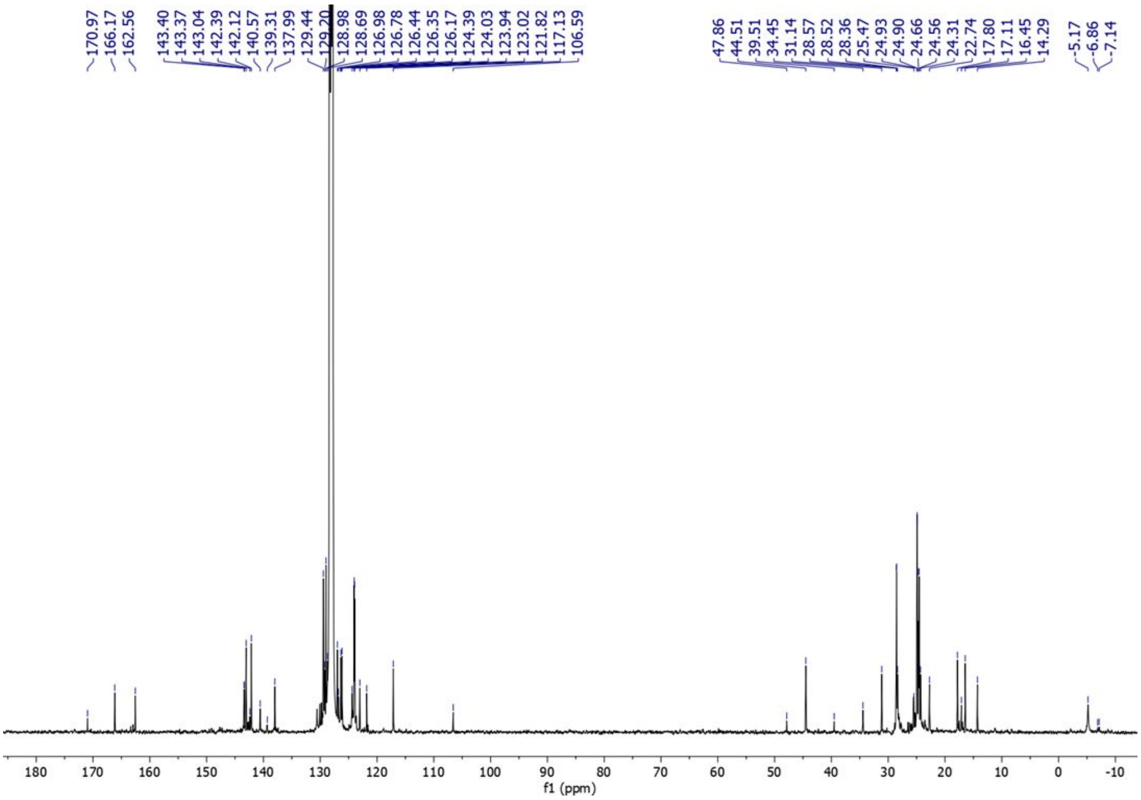

Figure S31. ^{13}**C{^{1}H}-NMR** (C_6D_6, 25 °C, 100 MHz) spectrum of the product of the reaction of **1c·BAr$^F_4$** with LiHBEt$_3$.

3.5 Reaction of the mixture of 2c and 3c with $B(C_6F_5)_3$.

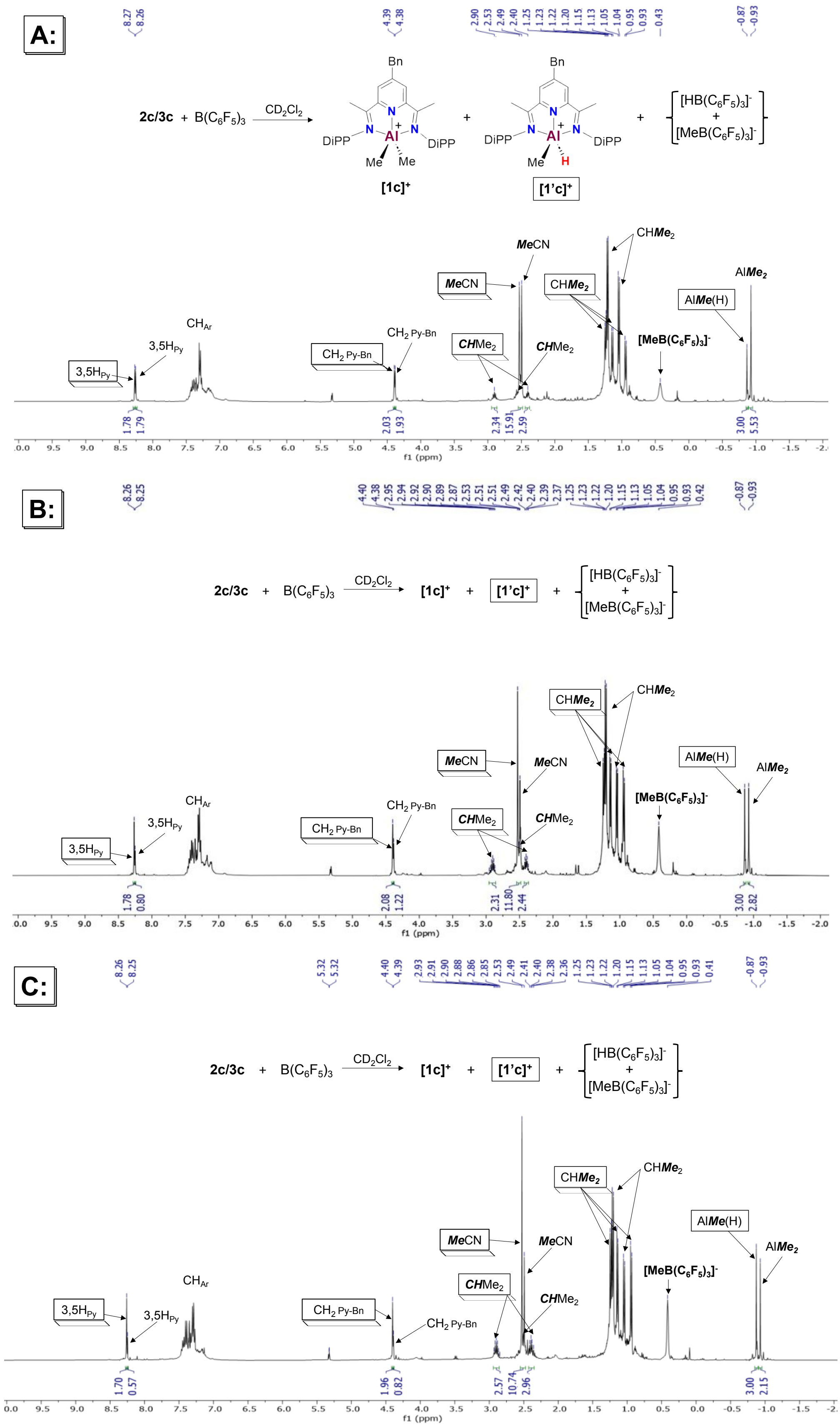

Figure S32. [1]**H-NMR** (CD_2Cl_2, 25 °C, 400 MHz) spectra of the reaction product of the mixture **2c**/**3c** with $B(C_6F_5)_3$ in CD_2Cl_2 after 5 minutes (**A**), 24 hours (**B**) and 48 hours (**C**).

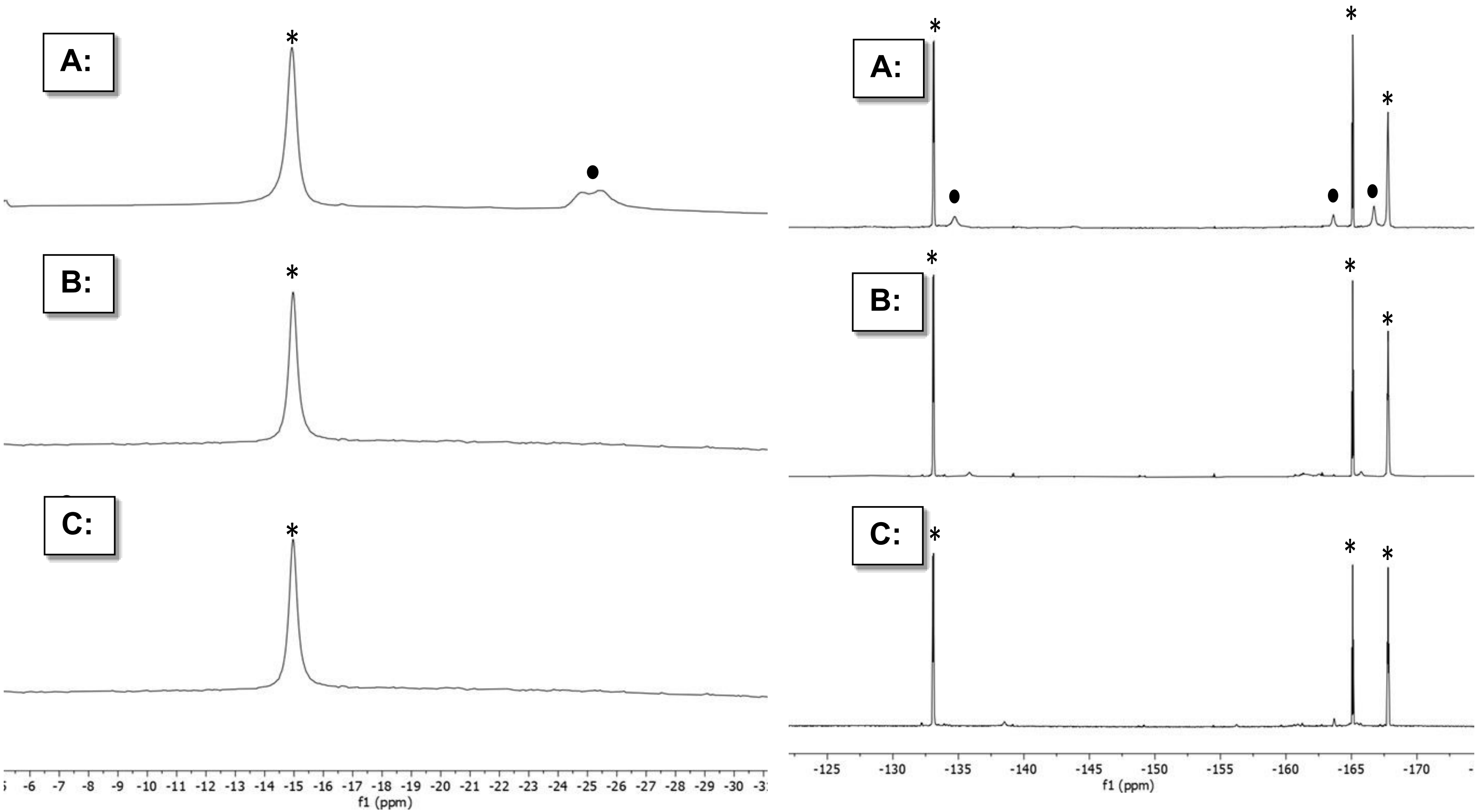

Figure S33. ^{11}**B-NMR (*left*)** (CD_2Cl_2, 25 °C, 128 MHz) and ^{19}**F{^{1}H}-NMR (*right*)** (CD_2Cl_2, 25 °C, 376 MHz) spectra of the reaction product of the mixture **2c**/**3c** with $B(C_6F_5)_3$ in CD_2Cl_2 after 5 minutes (**A**), 24 hours (**B**) and 48 hours (**C**). The signs marked with asterisk (*) correspond to **$[MeB(C_6F_5)_3]^-$**. The signs marked with (●) correspond to **$[HB(C_6F_5)_3]^-$**.

3.6 EPR spectrum

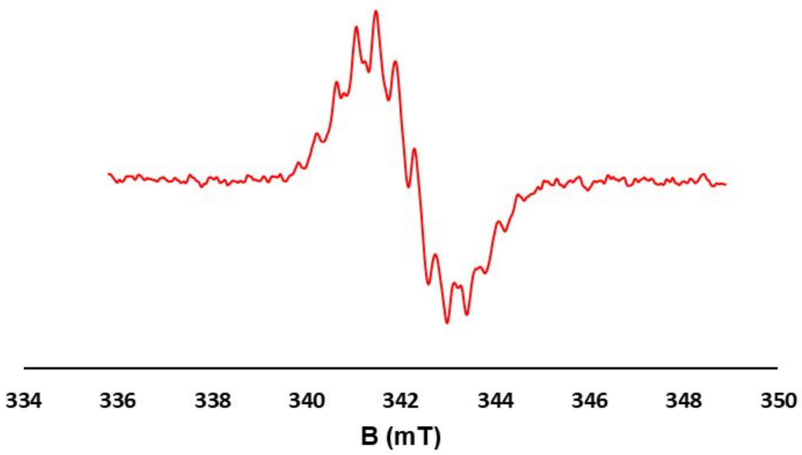

Figure S34. Solution X-band EPR spectrum products of the reaction of the mixture of **2c/3c** with $B(C_6F_5)_3$ after 30 minutes in CD_2Cl_2 at 293 K. Conditions: Frequency = 9.6039 GHz, modulation amplitude = 0.1 mT, microwave power = 0.6310 mW.

3.7 Reaction of 2a with dry O_2.

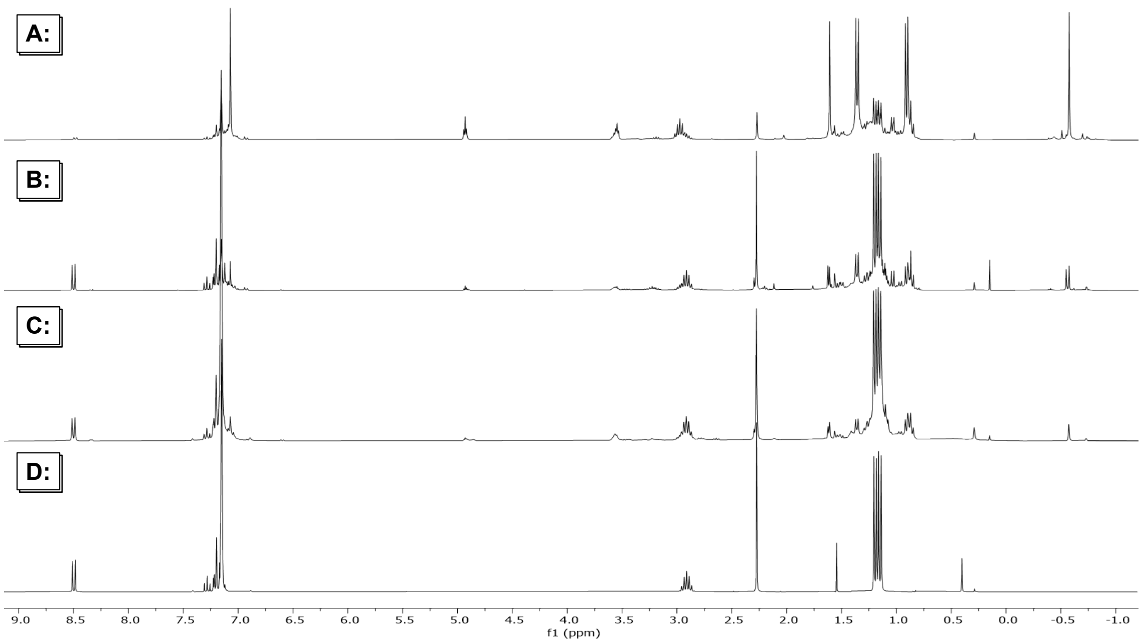

Figure S35. ^{1}H-NMR (C_6D_6, 25 °C, 400 MHz) spectra of the reaction of **2a** with dry O_2. **A:** Complex **2a** before reaction. **B:** Product of the reaction of complex **2a** + O_2 after 24 h at room temperature under N_2. C: Product of the reaction of complex **2a** + O_2 after 24 h at room temperature under N_2 (NMR tube open to air // 30 min at room temperature). **D: ^{1}H-NMR** (C_6D_6, 25 °C, 400 MHz) of isolated ^{DiPP}BIP for comparison.

3.8. Reaction of 2a with MeOH

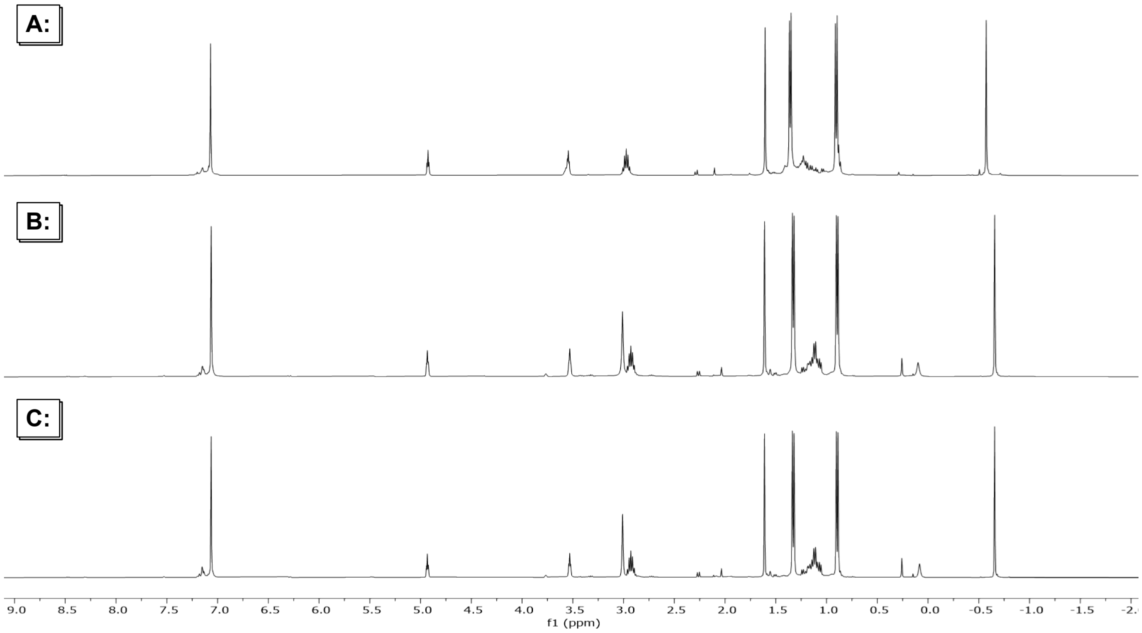

Figure S36. **^{1}H-NMR** (C_6D_6, 25 °C, 400 MHz) spectra of the reaction of **2a** with MeOH. **A:** Complex **2a** before reaction. **B:** Complex **2a** + MeOH after 1 h at room temperature. C: Complex **2a** + MeOH after 24 h at room temperature.

4. X-ray structural characterization

Single-Crystal X-ray Data.

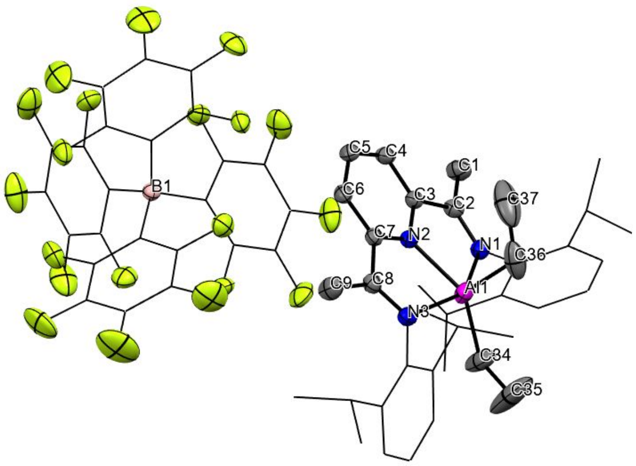

Figure S37. ORTEP representation of the structure of compound **1b·B(C_6F_5)$_4$**. Hydrogen atoms and 2.5 molecules of CH_2Cl_2 have been omitted for clarity. Selected bond lengths (Å) and angles (deg): Al–N(1): 2.2028(16); Al–N(2): 2.0081(16); Al–N(3): 2.1733(16); Al–C(36): 1.968(3); Al–C(34): 1.970(3); N(1)–C(2): 1.282(2); N2–C(3): 1.342(2); N(2)–C(7): 1.342(2); N(3)–C(8): 1.283(2); C(1)–C(2): 1.493(3); C(3)–C(2): 1.488(3); C(3)–C(4): 1.384(2); C(4)–C(5): 1.384(3); C(5)–C(6): 1.387(3); C(6)–C(7): 1.389(3); C(7)–C(8): 1.487(3); C(8)–C(9): 1.486(3); N(2)–Al–N(1): 74.73(6); N(2)–Al–C(36): 102.30(11); N(2)–Al–C(34): 139.64(11); C(34)–Al–C(36): 117.99(14); N(2)–Al–N(3): 75.25(6); N(1)–Al–N(3): 147.22(7).

Table S1. Crystal data and structure refinement for **1b·B(C_6F_5)$_4$**

Empirical formula	$C_{64.5}H_{60}AlBCl_7F_{20}N_3$	
Formula weight	1543.09	
Wavelength	0.71073 Å	
Temperature	193.00 K	
Crystal system	Triclinic	
Space group	P-1	
Unit cell dimensions	a = 10.8030(4) Å	α= 101.7800(10)°
	b = 15.9370(5) Å	β= 100.982(2)°
	c = 21.4760(7) Å	γ = 96.947(2)°
Volume	3503.8(2) Å^3	
Z	2	
Density (calculated)	1.463 g/cm^3	
Absorption coefficient	0.391 mm^{-1}	
F(000)	1570.0	
Crystal size	0.3 x 0.17 x 0.1 mm^3	

Theta range for data collection	3.662 to 56.748
Index ranges	$-14 \le h \le 14$, $-21 \le k \le 21$, $-28 \le l \le 28$
Reflections collected	187686
Independent reflections	17511 [R_{int} = 0.0790, R_{sigma} = 0.0356]
Completeness to theta = 28.374°	99.7 %
Absorption correction	Multi-Scan
Max. and min. transmission	0.746 and 0.598
Data / restraints / parameters	17511/0/814
Goodness-of-fit on F^2	1.027
Final R indexes [I>2sigma(I)]	R_1 = 0.0562, wR_2 = 0.1277
R indexes (all data)	R_1 = 0.0769, wR_2 = 0.1391
Largest diff. peak and hole	0.82/-0.69 Å^{-3}

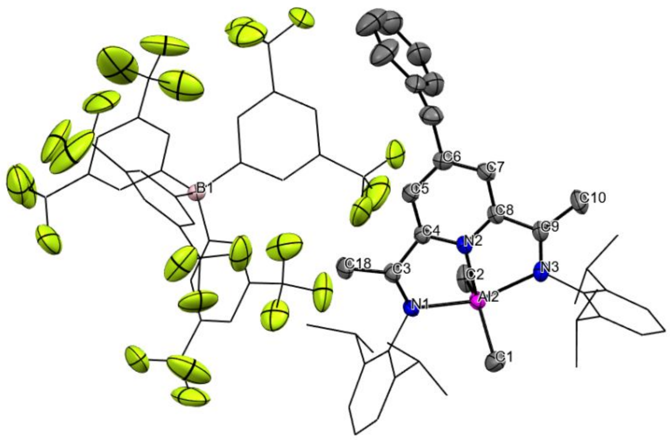

Figure S38. ORTEP representation of the structure of compound **1c·BAr^F$_4$**. Hydrogen atoms have been omitted for clarity. Selected bond lengths (Å) and angles (deg): Al–N(1): 2.167(2); Al–N(2): 2.002(3); Al–N(3): 2.160(3); Al–C(1): 1.946(4); Al–C(2): 1.968(3); N(1)–C(3): 1.278(4); N2–C(4): 1.347(4); N(2)–C(8): 1.336(4); N(3)–C(9): 1.286(4); C(8)–C(9): 1.490(4); C(9)–C(10): 1.490(4); C(8)–C(7): 1.390(4); C(6)–C(7): 1.388(4); C(6)–C(5): 1.396(4); C(5)–C(4): 1.383(4); C(4)–C(3): 1.486(4); C(3)–C(18): 1.498(4); N(2)–Al–N(1): 74.40(9); N(2)–Al–C(1): 142.37(15); N(2)–Al–C(2): 97.48(14); C(1)–Al–C(2): 120.14(18); N(2)–Al–N(3): 75.34(10); N(1)–Al–N(3): 145.02(10).

Table S2. Crystal data and structure refinement for **1c·BAr^F$_4$**.

Empirical formula	$C_{74}H_{67}AlBF_{24}N_3$, (CH_2Cl_2)	
Formula weight	1577.02	
Wavelength	0.71073 Å	
Temperature	193.00 K	
Crystal system	Monoclinic	
Space group	$P2_1/n$	
Unit cell dimensions	a = 12.6090(4) Å	α= 90°
	b = 43.3832(17) Å	β= 96.682(2)°
	c = 13.9209(6) Å	γ = 90°
Volume	7563.3(5) Å^3	
Z	4	
Density (calculated)	1.385 g/cm^3	
Absorption coefficient	0.201 mm^{-1}	
F(000)	3232.0	
Crystal size	0.1 x 0.1 x 0.05 mm^3	
Theta range for data collection	3.756 to 50.728	
Index ranges	$-15 \le h \le 14$, $-52 \le k \le 52$, $-16 \le l \le 16$	

Reflections collected	105225
Independent reflections	13858 [R_{int} = 0.0733, R_{sigma} = 0.0440]
Completeness to theta = 25.364°	99.9 %
Absorption correction	Multi-Scan
Max. and min. transmission	0.745 and 0.605
Data / restraints / parameters	13858 /300/1052
Goodness-of-fit on F^2	1.013
Final R indexes [I>2sigma(I)]	R_1 = 0.0741, wR_2 = 0.1774
R indexes (all data)	R_1 = 0.1048, wR_2 = 0.1958
Largest diff. peak and hole	0.80/-0.50 Å^{-3}

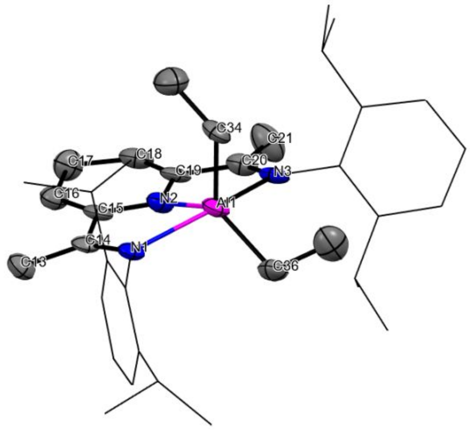

Figure S39. ORTEP representation of the structure of compound **2b**. Hydrogen atoms have been omitted for clarity. Selected bond lengths (Å) and angles (deg): Al–N(1): 2.218(5); Al–N(2): 1.893(5); Al–N(3): 2.238(5); Al–C(36): 1.993(8); Al–C(34): 1.981(6); N(1)–C(14): 1.274(7); N2–C(15): 1.383(7); N(2)–C(19): 1.380(8); N(3)–C(20): 1.299(8); C(14)–C(15): 1.451(8); C(13)–C(14): 1.515(8); C(15)–C(16): 1.346(9); C(16)–C(17): 1.485(9); C(17)–C(18): 1.505(8); C(19)–C(18): 1.330(9); C(19)–C(20): 1.469(7); C(20)–C(21): 1.510(9); N(2)–Al–N(1): 76.0(2); N(2)–Al–C(36): 136.3(2); N(2)–Al–C(34): 112.7(2); C(34)–Al–C(36): 111.0(3); N(2)–Al–N(3): 76.5(2); N(1)–Al–N(3): 150.9(2).

Table S3. Crystal data and structure refinement for **2b.**

Empirical formula	$C_{37}H_{54}N_3Al$	
Formula weight	567.81	
Wavelength	0.71073 Å	
Temperature	193.00 K	
Crystal system	Monoclinic	
Space group	C2/c	
Unit cell dimensions	a = 43.963(9) Å	α= 90°
	b = 10.0118(18) Å	β= 110.364(9)°
	c = 16.745(3) Å	γ = 90°
Volume	6910(2) $Å^3$	
Z	8	
Density (calculated)	1.092 g/cm^3	
Absorption coefficient	0.086 mm^{-1}	
F(000)	2480.0	
Crystal size	0.12 x 0.07 x 0.02 mm^3	
Theta range for data collection	4.742 to 50.724	
Index ranges	$-52 \le h \le 49$, $0 \le k \le 12$, $0 \le l \le 20$	
Reflections collected	6226	

Independent reflections	6226 [R_{int} = ?, R_{sigma} = 0.01271]
Completeness to theta = 25.362°	98.3 %
Absorption correction	Multi-Scan
Max. and min. transmission	0.745 and 0.566
Data / restraints / parameters	6226/0/383
Goodness-of-fit on F^2	1.194
Final R indexes [I>2sigma(I)]	R_1 = 0.1285, wR_2 = 0.2436
R indexes (all data)	R_1 = 0.2000, wR_2 = 0.2737
Largest diff. peak and hole	0.41/-0.34 Å^{-3}
